# Circulating tumor DNA-guided treatment with pertuzumab plus trastuzumab for *HER2*-amplified metastatic colorectal cancer: a phase 2 trial

**DOI:** 10.1038/s41591-021-01553-w

**Published:** 2021-11-11

**Authors:** Yoshiaki Nakamura, Wataru Okamoto, Takeshi Kato, Taito Esaki, Ken Kato, Yoshito Komatsu, Satoshi Yuki, Toshiki Masuishi, Tomohiro Nishina, Hiromichi Ebi, Kentaro Sawada, Hiroya Taniguchi, Nozomu Fuse, Shogo Nomura, Makoto Fukui, Seiko Matsuda, Yasutoshi Sakamoto, Hiroshi Uchigata, Kana Kitajima, Naomi Kuramoto, Takashi Asakawa, Steve Olsen, Justin I. Odegaard, Akihiro Sato, Satoshi Fujii, Atsushi Ohtsu, Takayuki Yoshino

**Affiliations:** 1 Department of Gastroenterology and Gastrointestinal Oncology, National Cancer Center Hospital East, Kashiwa, Japan; 2 Translational Research Support Section, National Cancer Center Hospital East, Kashiwa, Japan; 3 Cancer Treatment Center, Hiroshima University Hospital, Hiroshima, Japan; 4 Department of Surgery, National Hospital Organization Osaka National Hospital, Osaka, Japan; 5 Department of Gastrointestinal and Medical Oncology, National Hospital Organization Kyushu Cancer Center, Fukuoka, Japan; 6 Department of Head and Neck, Esophageal Medical Oncology, National Cancer Center Hospital, Tokyo, Japan; 7 Department of Cancer Center, Hokkaido University Hospital, Sapporo, Japan; 8 Department of Gastroenterology and Hepatology, Hokkaido University Hospital, Sapporo, Japan; 9 Department of Clinical Oncology, Aichi Cancer Center Hospital, Nagoya, Japan; 10 Gastrointestinal Medical Oncology, National Hospital Organization Shikoku Cancer Center, Matsuyama, Japan; 11 Division of Molecular Therapeutics, Aichi Cancer Center Research Institute, Nagoya, Japan; 12 Department of Medical Oncology, Kushiro Rosai Hospital, Kushiro, Japan; 13 Clinical Research Support Office, National Cancer Center Hospital East, Kashiwa, Japan; 14 Chugai Pharmaceutical Co., Ltd, Tokyo, Japan; 15 Clinical and Medical Affairs, Guardant Health AMEA, Redwood City CA, USA; 16 Clinical Development, Guardant Health, Redwood City CA, USA; 17 Division of Pathology, Exploratory Oncology Research & Clinical Trial Center, National Cancer Center, Kashiwa, Japan; 18 Department of Molecular Pathology, Yokohama City University Graduate School of Medicine, Yokohama, Japan

**Keywords:** Translational research, Predictive markers

## Abstract

The applicability of circulating tumor DNA (ctDNA) genotyping to inform enrollment of patients with cancer in clinical trials has not been established. We conducted a phase 2 trial to evaluate the efficacy of pertuzumab plus trastuzumab for metastatic colorectal cancer (mCRC), with human epidermal growth factor receptor 2 (*HER2*) amplification prospectively confirmed by tumor tissue or ctDNA analysis (UMIN000027887). *HER2* amplification was confirmed in tissue and/or ctDNA in 30 patients with mCRC. The study met the primary endpoint with a confirmed objective response rate of 30% in 27 tissue-positive patients and 28% in 25 ctDNA-positive patients, as compared to an objective response rate of 0% in a matched real-world reference population treated with standard-of-care salvage therapy. Post hoc exploratory analyses revealed that baseline ctDNA genotyping of *HER2* copy number and concurrent oncogenic alterations adjusted for tumor fraction stratified patients according to efficacy with similar accuracy to tissue genotyping. Decreased ctDNA fraction 3 weeks after treatment initiation associated with therapeutic response. Pertuzumab plus trastuzumab showed similar efficacy in patients with mCRC with *HER2* amplification in tissue or ctDNA, showing that ctDNA genotyping can identify patients who benefit from dual-HER2 blockade as well as monitor treatment response. These findings warrant further use of ctDNA genotyping in clinical trials for *HER2-*amplified mCRC, which might especially benefit patients in first-line treatment.

## Abstract (web summary)

The multicenter phase 2 TRIUMPH trial shows the utility of ctDNA genotyping as a screening platform to select patients with *HER2*-amplified metastatic colorectal cancer who benefit from dual-HER2 blockade with trastuzumab and pertuzumab

## Main

ctDNA is routinely used to detect genomic alterations in patients with advanced solid tumors^1,2^. We established a ctDNA-based screening study, called GOZILA, in Japan for matching patients with advanced gastrointestinal cancer to affiliated targeted therapy trials based on genotyping results. We showed that ctDNA-based genotyping accelerated enrollment in trials while maintaining equivalent efficacy compared to tissue-based genotyping^3^. Nevertheless, few clinical trials have prospectively evaluated the efficacy of ctDNA genotyping^4–6^, particularly for gene amplifications.

Based on a large-scale nationwide screening platform—the SCRUM-Japan GI-SCREEN and the GOZILA networks—we designed TRIUMPH, a phase 2 trial to evaluate the efficacy of pertuzumab plus trastuzumab in patients with mCRC with *RAS* wild-type and *HER2* amplification prospectively confirmed by tumor tissue or ctDNA analysis. Furthermore, real-world clinical outcomes for patients with *RAS* wild-type and *HER2*-amplified mCRC treated with non-HER2-targeted standard-of-care therapies were assessed as a reference using the SCRUM-Japan Registry, which is an observational cohort study of real-world data from patients with advanced solid tumors. Exploratory analyses were performed to evaluate the utility of ctDNA genotyping to predict treatment efficacy, monitor the response and identify the resistance mechanisms according to a pre-specified protocol.

**Fig. 1:**
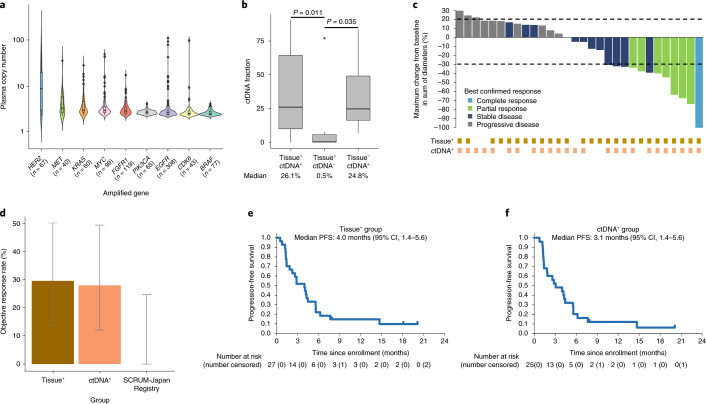
Clinical efficacy of pertuzumab plus trastuzumab for *HER2*-amplified mCRC. **a**, pCN of the ten most frequent gene amplifications in 1,107 patients with mCRC enrolled in GOZILA between January 2018 and March 2020. The pCN of *HER2* amplification (median, 8.6) was significantly higher than that of each other gene amplification (two-sided *P* < 0.0001 for each, Mann–Whitney *U-*test). The boxes represent 25th–75th percentiles; center lines indicate the median; whiskers extend to the maximum and minimum values within 1.5× of the interquartile range; and dots indicate outliers. **b**, ctDNA fraction of patients with tissue^+^ctDNA^+^ (*n* = 32), tissue^+^ctDNA^−^ (*n* = 7) and tissue^−^ctDNA^+^ (*n* = 6) for *HER2* amplification. The ctDNA fraction of those with tissue^+^ctDNA^−^ (median, 0.5%) was significantly lower than that with tissue^+^ctDNA^+^ (two-sided *P* = 0.011, Mann–Whitney *U*-test) or tissue^−^ctDNA^+^ (two-sided *P* = 0.035, Mann–Whitney *U*-test). The boxes represent 25th–75th percentiles; center lines indicate the median; whiskers extend to the maximum and minimum values within 1.5× of the interquartile range; and dots indicate outliers. **c**, Waterfall plot showing the change in the sum of the longest diameters of lesions from the baseline to the best post-baseline investigator assessment. Allocation to tissue^+^ or ctDNA^+^ group is shown by the boxes at the bottom of the figure. **d**, ORR in tissue^+^ (*n* = 27), ctDNA^+^ (*n* = 25) and SCRUM-Japan Registry (*n* = 13) patients. Error bars represent 95% CIs. **e**, Kaplan–Meier plots of PFS in the tissue^+^ group. Vertical lines denote patients who were censored. **f**, Kaplan–Meier plots of PFS in the tissue^+^ group.

**Extended Data Fig. 1:**
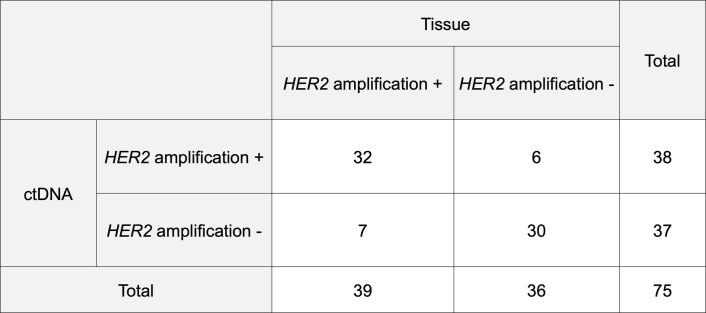
*HER2* amplification between tissue and ctDNA. In 75 patients tested by both HER2-Screening and GOZIA studies, the positive percent agreemen, negative percent agreement, and the overall agreement for *HER2* amplification between tissue and ctDNA analysis was 82%, 83%, and 83%, respectively.

**Extended Data Fig. 2:**
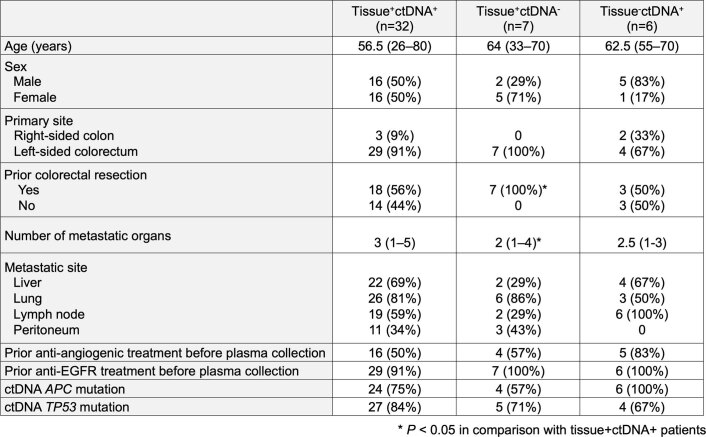
Baseline characteristics of patients receiving screening studies by tissue and ctDNA HER2 status. Data are n (%) or median (range). Tissue^+^ctDNA^−^ patients more commonly received prior colorectal resection (two-sided *P* = 0.036, Fisher’s exact test) and had higher number of metastatic organs (two-sided *P* = 0.040, Mann-Whitney U test) than tissue^+^ctDNA^−^ patients. ctDNA, circulating tumor DNA.

Two screening studies—HER2-Screening (immunohistochemistry (IHC) and fluorescence in situ hybridization (FISH)) and GOZILA (plasma next-generation sequencing (NGS) analysis)—were conducted to centrally confirm tissue and ctDNA *HER2* amplification, respectively. Between December 2017 and March 2020, tissue *HER2* amplification was confirmed by IHC and FISH in 56 (IHC 3+ for 50 and FISH-positive for 56) of 147 patients in HER2-Screening. Between January 2018 and March 2020, ctDNA *HER2* amplification was confirmed in 66 of 1,107 patients with mCRC in GOZILA. *HER2* amplification events showed the highest median plasma copy number (pCN) of all ctDNA gene amplifications, suggesting the role of *HER2* amplification as a driver and targetable alteration in mCRC (Fig. 1a). Seventy-five patients were tested by both screening studies. Of these, seven (18%) of 39 patients with tissue *HER2* amplification had no ctDNA *HER2* amplification (tissue^+^ctDNA^−^), whereas tissue *HER2* amplification was not confirmed in six (16%) of 38 patients with ctDNA *HER2* amplification (tissue^−^ctDNA^+^) (Extended Data Fig. 1). Patients with tissue^+^ctDNA^−^ had a significantly lower ctDNA fraction than patients with tissue^+^ctDNA^+^ and patients with tissue^−^ctDNA^+^ (Fig. 1b). The low tumor shedding in patients with tissue^+^ctDNA^−^ might be associated with low tumor volume, suggested by more common prior colorectal resection and fewer metastatic organs shown in this population (Extended Data Fig. 2). In all patients with tissue^−^ctDNA^+^ tumors, tumor tissue samples were obtained before the initiation of anti-epidermal growth factor receptor (EGFR) therapy, whereas plasma samples were collected after progression on anti-EGFR therapy.

**Extended Data Fig. 3:**
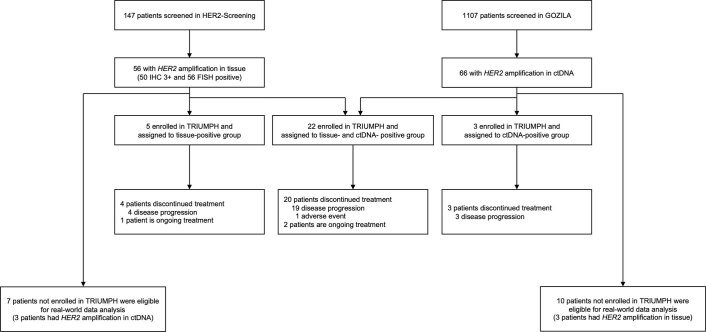
Screening and enrollment. Tissue and ctDNA screening were separately conducted for patients with metastatic colorectal cancer. Patients with *HER2* amplification confirmed by either of the screening studies were enrolled in the TRIUMPH study. Patients who were not enrolled in the TRIUMPH due to any reason, such as out of enrollment period, or who are at the site not participating in TRIUMPH were enrolled in the SCRUM-Japan Registry.

**Extended Data Fig. 4:**
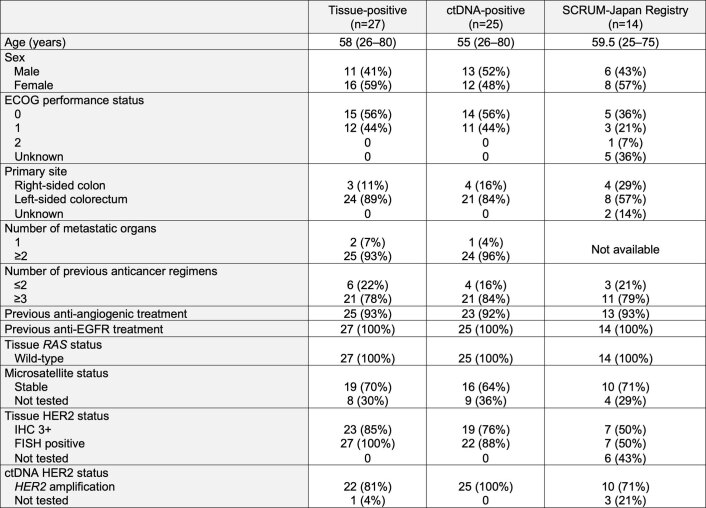
Baseline characteristics of patients in the TRIUMPH and SCRUM-Japan Registry. Summary of baseline characteristics of patients in the TRIUMPH and SCRUM-Japan Registry. Data are n (%) or median (range). Right-sided colon cancer was defined as cancer that is located in the cecum, ascending colon, or transverse colon, and left-sided colorectum cancer was defined as cancer that is in the descending colon, sigmoid colon, rectosigmoid colon, or rectum. ctDNA, circulating tumor DNA; ECOG, Eastern Cooperative Oncology Group; IHC, immunohistochemistry; FISH, fluorescence in situ hybridization.

In total, 30 patients with *HER2* amplification were enrolled in TRIUMPH between 24 January 2018 and 29 July 2019 (Extended Data Fig. 3). Most patients were overlapped in both tissue^+^ and ctDNA^+^ groups because the prevalence of *HER2* amplification newly identified after anti-EGFR therapy was less than expected, as previously reported^7^. *HER2* amplification was confirmed in both tissue and ctDNA for 22 of 30 patients (in tissue alone for five and in ctDNA alone for three); therefore, 27 and 25 patients were assigned to tissue^+^ and ctDNA^+^ groups, respectively. One patient with *HER2* amplification confirmed in HER2-Screening but for whom a baseline ctDNA result was unavailable was assigned only to the tissue^+^ group. The median follow-up was 9.2 months in patients with tissue^+^ and 7.6 months in patients with ctDNA^+^. Baseline characteristics are shown in Extended Data Fig. 4.

**Extended Data Fig. 5:**
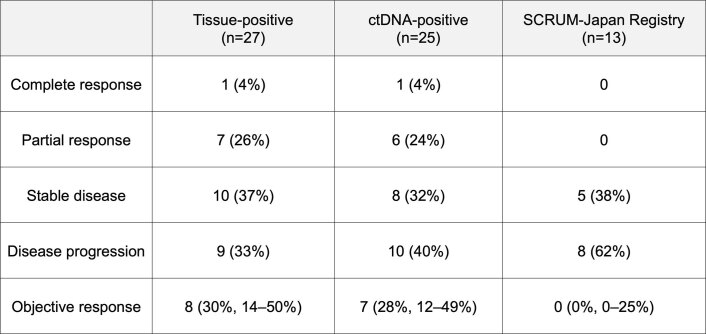
Best confirmed response. Summary of best confirmed response by investigator assessment to pertuzumab plus trastuzumab and standard-of-care treatment in SCRUM-Japan Registry. Data are n (%) or median (range). Right-sided colon cancer was d Data are n (%) or n (%, 95% CI).

**Extended Data Fig. 6:**
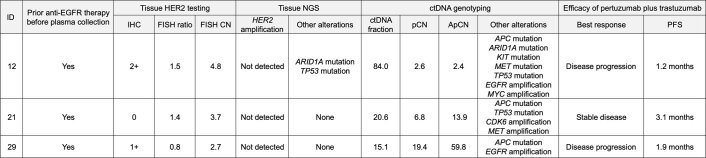
HER2 status and efficacy of pertuzumab plus trastuzumab in three patients with *HER2* amplification in ctDNA alone. All patients received prior anti-EGFR therapy before plasma collection. No HER2 amplification was identified in immunohistochemistry (IHC), fluorescence in situ hybridization (FISH), or next generation sequencing (NGS) tests. Some pathogenic alterations not found in tissue were identified by ctDNA genotyping. No clinical benefit was achieved. pCN, plasma copy number; ApCN, adjusted pCN; and PFS, progression-free survival.

The study met the primary endpoint, with eight (30%) of 27 patients with tissue^+^ achieving a confirmed investigator-assessed objective response (95% confidence interval (CI), 14–50%), including one complete response, and seven (28%) of 25 patients with ctDNA^+^ achieving a confirmed objective response (95% CI, 12–49%), including one complete response, which were more than the five required to reject the null hypothesis (Fig. 1c,d and Extended Data Fig. 5). Median duration of response was 12.1 months (95% CI, 2.8 to not reached) in patients with tissue^+^ and 8.1 months (95% CI, 2.8 to not reached) in patients with ctDNA^+^. The single patient who was ctDNA^−^ showed a *HER2* pCN below the limit of detection due to a very low ctDNA fraction; when adjusted for ctDNA fraction, the pCN was high, reaching 26.5 copies. No objective response was confirmed in three patients with *HER2* amplification identified in ctDNA alone. HER2 status and the efficacy of these patients are shown in Extended Data Fig. 6. Lack of tissue *HER2* amplification (confirmed using NGS, IHC and FISH analysis) and anti-EGFR therapy before ctDNA analysis in all patients suggested that the intratumoral heterogeneous *HER2*-amplified clones or acquired *HER2* amplification were driven by the evolutionary pressures created by anti-EGFR therapy.

The real-world reference data comprised 14 eligible patients from the SCRUM-Japan Registry with wild-type *RAS* and *HER2* amplification confirmed by HER2-Screening or GOZILA but who were not enrolled in TRIUMPH. These patients had been previously treated with fluoropyrimidine, oxaliplatin and irinotecan and anti-EGFR therapy. The most commonly used first salvage-line treatment was trifluridine/tipiracil with or without bevacizumab (Supplementary Table 1). Of 13 evaluable patients, none had a confirmed objective response (0%, 95% CI, 0–25%) (Fig. 1d and Extended Data Fig. 5).

**Extended Data Fig. 7:**
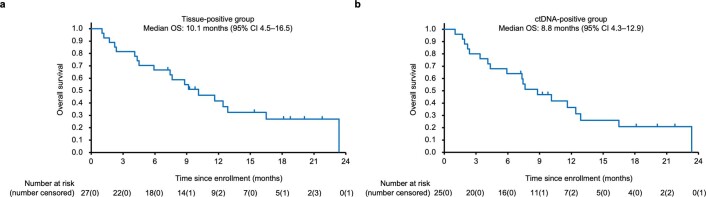
Overall survival. (**a**) Kaplan-Meier plots of overall survival (OS) in tissue-positive group. Vertical lines denote censored patients. (**b**) Kaplan-Meier plots of overall survival (OS) in ctDNA-positive group.

Median progression-free survival (PFS) was 4.0 months (95% CI, 1.4–5.6) and 3.1 months (95% CI, 1.4–5.6) in the tissue^+^ and ctDNA^+^ groups, respectively (Fig. 1e,f). Median overall survival (OS) was 10.1 months (95% CI, 4.5–16.5) and 8.8 months (95% CI, 4.3–12.9) in the tissue^+^ and ctDNA^+^ groups, respectively (Extended Data Fig. 7). Other efficacy endpoints are summarized in Supplementary Table 2. Treatment-related adverse events occurred in 24 (80%) of 30 patients, with the most common being infusion-related reactions, diarrhea, stomatitis and malaise (Supplementary Table 3). Grade 3 treatment-related adverse events occurred in three (10%) patients. No unexpected or treatment-related deaths occurred.

**Fig. 2:**
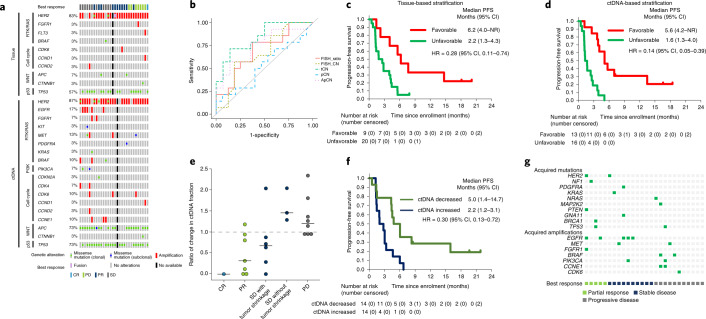
Baseline, on-treatment and on-progression biomarker analysis for treatment with pertuzumab plus trastuzumab. **a**, Baseline alterations of known drivers by tissue or ctDNA genotyping. Patients are listed from left to right in order of increasing change in the sum of the longest diameters of lesions from the baseline to the best post-baseline investigator assessment. **b**, Receiver operating characteristics to predict the clinical benefit by pertuzumab plus trastuzumab using baseline HER2 status: FISH *HER2*/*CEP17* ratio, FISH *HER2* CN, tissue NGS copy number (tCN), pCN and ApCN. **c**, Kaplan–Meier plots of PFS for patients with tumors harboring tissue-based favorable factors (tissue CN ≥ 68.7 and no concurrent RTK/RAS/PI3K alterations) versus those lacking favorable factors. **d**, Kaplan–Meier plots of PFS for patients with tumors harboring ctDNA-based favorable factors (ApCN ≥ 16.7 and no concurrent RTK/RAS/PI3K alterations) versus those lacking favorable factors. **e**, Proportional change in ctDNA fraction by tumor response: complete response (CR) (*n* = 1), partial response (PR) (*n* = 7), stable disease (SD) with tumor shrinkage (*n* = 6), SD without tumor shrinkage (*n* = 3) and progressive disease (PD) (*n* = 8). **f**, Kaplan–Meier plots of PFS for patients with decreased versus increased ctDNA fraction. **g**, Acquired alterations at disease progression. NR, not reached.

**Extended Data Fig. 8:**
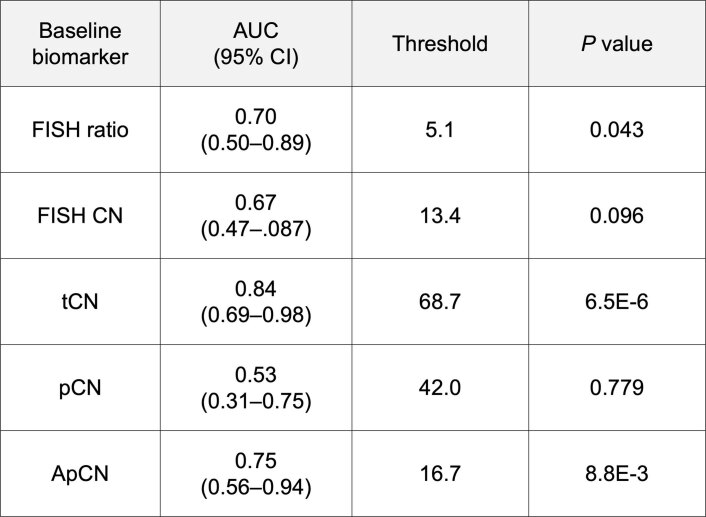
Receiver operating characteristics analysis of baseline HER2 status. Summary of receiver operating characteristics (ROC) analysis of baseline HER2 status to predict the clinical benefit by pertuzumab plus trastuzumab. Baseline HER2 status included fluorescence in situ hybridization (FISH) *HER2*/*CEP17* ratio, FISH *HER2* copy number (CN), tissue NGS copy number (tCN), plasma copy number (pCN), and adjusted pCN (ApCN). The two-sided *P* value tests the null hypothesis that the area under the ROC curve is 0.5. Adjustments were not made for multiple comparisons. Threshold is determined as the point of the ROC curve which is closest to the perfect (1,0) coordinate.

**Extended Data Fig. 9:**
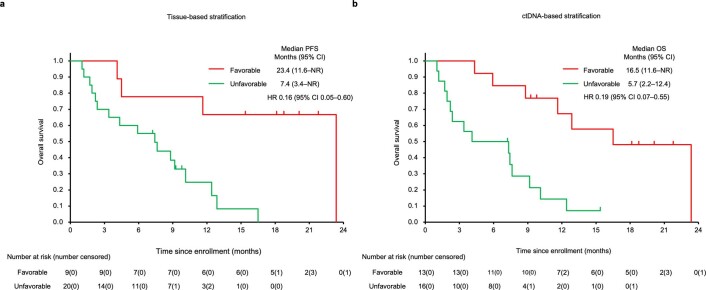
Overall survival according to tissue or ctDNA-based stratification. (**a**) Kaplan-Meier plots of OS for patients with tumors harboring tissue-based favorable factors (tissue CN ≥68.7 and no concurrent RTK/RAS/PI3K alterations) versus those lacking favorable factors. (**b**) Kaplan-Meier plots of OS for patients with tumors harboring ctDNA-based favorable factors (ApCN ≥16.7 and no concurrent RTK/RAS/PI3K alterations) versus those lacking favorable factors.

Because plasma ctDNA can reflect the genomic profile at the time of treatment initiation, ctDNA genotyping can potentially identify patients who are more or less likely to benefit from specific therapies. To investigate the association between baseline tissue or ctDNA genomic profiles and treatment efficacy, we first performed a post hoc exploratory analysis associating radiographic responses with specific genomic alterations. Co-alterations of oncogenes, such as *HER2* and *BRAF*, were detected only in a small number of non-responders by tissue NGS (Fig. 2a). On the other hand, baseline ctDNA genotyping showed that amplifications or clonal mutations of genes related to receptor tyrosine kinase (RTK)/RAS and PI3K, which comprise previously described resistance mechanisms to HER2-targeted therapy in breast cancer and gastric cancer^8,9^, were markedly enriched in non-responders (Fig. 2a and Supplementary Table 4). In total, in baseline samples from non-responders, concurrent alterations in RTK/RAS/PI3K-related genes were identified in 19% (4/21) by tissue versus 67% (14/21) by ctDNA genotyping (*P* = 0.004). Next, we assessed the association between the baseline HER2 status in tissue and ctDNA and clinical benefits as defined by radiographic response or stable disease at four or more months. Among tissue-based HER2 status, *HER2* copy number (CN) by an NGS analysis (green dashed line in Fig. 2b) was more significantly associated with the clinical benefit than *HER2*/*CEP17* ratio or HER2 CN by FISH (area under the receiver operating characteristics curve (AUROC) = 0.84, *P* < 0.001; Fig. 2b and Extended Data Fig. 8). As expected due to the confounding effects of tumor fraction, absolute plasma ctDNA CN was not associated with the clinical benefit, whereas the association of the clinical benefit with tumor-fraction-adjusted plasma CN (ApCN) was similar to that of tissue NGS (AUROC = 0.75, *P* = 0.009; Fig. 2b and Extended Data Fig. 8). We then compared survival outcomes according to concurrent clonal alterations in RTK/RAS/PI3K-related genes and the threshold of tissue NGS CN for tissue genotyping or ApCN for ctDNA genotyping determined by ROC analysis. Patients with no concurrent oncogenic alterations and a CN above the threshold showed significantly better PFS (median in those with versus without favorable factors, tissue-based, 6.2 versus 2.2 months, hazard ratio (HR) = 0.28 (95% CI, 0.11–0.74); ctDNA-based, 5.6 versus 1.6 months, HR = 0.14 (95% CI, 0.05–0.39); Fig. 2c,d) and OS (median, tissue-based, 23.4 versus 7.4 months, HR = 0.17 (95% CI, 0.05–0.60); ctDNA-based, 16.5 versus 5.7 months, HR = 0.19 (95% CI, 0.07–0.55); Extended Data Fig. 9), although ctDNA-based stratification showed better HRs for PFS despite the inclusion of more patients with favorable factors. These findings indicate that baseline ctDNA genomic profiling can more accurately identify patients who will benefit from pertuzumab plus trastuzumab.

**Extended Data Fig. 10:**
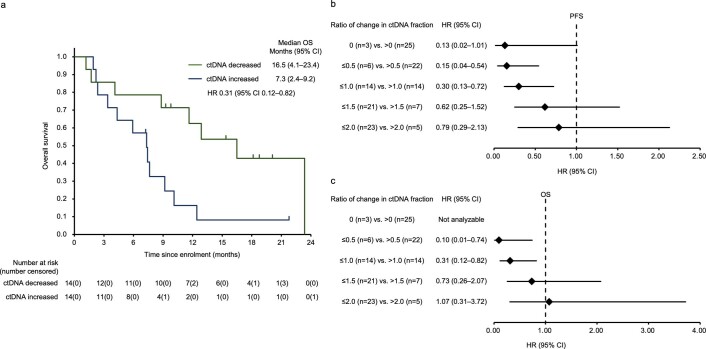
Survival outcomes by change in ctDNA fraction. (**a**) Kaplan-Meier plots of OS for patients with decreased versus increased ctDNA fraction. (**b**) Forest plots depicting the association between proportional change in ctDNA fraction and PFS. The dot and error bars represent the point estimate of hazard ratio and 95% confidence intervals, respectively. (**c**) Forest plots depicting the association between proportional change in ctDNA fraction and OS. The dot and error bars represent the point estimate of hazard ratio and 95% confidence intervals, respectively.

To evaluate whether on-treatment changes in ctDNA could assist predicting clinical benefit, changes in ctDNA fraction in the first 3 weeks after the initiation of the study treatment were compared with the best confirmed response in 28 patients for whom samples from both time points were available. The median proportional change in ctDNA fraction was less than 1.0 in patients who achieved a confirmed response or tumor shrinkage (0.00 for complete response, 0.32 for partial response and 0.68 for stable disease with tumor shrinkage), whereas patients with tumor growth showed increasing (median change, 1.46 for stable disease without tumor shrinkage and 1.21 for progressive disease) ctDNA fractions, respectively (Fig. 2e). Decreased ctDNA fraction was significantly associated with superior PFS (HR = 0.30 (95% CI, 0.13–0.72)) and OS (HR = 0.31 (95% CI, 0.12–0.82)), and there was a monotonic relationship between the change in ctDNA fraction and survival outcomes (Fig. 2f and Extended Data Fig. 10).

To identify genomic mechanisms of acquired resistance to pertuzumab plus trastuzumab in *HER2*-amplified mCRC, results of ctDNA analysis from plasma samples collected after disease progression were compared to those collected before treatment. At least one actionable alteration emerged after disease progression in 16 (62%) of 26 patients (Fig. 2g). Notably, four (80%) of five patients who achieved a response had an actionable acquired alteration. These alterations were enriched in genes related to the RTK/RAS and PI3K pathways, similar to primary resistance. *HER2* amplification was lost in four patients.

This phase 2 trial met its primary endpoint, showing anti-tumor activity of pertuzumab plus trastuzumab with a confirmed objective response rate (ORR) of 30% and 28% in patients with mCRC harboring *HER2* amplification prospectively confirmed by tumor tissue or ctDNA analysis, respectively. The efficacy and safety were similar to those previously reported^10^. No responses to non-HER2-targeted therapy were seen in a real-world reference, consistent with the limited efficacy of salvage-line treatment reported in pivotal trials^11,12^. Our large-scale SCRUM-Japan GI-SCREEN and GOZILA networks allowed evaluation of the efficacy of dual-HER2 blockade and standard-of-care in patients with *HER2*-amplified mCRC prospectively confirmed by tissue and ctDNA analysis, in parallel.

Discordance of *HER2* amplifications between tissue testing and ctDNA genotyping were observed in some patients; however, in all patients with tissue^−^ctDNA^+^, tissue samples were obtained before anti-EGFR therapy, whereas blood was drawn after. As *HER2* amplification is known to be acquired after progression on anti-EGFR therapy^13,14^, the tissue^−^ctDNA^+^ population is likely due to this study design artifact rather than true discordance. In contrast, the tissue^+^ctDNA^−^ population is likely true discordance due to low tumor shedding because these patients showed markedly lower ctDNA fractions. In clinical practice, however, the 10–20% false-negative rate associated with ctDNA-based genotyping is overwhelmingly outweighed by the increase in addressable population owing to genotyping success rates and rapidity^3^. Moreover, although our study was not designed to compare populations, it also reports similar clinical efficacy. Overall, these findings suggest that ctDNA analysis can identify patients with *RAS* wild-type and *HER2*-amplified mCRC who benefit from dual-HER2 blockade as well as conventional tissue analysis.

In addition to selecting patients who benefit from dual-HER2 blockade, our trial suggested additional advantages of ctDNA analysis. First, baseline ctDNA genotyping showed an association of low ApCN and specific concurrent oncogenic alterations with lower efficacy; however, more than half of oncogenic alterations found in baseline ctDNA were not identified in archival tissue NGS testing, suggesting that genotyping is necessary at the time of treatment initiation to best identify patients who will benefit from dual-HER2 blockade by capturing acquired alterations throughout systemic therapy using ctDNA analysis^15^. Second, decreased ctDNA fraction shortly after treatment initiation strongly predicted radiographic response. No previous studies reported the association between ctDNA changes and therapeutic response in *HER2*-amplified mCRC. Our results show the potential clinical utility of immediate changes in ctDNA fraction in predicting patient benefit from pertuzumab plus trastuzumab. Finally, ctDNA analysis after disease progression identified potential acquired resistance alterations in common oncogenic pathways. These resistance mechanisms might inform salvage treatment options and warrant further evaluation. Indeed, similarly to previous reports in breast cancer and gastric cancer progressing on HER2-targeted therapy, the single most common resistance mechanism in this cohort was loss of *HER2* amplification, which has been correlated with lack of response to later-line HER2-directed therapies.

The limitations of this study include the relatively small sample size and use of a registry control arm. Despite this, the observed efficacy data are consistent with those from the similar phase 2 trials that explored dual-HER2 blockade for mCRC^10,16^. Moreover, the markedly improved ORR observed in TRIUMPH relative to the real-world reference population strongly supports the activity of pertuzumab plus trastuzumab in patients with *RAS* wild-type and *HER2*-amplified mCRC confirmed by tissue or ctDNA analysis.

In conclusion, to our knowledge, this is the first prospective study showing that patients with *HER2-*amplified mCRC identified by ctDNA genotyping benefit from dual-HER2 blockade similarly to patients identified by conventional tissue analysis. Our study also suggests that comprehensive ctDNA genotyping might be able to further refine patient selection for this regimen by identifying primary resistance determinants. Therefore, we recommend the implementation of both ctDNA genotyping and tissue HER2 testing in clinical practice for patients with mCRC to identify candidates for treatment with pertuzumab plus trastuzumab.

## Methods

### Study design and participants

TRIUMPH is a multicenter, open-label, single-arm, phase 2 study in Japan. Eligible patients were aged 20 years or older; had histologically confirmed mCRC; had an Eastern Cooperative Oncology Group performance status score of 0 or 1; had *RAS* wild-type and HER2-positive tumors, defined as IHC 3+ in >10% of tumor cells or FISH-positive (*HER2*/*CEP17* ratio ≥2.0) by tissue testing, or as *HER2*-amplified and *RAS* wild-type by ctDNA analysis; and were refractory or intolerant to fluoropyrimidine, irinotecan and oxaliplatin and to an anti-EGFR antibody.

The study protocol was approved by the institutional review board at each institution (National Cancer Center Hospital East, Aichi Cancer Center Hospital, National Cancer Center Hospital, National Hospital Organization Kyushu Cancer Center, Hokkaido University Hospital, National Hospital Organization Shikoku Cancer Center and National Hospital Organization Osaka National Hospital). The study was conducted in accordance with the protocol, the Ministerial Ordinance on Good Clinical Practice for Drugs and the Declaration of Helsinki. All patients provided written informed consent before enrollment.

### Procedures

Two screening studies, HER2-Screening and GOZILA, were conducted to centrally confirm tissue and ctDNA *HER2* amplification, respectively. HER2-Screening enrolled patients with mCRC. IHC testing was performed using the PATHWAY HER2/*neu* (4B5) Rabbit Monoclonal Primary Antibody (Ventana Medical Systems), and FISH was performed using the PathVysion HER-2 DNA Probe Kit (Abbott Laboratories) at a Clinical Laboratory Improvement Amendments (CLIA)-certified laboratory in Japan. HER2 scoring was conducted according to international consensus diagnostic criteria^1^. GOZILA enrolled patients with metastatic gastrointestinal cancer, including mCRC, who showed disease progression during systemic chemotherapy in HER2-Screening, and performed plasma NGS analysis using Guardant360 at Guardant Health, a CLIA-certified, College of American Pathologists (CAP)-accredited, New York State Department of Health-approved laboratory. Although any patient with mCRC was eligible for both screening studies, those with tumors previously known to harbor *HER2* CN ≥4.0 by tissue NGS testing were preferentially enrolled to efficiently identify HER2-positive tumors.

Patients received intravenous pertuzumab (840 mg loading dose, followed by 420 mg) and trastuzumab (8 mg kg^−1^ loading dose, followed by 6 mg kg^−1^) every 3 weeks until disease progression, death, unacceptable toxicity or discontinuation for any other reasons. Radiographic imaging by computed tomography or magnetic resonance imaging scans was done every 6 weeks during the first 24 weeks of treatment and then every 9 weeks thereafter. Response was assessed per Response Evaluation Criteria in Solid Tumors (RECIST) version 1.1 by local site investigators and by independent central review. All tumor responses were confirmed at least 4 weeks after the initial response. Adverse events were collected throughout treatment and for 30 d thereafter and were graded according to the National Cancer Institute Common Terminology Criteria for Adverse Events version 4.0. Clinical data were collected using Medidata Rave version 2015.1.1 and Medidata Classic Rave version 2018.2.0.

For biomarker analyses, ctDNA genotyping was performed at week 3 and after disease progression.

### Real-world reference in the SCRUM-Japan Registry

Individual patient-level reference data for TRIUMPH were extracted from the SCRUM-Japan Registry as of 29 February 2020 according to pre-defined criteria as described below. The SCRUM-Japan Registry is a longitudinal observational study that generates regulatory-grade real-world data of patients with advanced solid tumors harboring rare alterations identified in the SCRUM-Japan project, including those with mCRC with *HER2* amplification by HER2-Screening or GOZILA. Radiographic imaging was prospectively done every 6–10 weeks according to the study protocol, and response was assessed per RECIST version 1.1 by local site investigators. This study involved 71 core cancer institutions in Japan and was conducted in accordance with the Declaration of Helsinki and the Japanese Ethical Guidelines for Medical and Health Research Involving Human Subjects.

For collection of real-world reference data, individual data of patients who met the following criteria and were not enrolled in TRIUMPH due to any reason, such as being out of the enrollment period or being at a site not participating in TRIUMPH, were extracted:

1. Has *RAS* wild-type
2. Has HER2-positive tumors confirmed by either tissue or ctDNA analysis according to the criteria used in TRIUMPH
3. Is refractory or intolerant to fluoropyrimidine, irinotecan and oxaliplatin or to cetuximab or panitumumab (regardless of treatment history of angiogenesis inhibitors, such as bevacizumab, ramucirumab and aflibercept, as well as trifluridine/tipiracil hydrochloride and regorafenib)

### Outcomes

The primary endpoint was confirmed ORR (defined as the proportion of patients who achieved a complete or partial response confirmed on a follow-up scan ≥4 weeks after the initial response) by investigator assessment. Secondary endpoints were PFS, duration of response (DOR), disease control rate (DCR), OS, confirmed ORR by independent central review and incidences of adverse events. These endpoints, excluding incidences of adverse events, were analyzed for two primary populations: tissue^+^ and ctDNA^+^. Biomarker studies were exploratory.

Patients with *RAS* wild-type and *HER2*-amplified mCRC in the SCRUM-Japan Registry, who were treated with the first salvage-line treatment (defined as the treatment regimen immediately after disease is identified as refractory to or patient is found to be intolerant of fluoropyrimidine, irinotecan and oxaliplatin or to an anti-EGFR antibody), were assessed for real-world ORR. This was defined as the proportion of patients who achieved a complete or partial response in the first salvage-line treatment by investigator assessment.

### Statistical analysis

The planned sample size for each testing group (tissue^+^ and ctDNA^+^) was calculated to be 25 on the basis of a power of 90% to test the null hypothesis of confirmed ORR by investigator assessment of 5%, versus the alternative hypothesis of the ORR of 30%, at a one-sided *α* of 0.025. Five confirmed objective responses were needed to declare the study positive. No adjustment for multiplicity was planned.

Patient characteristics, safety data and anti-tumor activity in TRIUMPH were summarized descriptively. Efficacy endpoints were analyzed for the full analysis set comprising all eligible patients. The 95% CIs of the ORR and DOR were calculated using the Clopper and Pearson method, and PFS, DOR and OS were estimated using the Kaplan–Meier method.

For real-world reference data for TRIUMPH, target sample size was not predetermined. The 95% CI of the real-world ORR was calculated using the Clopper and Pearson method.

Statistical analyses were performed using SAS software (version 9.4). The data cutoff for the analyses was 31 March 2020. TRIUMPH and SCRUM-Japan Registry are registered with the UMIN Clinical Trials Registry—UMIN000027887 and UMIN000028058, respectively.

### Tissue HER2 testing

IHC was performed on 4-μm-thick formalin-fixed, paraffin-embedded (FFPE) sections using the PATHWAY HER2/*neu* (4B5) Rabbit Monoclonal Primary Antibody (not diluted) and BenchMark ULTRA autostainer (Ventana Medical Systems). The IHC score of each sample (0, 1+, 2+ or 3+) was assessed by a certified pathologist (S.F.) using diagnostic criteria according to international consensus diagnostic criteria^17^. IHC scores 0 and 1+ were considered HER2-negative. The proportions of HER2-positive cells were calculated by microscopy, similarly to the method by which pathologists perform routine diagnostics for HER2 in breast and gastric cancers. *HER2* amplification was assessed by FISH using the PathVysion HER-2 DNA Probe Kit and the Duet-3/Setup Station/SOLO2 system (BioView). FISH was performed according to the manufacturer’s instructions. *HER2* amplification was defined as a HER2/CEP17 ratio of ≥2.0.

### ctDNA genotyping in GOZILA

NGS analysis of ctDNA was performed using Guardant360 at Guardant Health^18^. Guardant360 detects single-nucleotide variants (SNVs), indels, fusions and CN alterations in 74 genes with a reportable range of ≥0.04%, ≥0.02%, ≥0.04% and ≥2.12% copies, respectively, as well as microsatellite instability. For GOZILA, 2 × 10 ml of whole blood was collected in Cell-Free DNA BCT (Streck) from enrolled patients and sent to Guardant Health. Then, 5–30 ng of cell-free DNA (cfDNA) isolated from plasma was labeled with non-redundant oligonucleotides (‘molecular barcoding’), enriched using targeted hybridization capture and sequenced on the NextSeq 550 platform (Illumina). Base call files generated by Illumina’s RTA software version 2.12 were demultiplexed using bcl2fastq version 2.19 and processed with a custom pipeline for molecule barcode detection, sequencing adapter trimming and base quality trimming^18^. The processed reads were then aligned to hg19 using the Burrows–Wheeler Aligner-MEM algorithm (arXiv:1303.3997v2). Somatic cfDNA alterations were identified using a proprietary bioinformatics pipeline. ctDNA fraction was measured by the maximum variant allelic fraction (VAF). To estimate the cfDNA clonality for somatic SNVs, indels and fusions, relative clonality was initially defined as alteration VAF/maximum somatic VAF in the sample. Mutations with relative clonality of less than 0.3 were classified as subclonal mutations. The adjusted pCN was calculated as follows: adjusted pCN = (observed pCN − 2 × [1 − T%])/T%, where T% = 2 × maximum VAF/100 (ref. ^19^).

### Tissue NGS analysis

NGS analysis of tumor tissue was performed using Oncomine Comprehensive Assay (OCA) version 1 between February 2015 and March 2017 and OCA version 3 between April 2017 and April 2019 at the Life Technologies Clinical Services Lab, which is a CLIA-certified, CAP-accredited laboratory^20^. These assays covered 143 (OCA version 1) and 161 (OCA version 3) cancer-related genes and detected relevant SNVs, CN variations, gene fusions and indels in one streamlined workflow. In brief, tumor DNA and RNA were isolated from FFPE sections, and DNA/RNA libraries were prepared. Purified libraries were sequenced using Ion Torrent PGM (Thermo Fisher Scientific). Sequence reads were aligned to the hg19 assembly and were called using Ion Reporter Software version 4.4 (for OCA version 1) and version 5.0 (for OCA version 3) to detect alterations. Tissue NGS analysis was performed for 29 patients who enrolled in TRIUMPH, excluding one patient with unavailable tumor tissue.

### Life Sciences Reporting Summary

Further information on research design is available in the Nature Research Reporting Summary linked to this article.

## Online content

Any methods, additional references, Nature Research reporting summaries, source data, extended data, supplementary information, acknowledgements, peer review information; details of author contributions and competing interests; and statements of data and code availability are available at 10.1038/s41591-021-01553-w.

## Supplementary information

Supplementary Information Supplementary Tables 1–4, protocol and statistical analysis plan

Reporting Summary

## Data Availability

The authors declare that all variant data used in the conduct of the analyses are available within the article and Supplementary Table 4. To protect the privacy and confidentiality of patients in this study, clinical data are not made publicly available in a repository or the in the supplementary material of the article, but they will be available upon reasonable request to the corresponding author. Those requests will be reviewed by a study steering committee to verify whether the request is subject to any intellectual property or confidentiality obligations. All data shared will be de-identified.
