# Supplementary material for: Circulating tumor DNA-guided treatment with pertuzumab plus trastuzumab for HER2-amplified metastatic colorectal cancer: a phase 2 trial
Source: Nat Med. 2021 Nov 11;27(11):1899–903. doi: 10.1038/s41591-021-01553-w (PMC8604726; doi:10.1038/s41591-021-01553-w)
Supplement: Supplementary file 1 — Supplementary Tables 1–4, protocol and statistical analysis plan [file 41591_2021_1553_MOESM1_ESM.pdf]

nature portfolio

https://doi.org/10.1038/s41591-021-01553-w

Supplementary information

Circulating tumor DNA-guided treatment with pertuzumab plus trastuzumab for *HER2*-amplified metastatic colorectal cancer: a phase 2 trial

In the format provided by the authors and unedited

Supplementary Table 1. Treatment immediately following refractory or intolerant to a fluoropyrimidine, irinotecan, oxaliplatin, and an anti-EGFR antibody

	n=14
Trifluridine/tipiracil plus bevacizumab	6 (43%)
Trifluridine/tipiracil	3 (21%)
Regorafenib	2 (14%)
FOLFOX plus bevacizumab	1 (7%)
FOLFIRI plus bevacizumab	1 (7%)
S-1	1 (7%)

Data are n (%).

Abbreviations: FOLFOX, combination chemotherapy using fluorouracil, folinic acid, and oxaliplatin; FOLFIRI, combination chemotherapy using fluorouracil, folinic acid, and irinotecan.

Supplementary Table 2. Efficacy endpoints

	Tissue-positive, n=27 (95% confidence interval)	ctDNA-positive, n=25 (95% confidence interval)
Confirmed objective response rate by investigator assessment	30% (14%–50%)	28% (12%–49%)
Confirmed objective response rate by central review	33% (17%–54%)	28% (12%–49%)
Disease control rate by investigator assessment	67% (46%–84%)	60% (39%–79%)
Disease control rate by central review	59% (39%–78%)	60% (39%–79%)
Progression-free survival by investigator assessment	4.0 months (1.4–5.6 months)	3.1 months (1.4–5.6 months)
Progression-free survival by central review	4.2 months (1.5–5.6 months)	4.0 months (1.8–5.6 months)
Duration of response	12.1 months (2.8 months–NR)	8.1 months (2.8 months–NR)
Time to treatment failure	2.8 months (1.4–4.4 months)	2.8 months (1.4–4.4 months)
Overall survival	10.1 months (4.5–16.5 months)	8.8 months (4.3–12.9months)

Supplementary Table 3. Treatment-related adverse events in the overall population (n=30)

	Any grade	Grade 3
Any	24 (80%)	3 (10%)
Infusion-related reaction	14 (47%)	1 (3%)
Diarrhea	11 (37%)	0
Stomatitis	4 (13%)	0
Malaise	3 (10%)	0
Decreased appetite	2 (7%)	0
Nausea	2 (7%)	0
Rash	2 (7%)	0
Abdominal pain	1 (3%)	0
Angina pectoris	1 (3%)	0
Dry eye	1 (3%)	0
Fatigue	1 (3%)	0
Fever	1 (3%)	0
Peripheral edema	1 (3%)	0
Upper abdominal pain	1 (3%)	0
Vomiting	1 (3%)	0
Alkaline phosphatase increased	1 (3%)	0
CPK increased	1 (3%)	0
Dry skin	1 (3%)	0
Dysgeusia	1 (3%)	0
Epistaxis	1 (3%)	0

Exfoliative dermatitis	1 (3%)	0
GGT increased	1 (3%)	1 (3%)
Hypertension	1 (3%)	0
Hyperuricemia	1 (3%)	0
Ejection fraction decreased	1 (3%)	1 (3%)
Neutrophil count decreased	1 (3%)	0
Onycholysis	1 (3%)	0
Paronychia	1 (3%)	0
Platelet count decreased	1 (3%)	0
Rash acneiform	1 (3%)	0
Rash maculopapular	1 (3%)	0
White blood cell decreased	1 (3%)	0

Supplementary Table 4. Baseline genomic alterations and best confirmed response

TRIUMPH ID	Alteration in tissue	Alteration in ctDNA	Best confirmed response
1	*APC* mutation<br>*ERBB2* amplification	*APC* mutation<br>*ERBB2* amplification<br>*PDGFRA* mutation	Stable disease
2	*ERBB2* amplification<br>*TP53* mutation	Not analyzed	Stable disease
3	*APC* mutation<br>*ERBB2* amplification<br>*TP53* mutation	*APC* mutation<br>*ERBB2* amplification<br>*TP53* mutation	Stable disease
4	*CDK6* amplification<br>*ERBB2* amplification	*ERBB2* amplification<br>*TP53* mutation	Stable disease
5	*ERBB2* amplification<br>*TP53* mutation	*APC* mutation<br>*CDK6* amplification<br>*ERBB2* amplification<br>*MET* mutation<br>*TP53* mutation	Partial response
6	*ERBB2* amplification<br>*FGFR1* amplification<br>*TP53* mutation	*APC* mutation<br>*ERBB2* amplification<br>*TP53* mutation	Partial response
7	*ERBB2* amplification<br>*TP53* mutation	*ERBB2* amplification<br>*TP53* mutation	Progressive disease

8	*ERBB2* mutation<br>*ERBB2* amplification<br>*TP53* mutation	*APC* mutation<br>*ERBB2* mutation<br>*ERBB2* amplification<br>*TP53* mutation	Progressive disease
9	*ERBB2* amplification	*ERBB2* amplification<br>*TP53* mutation	Stable disease
10	*ERBB2* amplification	*APC* mutation<br>*ERBB2* mutation	Partial response
11	*CCND2* amplification<br>*ERBB2* amplification<br>*TP53* mutation	*APC* mutation<br>*CCND2* amplification<br>*CDK4* amplification<br>*CDK6* amplification<br>*ERBB2* amplification<br>*MET* amplification<br>*PIK3CA* mutation<br>*TP53* mutation	Progressive disease
12	*TP53* mutation	*APC* mutation<br>*EGFR* amplification<br>*ERBB2* amplification<br>*KIT* mutation<br>*MET* mutation<br>*TP53* mutation	Progressive disease
13	*ERBB2* amplification<br>*TP53* mutation	*APC* mutation<br>*ERBB2* amplification<br>*TP53* mutation	Complete response

14	*TP53* mutation	*APC* mutation<br>*BRAF* amplification<br>*CCNE1* amplification<br>*EGFR* amplification<br>*FGFR1* amplification<br>*KRAS* mutation<br>*TP53* mutation	Progressive disease
15	*BRAF* mutation	*BRAF* mutation<br>*CCNE1* amplification<br>*ERBB2* amplification<br>*TP53* mutation	Progressive disease
16	*ERBB2* amplification<br>*TP53* mutation	*APC* mutation	Partial response
17	*ERBB2* amplification<br>*TP53* mutation	*ERBB2* amplification<br>*TP53* mutation	Partial response
18	*ERBB2* amplification<br>*TP53* mutation	*APC* mutation<br>*EGFR* amplification<br>*ERBB2* amplification<br>*PIK3CA* mutation<br>*TP53* mutation	Progressive disease
19	*ERBB2* amplification<br>*TP53* mutation	*TP53* mutation	Stable disease
20	*ERBB2* amplification<br>*ERBB2* fusion	*APC* mutation<br>*ERBB2* amplification<br>*TP53* mutation	Stable disease

21	Not analyzed	*APC* mutation<br>*CDK6* amplification<br>*ERBB2* amplification<br>*MET* amplification<br>*TP53* mutation	Stable disease
22	*ERBB2* mutation<br>*ERBB2* amplification<br>*TP53* mutation	*APC* mutation<br>*ERBB2* mutation<br>*ERBB2* amplification<br>*TP53* mutation	Stable disease
23	*ERBB2* amplification	*APC* mutation<br>*ERBB2* amplification<br>*FGFR1* amplification<br>*TP53* mutation	Progressive disease
24	*ERBB2* amplification	*APC* mutation<br>*CDKN2A* mutation<br>*ERBB2* mutation<br>*TP53* mutation	Stable disease
25	*ERBB2* amplification<br>*TP53* mutation	*CCNE1* amplification<br>*EGFR* amplification<br>*ERBB2* amplification	Progressive disease
26	*ERBB2* amplification	*APC* mutation<br>*BRAF* amplification<br>*CDK4* amplification<br>*ERBB2* mutation<br>*ERBB2* amplification	Progressive disease

27	*CCND1* amplification<br>*ERBB2* amplification<br>*FLT3* amplification	*APC* mutation<br>*CCND1* amplification<br>*ERBB2* amplification	Partial response
28	*ERBB2* amplification<br>*TP53* mutation	*APC* mutation<br>*ERBB2* amplification<br>*TP53* mutation	Stable disease
29	Not detected	*APC* mutation<br>*EGFR* amplification<br>*ERBB2* amplification	Progressive disease
30	*CTNNB1* mutation<br>*ERBB2* amplification	*APC* mutation<br>*CTNNB1* mutation<br>*ERBB2* amplification<br>*TP53* mutation	Partial response

Multicenter Phase II study in patients with HER2-positive metastatic colorectal cancer

Investigator-initiated clinical study protocol

TRIUMPH study

TRastuzumab comb**I**ned with pertuz**UM**ab for **P**atients with **H**er2-positive mCRC

Protocol No.: EPOC1602

Coordinating investigator (representative): Wataru Okamoto (until March 31, 2019)

Chief of BB/TR Support Section, Clinical Research Support Office,
National Cancer Center Hospital East
6-5-1 Kashiwanoha, Kashiwa, Chiba, 277-8577, Japan
TEL: 04-7133-1111 (extension 91551)
E-mail: wokamoto@east.ncc.go.jp

Coordinating investigators: Yoshiaki Nakamura, Akihiro Sato, and Takayuki Yoshino (representative from April 1, 2019)

Department of Gastrointestinal Oncology and Clinical Research Support Office,
National Cancer Center Hospital East,
6-5-1 Kashiwanoha, Kashiwa, Chiba, 277-8577, Japan
TEL: 04-7133-1111 (extension: Nakamura 91397, Sato 91247, Yoshino 93011)
E-mail: triumph_core@east.ncc.go.jp

Confidentiality notice

This protocol contains confidential information and is provided for the coordinating committee, investigators, subinvestigators, study collaborators (including contract research organizations such as site management organizations), study sites, institutional review boards, and the response and safety evaluation committee.

Except when you explain details in this study to patients, you must not disclose this protocol to any third party or use it for the purposes other than this study without written approval of the coordinating committee and the pharmaceutical company providing study drugs.

Protocol No.: EPOC1602

Version No.: Version 8.0, prepared on November 10, 2020

History of revision:

June 28, 2017: Preparation of the investigator-initiated clinical study protocol ver 1.0

July 25, 2017: Preparation of the investigator-initiated clinical study protocol ver 1.01

July 26, 2017: Preparation of the investigator-initiated clinical study protocol ver 1.02

September 15, 2017: Preparation of the investigator-initiated clinical study protocol ver 2.0

December 22, 2017: Preparation of the investigator-initiated clinical study protocol ver 3.0

May 1, 2018: Preparation of the investigator-initiated clinical study protocol ver 4.0

August 15, 2018: Preparation of the investigator-initiated clinical study protocol ver 5.0

November 2, 2018: Preparation of the investigator-initiated clinical study protocol ver 6.0

January 18, 2019: Preparation of the investigator-initiated clinical study protocol ver 7.0

November 10, 2020: Preparation of the investigator-initiated clinical study protocol ver 8.0

略語一覧

5-FU	Fluorouracil
AE	Adverse event
ARDS	Acute respiratory distress syndrome
BSC	Best supportive care
CISH	Chromogenic in situ hybridization
CTCAE	Common terminology criteria for adverse events
CRF	Case report form
CT	Computed tomography
DCR	Disease control rate
DoR	Duration of response
ECD	Extracellular domain
ECI	Events of clinical interest
ECOG	Eastern cooperative oncology group
EGFR	Epidermal growth factor receptor
FISH	Fluorescence in situ hybridization
FAS	Full analysis set
GCP	Good clinical practice
G-CSF	Granulocyte colony stimulating factor
HBs	Hepatitis B surface
HER	Human epidermal growth factor receptor
HIV	Human immunodeficiency virus
HR	Hazard ratio
IAR	Infusion-associated reaction
Ig	Immunoglobulin
IRB	Institutional Review Board
IHC	Immunohistochemistry
ISH	In situ hybridization
l-LV	Levofolinate calcium
LVEF	Left ventricular ejection fraction
LVSD	Left ventricular systolic dysfunction
MRI	Magnetic resonance imaging
MUGA	Multigated acquisition scan
NGS	Next-generation sequencing
ORR	Objective response rate

OS	Overall survival
PDX	Patient-derived xenograft
PFS	Progression-free survival
PS	Performance Status
RAS	Rat sarcoma
RECIST	Response evaluation criteria in solid tumors
SISH	Silver-enhanced in situ hybridization
SP	Safety population
TTF	Time to treatment failure
VEGF(R)	Vascular endothelial growth factor (receptor)

0 Summary

Phase of development	Phase II
Objective	The objective of this study is to evaluate the efficacy and safety of the concurrent therapy of trastuzumab and pertuzumab in patients with human epidermal growth factor receptor (HER) 2-positive metastatic colorectal cancer who are refractory or intolerant to standard chemotherapy.<br>**Primary endpoint:**<br>Confirmed objective response rate（ORR）assessed by investigator review<br>Analysis was performed in the following two analysis sets.<br>• Patients with HER2 positive tumor confirmed by tissue analysis<br>• Patients with HER2 positive and *RAS* wild-type tumor confirmed by analysis of blood sample<br>**Secondary endpoints:**<br>The following items were evaluated for each of “patients with HER2 positive tumor confirmed by tissue analysis” and “patients with HER2 positive and *RAS* wild-type tumor confirmed by analysis of blood sample.”<br>• Progression-free survival (PFS)<br>• Duration of response (DoR)<br>• Time to treatment failure (TTF)<br>• Disease control rate (DCR)<br>• Overall survival (OS)<br>• Confirmed ORR assessed by central review*<br>• Percentage change in sum of tumor diameters<br>• Incidences of adverse events<br>• ORR and PFS in the pretreatment regimens containing an anti-EGFR antibody drug (cetuximab or panitumumab)<br><br>*Central assessment of objective tumor response may not be performed for reasons such as cost.<br><br>The endpoints shown above will be analyzed by comparing the data in this study with the control data from the natural history follow-up group and from patients with HER2-positive metastatic colorectal cancer in the SCRUM-Japan registry.

	Details of these analyzed items will be analyzed and compared in accordance with the statistical analysis plan specified separately.
Study design	This is an open-label, single-arm, multicenter, Phase II study to evaluate the efficacy and safety of the concurrent therapy of trastuzumab and pertuzumab in patients with HER2-positive metastatic colorectal cancer who are refractory or intolerant to standard chemotherapy.
Target population	Patients who meet both the eligibility criteria A and B and do not meet any of the exclusion criteria will be enrolled as target patients for study treatment in the study. Patients who meet the eligibility criteria A, and do not meet the eligibility criteria B or meet any of the exclusion criteria, will be enrolled in the natural history follow-up group and will be investigated every 3 months for information on antitumor treatment and survival but not for adverse events.<br><br>**Eligibility criteria A:**<br>1. A patient aged 20 years or above<br>2. A patient with metastatic colorectal cancer that is histologically diagnosed as adenocarcinoma<br>3. A patient who has tumor tissue with wild-type *KRAS* codons 12 and 13. If other codons than *KRAS* codons 12 and 13 (*KRAS* codons 59, 61, 117, and 146 and *NRAS* codons 12, 13, 59, 61, 117, and 146) are also analyzed, the statuses for all these *RAS* codons must be wild type.<br>4. A patient with cancer that meets either the following criteria I or II<br>I .A patient with cancer that meets either of the following based on analysis of tumor tissue in central pathological assessment* using the HER2 IHC test and HER2 FISH test<br>i. IHC3+<br>ii. FISH positive (*HER2*/*CEP17* ratio ≥ 2.0)<br>II .A patient who has *HER2* amplification (++ or +++) (HER2 positive) and wild-type *RAS*[†] based on analysis of a blood sample in central assessment* using liquid biopsy<br>[†]Wild-type *RAS* is defined as “Relative clonality[††] is equal to or less than 30% in each *KRAS* codon 12, 13, 59, 61, 117, and 146, and *NRAS* codon 12, 13, 59, 61, 117, and 146.”

†† relative clonality (%) = % cfDNA of a certain mutation / the highest % cfDNA x 100

*The HER2 test of tumor tissue, the liquid biopsy using blood samples and central assessment of the results will be performed in clinical research conducted separately.

5. A patient who is refractory or intolerant to fluorinated pyrimidine antimetabolites, oxaliplatin, irinotecan, cetuximab, or panitumumab (with or without a treatment history with angiogenic inhibitors such as bevacizumab, ramucirumab and aflibercept, trifluridine-tipiracil combination, and regorafenib)
   *In patients with recurrence during adjuvant chemotherapy or within 6 months after the final dose, adjuvant chemotherapy is counted as treatment history.
6. A patient with an Eastern Cooperative Oncology Group (ECOG) Performance Status of 0 or 1
7. A patient with an expected survival of at least 12 weeks
8. A patient who has given written consent

Eligibility criteria B:

1. A patient who has never been treated with HER2-targeted drugs (including pan-HER-targeted drugs)
2. A patient with a measurable lesion based on the Response Evaluation Criteria in Solid Tumors (RECIST) Version 1.1
3. A patient with the organ functions that meet the following:
   - Neutrophil count ≥ 1,000/mm^3
   - Platelet count ≥ 75,000/mm^3
   - Hemoglobin ≥ 8.0 g/dL
   - Serum creatinine ≤ 2.0 mg/dL, or calculated (Cockcroft-Gault equation*) or measured creatinine clearance ≥ 50 mL/min
   - Total bilirubin ≤ 2.0 mg/dL (If a patient with Gilbert's syndrome has a value of > 2.0 mg/dL, the patient can be enrolled after the coordinating committee agrees.)
   - ALT and AST ≤ 100 U/L (patients with liver metastasis: ≤ 200 U/L)

   *Cockcroft-Gault equation: Creatinine clearance value = (140 − age) × body weight (kg)/(72 × serum creatinine value) (*for women, multiplying the obtained value by 0.85 gives the creatinine clearance)
4. A patient with a left ventricular ejection fraction (LVEF) of 50% or above measured by echocardiography, multigated acquisition scan (MUGA), or

	magnetic resonance imaging (MRI)<br>5. A patient with no symptomatic brain metastasis or meningeal dissemination<br>6. A patient who is not breast-feeding<br>7. A patient who is willing to use appropriate contraception* throughout study treatment and for at least 7 months after the last dose of pertuzumab or trastuzumab<br>*The following methods of contraception are used.<br>- Abstaining from sexual intercourse<br>- Using a combination hormone contraception drug<br>- For women, having only one partner, who is a man who has undergone vasectomy<br>- For men, undergoing vasectomy<br>8. A patient without a history of cardiovascular disease shown below within 6 months before the start of study treatment<br>- Acute myocardial infarction<br>- Angina pectoris that requires intervention<br>- Symptomatic congestive cardiac failure<br>9. A patient without inadequately controlled hypertension (systolic pressure ≥ 180 mmHg or diastolic pressure ≥ 100 mmHg)<br>10. A patient with no history of inadequately controlled arrhythmia (heart rate at rest > 100 beats per minute), ventricular arrhythmia, or severe atrioventricular block (Mobitz type II second-degree atrioventricular block or third-degree atrioventricular block)<br>- A patient who has chronic atrial arrhythmia with controlled pulse rate is eligible if the patient does not have any other cardiovascular abnormality.<br>11. A patient with a negative result on the human immunodeficiency virus (HIV) antibody test<br>12. A patient with a negative result on the hepatitis B surface (HBs) antigen test<br>13. A patient who is not receiving long-term treatment with systemic steroids at a prednisolone equivalent dose > 10 mg/day<br>14. A patient with no history of other malignant tumors within 3 years before the start of study treatment. However, if a patient has a history of the lesion equivalent to carcinoma in situ or intramucosal carcinoma that is judged cured after local treatment, non-metastatic prostate cancer that does not

	require systemic treatment, or other solid cancers that do not require treatment or to which study treatment is not considered disadvantageous.<br>15. A patient who has not undergone another antitumor treatment concurrently<br>- A patient who has not undergone pretreatment (chemotherapy, molecular targeted therapy, antibody therapy, or radiotherapy) within 2 weeks before the start of study treatment<br>- Pretreatment with bisphosphonates and denosumab is allowed.<br>16. A patient who has recovered from toxicity due to pretreatment (to Grade 1 or below or to baseline). However, patients with abnormal blood test levels within the range meeting the eligibility criterion B.3., patients with Grade 2 peripheral sensory neuropathy, Grade 2 alopecia, Grade 2 skin hyperpigmentation, controllable hypertension, and hypocalcemia/hypomagnesemia are eligible.<br>17. A patient who has not undergone major surgery within 4 weeks before the start of study treatment. However, patients who have undergone colostomy earlier than 2 weeks before the start of study treatment are eligible.<br>18. A patient with no other serious, clinically significant abnormalities that are acute or chronic<br>19. A patient who does not want study treatment<br><br>**Exclusion criteria**<br>1. A patient who had a history of bone marrow transplantation or organ transplantation.<br>2. A patient who is a woman of childbearing potential and has a positive result on a serum pregnancy test.<br>3. A patient with active hemorrhagic lesions.<br>4. A patient whom the investigator judges to be unsuitable for enrollment
Sample size and statistical analysis	For each of the analysis sets “patients with HER2 positive tumor confirmed by tissue analysis” and “patients with HER2 positive tumor confirmed by analysis of blood sample,” the threshold and expected value of the objective response rate for study treatment, which is the primary endpoint, were set at 5% and 30%, respectively, with a one-sided significance level of 2.5% and a power of 80%, and the target sample size for each analysis set was set at 18 (on the basis of a precise method based on the binomial distribution). When at least 4 of the 18 subjects have an objective response, the result will be considered statistically significant. In each analysis set, if subjects are enrolled at a good

	rate, whether the power can be raised to 90% (whether the target sample size can be increased to 25) will be evaluated during the study.
Method of treatment	Combination therapy of trastuzumab + pertuzumab<br>• Trastuzumab: 8 mg/kg (first dose), 6 mg/kg (second and subsequent doses), every 3 weeks<br>• Pertuzumab: 840 mg (first dose), 420 mg (second and subsequent doses), every 3 weeks<br>Both drugs will be intravenously infused.
Safety evaluation	Safety will be evaluated with the Common Terminology Criteria for Adverse Events (CTCAE) Version 4.0 - JCOG (corresponding to CTCAE v4.03/MedDRA Version 12.0).
Efficacy evaluation	Objective tumor response will be assessed in accordance with the RECIST version 1.1.<br>Image evaluation: Computed tomography (CT) or MRI (CT is preferable for the evaluation of antitumor response.)
Screening research	HER2-positive patients will be identified in clinical research conducted separately.
Biomarker evaluation	Biomarkers will be evaluated as a research accompanying this study, and the plan will be prepared separately.
Interim analysis	It will not be performed.
Planned study period	Planned enrollment period: December 2017 to December 2019 (for 25 months)<br>Planned observation period: Until March 2022<br>Entire study period (including procedures for the completion of the study): Until March 2023
The number of study sites	7 sites
Representative coordinating investigator	Wataru Okamoto (until March 31, 2019), Takayuki Yoshino (from April 1, 2019), National Cancer Center Hospital East
Planned study sites	Hokkaido University Hospital<br>National Cancer Center Hospital East<br>National Cancer Center Hospital<br>Aichi Cancer Center<br>Osaka National Hospital<br>Shikoku Cancer Center

	Kyushu Cancer Center
Implementation structure of the study	The conduct of this study will be funded by the research grant for "the investigator-initiated study in patients with HER2-positive unresectable or recurrent colorectal cancer utilizing the Cancer Genome Screening Project for Individualized Medicine in Japan (SCRUM-Japan) patient registry" (representative: Wataru Okamoto, Clinical Research Support Office [until March 31, 2019], Takayuki Yoshino, Department of Gastrointestinal Oncology [from April 1, 2019] National Cancer Center Hospital East) in the Project Promoting Clinical Trials for Development of New Drugs and Medical Devices in FY 2016 by the Japan Agency for Medical Research and Development until March 31, 2021, and Chugai Pharmaceutical Co., Ltd. from April 1, 2021. Historical data will be provided to the SCRUM-Japan disease registry for free. Study drugs (trastuzumab and pertuzumab) will be provided by Chugai P harmaceutical Co., Ltd.
Expected results from the study	When the efficacy and safety of the concurrent therapy of trastuzumab and pertuzumab are demonstrated in patients with HER2-positive unresectable or recurrent colorectal cancer, an effective treatment against unresectable or recurrent colorectal cancer may be developed, and a new disease class of HER2-positive unresectable or recurrent colorectal cancer can be established to provide evidence for precision medicine.

TABLE OF CONTENTS

0 Summary 5

1 Objectives 18

2 Backgrounds and rationales for the study plan 19

2.1 Target patients 19

2.1.1 Epidemiology of colorectal cancer 19

2.2 Standard treatment against colorectal cancer 19

2.3 Roll of HER2 signals in colorectal cancer 20

2.3.1 Pathological significance of HER2 overexpression 20

2.3.2 Frequency of HER2-positive cases in colorectal cancer 20

2.3.3 Relationship between *HER2* amplification and resistance to anti-EGFR antibody drugs 22

2.3.4 Development of HER2-targeted treatment against HER2-positive unresectable or recurrent colorectal cancer（Table 2.b） 23

2.3.5 Concurrent therapy of trastuzumab and pertuzumab against HER2-positive unresectable or recurrent colorectal cancer 24

2.4 Rationales for the treatment plan 27

2.4.1 Treatment regimen in this study 27

2.4.2 Rationales for the dosage and treatment schedule 28

2.5 Posttreatment 28

2.6 Study design 28

2.6.1 Target patients 29

2.6.2 Endpoints 33

2.6.3 Rationales for endpoints 34

2.6.4 Clinical hypothesis and rationales for the sample size 35

2.6.5 Expected number of enrolled patients and the periods of enrollment and follow-up 36

2.6.6 Control group 37

2.7 Summary of expected benefits and disadvantages from participation in the study 38

2.7.1 Expected benefits 38

2.7.2 Expected risks and disadvantages 38

2.8 Reasons why this is conducted as an investigator-initiated study 38

2.9 Accompanying research 39

3 Criteria and definitions used in this study 40
3.1 Performance Status (Eastern Cooperative Oncology Group [ECOG] classification) 40
3.2 Criteria for the assessment of adverse events 40
3.3 Criteria for the assessment of response rates 40
3.4 HER2-positive criteria 40
4 Inclusion and exclusion criteria 41
4.1 Inclusion criteria of this study 41
4.2 Follow-up of natural history 44
5 Enrollment and allocation 45
5.1 Enrollment procedure 45
5.2 Notes for enrollment 47
5.3 Operations related to enrollment in two analysis sets 47
6 Treatment plan and criteria for change in treatment 49
6.1 Study treatment 49
6.1.1 Pertuzumab 49
6.1.2 Trastuzumab 50
6.2 Discontinuation criteria for study treatment 51
6.2.1 Discontinuation criteria for study treatment 51
6.2.2 Data Collection and Follow-up of Patients after Discontinuation of the protocol treatment 52
6.3 Criteria for discontinuation of the protocol treatment 52
6.3.1 Delay or discontinuation of treatment due to cardiac toxicity 52
6.3.2 Infusion-associated reactions 54
6.4 Concomitant therapies 55
6.4.1 Permitted concomitant therapies 55
6.4.2 Prohibited concomitant therapies 56
7 Information on study drugs 57
7.1 Pharmaceutical information on trastuzumab 57
7.2 Pharmaceutical information on pertuzumab 57
7.3 Control of study drugs 57
7.4 Expected adverse events 57
8 Endpoints, laboratory test, and evaluation schedule 59
8.1 Study calendar 60
8.2 Collection, tests, and observations during the study and their procedures 62

8.2.1 Informed consent 62
8.2.2 Patient characteristics 62
8.2.3 HER2 status 64
8.2.4 Measurement of body height, weight, and vital signs 64
8.2.5 ECOG Performance Status 64
8.2.6 Clinical findings (including adverse events) and concomitant drugs/therapies 64
8.2.7 Resting 12-lead electrocardiogram 64
8.2.8 LVEF 64
8.2.9 Status of study treatment 64
8.3 Laboratory test 64
8.3.1 Hematology and biochemistry 65
8.3.2 Tumor markers 65
8.3.3 Pregnancy test 65
8.3.4 Infection test 65
8.3.5 Biopsy and blood sampling for biomarkers 65
8.4 Imaging test 66
8.5 Follow-up 66
9 Data collection 67
9.1 Handling and retention of CRF data 67
9.2 Identification of source documents 67
10 Reporting of adverse events 68
10.1 General toxicity related to trastuzumab and pertuzumab 68
10.1.1 Risks of hypersensitivity reactions (including anaphylaxis) associated with infusion 68
10.1.2 Risks of decreased left ventricular ejection fraction and symptomatic cardiac failure 68
10.2 General toxicity related to pertuzumab 69
10.2.1 Risk of toxicity related to epithelial growth factor receptor (HER1) 69
10.3 Evaluation of Adverse Events 69
10.3.1 Definition of Adverse Events 69
10.3.2 Method for Recording AEs 70
10.3.3 Items to Be Recorded for AEs 70
10.3.4 Assessment of the Causal Relationship 70
10.3.5 Follow-up of the Clinical Course at the Onset of AEs 72
10.3.6 AE Evaluation Period 72
10.4 Reporting of Serious Adverse Events (SAEs) 72

10.4.1 Definition of SAE 72
10.4.2 Events of clinical interest (ECI) 73
10.4.3 Reporting procedure of the Investigator to the head of the study site and the coordinating committee 73
10.4.4 Reporting obligation of the coordinating committee and reporting procedures 74
10.4.5 Duties of the efficacy and safety evaluation committee 75
10.5 Collection of safety information 75
10.6 Follow-up of pregnancy 75
10.6.1 Pregnancy in female subjects 75
10.6.2 Pregnancy in female partners of male subjects 76
11 Assessment of response and definitions of endpoints 77
11.1 Assessment of response 77
11.2 Definitions of endpoints 77
11.2.1 Confirmed objective response rate (ORR) assessed by investigator review 77
11.2.2 Confirmed Objective Response rate (ORR) assessed by central review 77
11.2.3 Progression free survival (PFS) 77
11.2.4 Duration of response (DoR) 78
11.2.5 Time to treatment failure (TTF) 78
11.2.6 Disease control rate (DCR) 79
11.2.7 Overall survival (OS) 79
11.2.8 Percentage change in sum of tumor diameters 80
11.2.9 Incidence of AEs 80
11.2.10 ORR in regimens including anti-EGFR antibody drugs (cetuximab or panitumumab) in pretreatment 80
12 Statistical matters 81
12.1 Handling of the patients 81
12.2 Definitions of analysis populations 81
12.3 Positioning of analysis and analysis method 82
12.4 Efficacy analysis 83
12.4.1 Primary endpoint 83
12.4.2 Secondary endpoints 83
12.5 Safety analysis 84
12.6 Handling of data 84
12.6.1 Handling of missing values and outliers 84
12.6.2 Handling of additional analysis 85
12.7 Target sample size 85

12.7.1 Rationales for determination 85
12.8 Interim analysis 85
12.9 Primary analysis 85
12.10 Analysis performed to prepare the clinical study report 85
12.11 Final analysis 86
12.12 Remarks 86
12.12.1 Comparison with the comparative control group 86
12.12.2 Integrated analysis with external data 87
12.12.3 Analysis sets 88
12.12.4 Other exploratory analysis 88
13 Ethical matters 90
13.1 Policies, laws and regulations with which the study complies 90
13.2 Informed consent 90
13.2.1 Explanation to the patients 90
13.2.2 Informed consent 91
13.3 Protection of personal information and patient identification 91
13.3.1 Purposes of using personal information, items to be used and methods of use 91
13.3.2 Secondary use of data 92
13.3.3 Safety management responsibility system 92
13.3.4 Handling of disclosure of the patients' information 92
13.4 Approval of the institutional review board (IRB) 93
13.4.1 Approval at the start of the study 93
13.4.2 Approval of the IRB for the appropriateness of continuing the study 93
13.4.3 Changes in the contents of the protocol 93
13.4.4 Categories of changes in the contents of the protocol 93
13.4.5 Approval of the IRB at the time of protocol revision 94
13.5 Management of conflict of interest (COI) 94
13.6 Compensation 94
14 Monitoring and audits 95
14.1 Monitoring 95
14.2 Protocol deviations and violation 95
14.3 Audits 95
14.4 Direct access 95
15 Special note 97
15.1 Central assessment of the objective tumor response 97

15.2 Retention of records **97**
15.2.1 Sponsor-investigator 97
15.2.2 Study sites 97
15.3 Completion of the study **97**
15.4 Discontinuation at the study site **98**
15.5 Interruption of the study and discontinuation of the entire study **98**
15.5.1 Interruption of the study 98
15.5.2 Discontinuation of the entire study 98
16 Study organization **99**
16.1 Study implementation structure **99**
16.2 Funding source of the study **99**
17 Attribution of study results and publication of study results **100**
18 REFERENCES **101**
Appendix A ECOG Performance Status **106**
Appendix B Assessment according to RECIST ver. 1.1 .... **107**

1 Objectives

The objective of this study is to evaluate the efficacy and safety of the concurrent therapy of trastuzumab and pertuzumab in patients with human epidermal growth factor receptor (HER) 2-positive unresectable or recurrent colorectal cancer who are refractory or intolerant to standard chemotherapy.

The endpoints of this study are as follows.

Primary endpoint:

Confirmed objective response rate（ORR）assessed by investigator review

Analysis was performed in the following two analysis sets.

- Patients with HER2 positive tumor confirmed by tissue analysis
- Patients with HER2 positive and *RAS* wild-type tumor confirmed by analysis of blood sample

Secondary endpoints:

The following items were evaluated for each of “patients with HER2 positive tumor confirmed by tissue analysis” and “patients with HER2 positive and *RAS* wild-type tumor confirmed by analysis of blood sample.”

- Progression-free survival (PFS)
- Duration of response (DoR)
- Time to treatment failure (TTF)
- Disease control rate (DCR)
- Overall survival (OS)
- Confirmed ORR assessed by central review*
- Percentage change in sum of tumor diameters
- Incidences of adverse events
- ORR and PFS in the pretreatment regimens containing an anti-EGFR antibody drug (cetuximab or panitumumab)

*Central assessment of objective tumor response may not be performed for reasons such as cost.

The endpoints shown above will be analyzed by comparing the data in this study with the control data from the natural history follow-up group and from patients with HER2-positive metastatic colorectal cancer in the SCRUM-Japan registry. Details of these analyzed items will be analyzed and compared in accordance with the statistical analysis plan specified separately.

2 Backgrounds and rationales for the study plan

2.1 Target patients

2.1.1 Epidemiology of colorectal cancer

Colorectal cancer is the cancer with the world's third largest number of patients and the world's fourth largest number of deaths[1]. Also in Japan, the crude prevalence rate and the crude death rate for colorectal cancer tend to be increased, and the deaths due to colorectal cancer account for 5.2% (in men) and 11.7% (in women) of all cancer deaths[2].

2.2 Standard treatment against colorectal cancer

According to the guidelines for treatment of colorectal cancer (2016), standard treatment against colorectal cancer depends on cancer staging[3]. Cancer at Stage 0 (that remains in the mucosa) is often resected endoscopically. Cancer at Stages I–III is resected surgically. For cancer at Stage III with metastases to lymph nodes and cancer at Stage II with a high risk for recurrence, adjuvant chemotherapy is given. For unresectable or recurrent cancer, systemic chemotherapy is provided. If metastases to the liver and lung are considered resectable, they may be resected surgically.

As the first-line treatment against unresectable or recurrent colorectal cancer, cytotoxic antineoplastic drugs such as fluorinated pyrimidine antimetabolites (fluorouracil [5-FU] + levofolinate calcium [*l*-LV], capecitabine, S-1, tegafur-uracil + leucovorin [LV]), oxaliplatin, and irinotecan are mainly combined. The combination of fluorinated pyrimidine antimetabolites and oxaliplatin includes FOLFOX, CapeOX, and SOX, and the combination of fluorinated pyrimidine antimetabolites and irinotecan includes FOLFIRI and IRIS. A three-drug combination called FOLFOXIRI (5-FU + *l*-LV in combination with oxaliplatin and irinotecan) is also an option. The above regimens with cytotoxic antineoplastic drugs in combination with an anti-VEGF antibody drug, bevacizumab, and anti-EGFR antibody drug, cetuximab or panitumumab (for wild-type *RAS* only) are the current standard of the first-line treatment.

In principle, recommended second-line treatment is irinotecan-based regimens in patients treated with oxaliplatin as the first-line treatment and oxaliplatin-based regimens in patients treated with irinotecan as the first-line treatment. For the combination of molecular target drugs in second-line treatment, an option in patients with wild-type *RAS* treated with an anti-EGFR antibody drug as the first-line treatment is to switch it to bevacizumab or an anti-VEGFR antibody drug, ramucirumab. Options in patients treated with bevacizumab as the first-line treatment are to switch it to an anti-EGFR antibody drug or ramucirumab and to continue treatment with bevacizumab. For patients with *RAS* mutation, an option is the concurrent use of bevacizumab or ramucirumab.

As the third-line treatment in patients with wild-type *RAS* who have never used anti-EGFR antibody drugs in first- or second-line treatment, irinotecan + an anti-EGFR antibody drug or an anti-EGFR antibody drug alone is recommended. As salvage-line treatment after cytotoxic antineoplastic drugs, anti-VEGF/VEGFR antibody drugs, and anti-EGFR antibody drugs are used, a small-molecule compound, regorafenib, and trifluridine-tipiracil combination have shown an effect of prolonging survival compared with the best supportive care (BSC)[4,5].

2.3 Roll of HER2 signals in colorectal cancer

2.3.1 Pathological significance of HER2 overexpression

Human epithelial growth factor receptor 2 (HER2) is a tyrosine kinase receptor that has been found to undergo gene amplification or be overexpressed in various cancers including breast cancer and gastric cancer. From the results of non-clinical and clinical studies, it is inferred that the overexpression of HER2 is directly involved in the pathogenesis of human tumors and their poor prognosis[6]. If a mutant *HER2* gene is transfected into mouse fibroblasts (NIH3T3), the cells transform into malignant cells, and it results in the formation of tumors in nude mice[7, 8]. In addition, transgenic mice overexpressing *neu* genes (the rodent homolog of human *HER2* genes) develop breast cancer[9]. Antibodies specific to the extracellular domain (ECD) of HER2 suppress the growth of tumors with HER2 overexpression[10-13]. These data suggest that driver genetic alterations in HER2 have a role in the malignant transformation of cells and promotion of tumor formation. Given this background, therapies that antagonize abnormal function due to HER2 overexpression are being developed in order to improve the prognosis in cancer patients with HER2 overexpression.

2.3.2 Frequency of HER2-positive cases in colorectal cancer

Since the 1990s, it has been reported that HER2 proteins are overexpressed in some colorectal cancer[14]. Recently, some reports have shown the results of analyses on HER2 overexpression and *HER2* amplification in colorectal cancer in larger cohorts (Table 2.a).

Table 2.a Frequencies of HER2 overexpression and *HER2* amplification in colorectal cancer

Research	HER2 test	Number of cases	Number of HER2-positive cases (%)
TCGA[15]	SNP array	257 (Stage I-IV)	7 (2.7%)
Heppner BI, et al. [16]	IHC, CISH	1645 (Stage I-IV)	26 (1.6%)

Valtorta E, et al. [17]	IHC, FISH	1086 (unresectable or recurrent)	56 (5.2%)*<br>**KRAS* エクソン 2 野生型に限る
Richman SD, et al. [18]	IHC, FISH	1914 (Stage II, III)<br>1342 (unresectable or recurrent)	25 (1.3%)<br>29 (2.2%)
Kato T, et al. [19]	NGS (SCRUM-Japan GI-SCREEN)	266 (unresectable or recurrent)	11 (4.1%)

The Cancer Genome Atlas (TCGA) network reported the results of comprehensive molecular biological analysis in colorectal cancer in 2012[15]. In the research, a change in the gene copy number was analyzed in 257 samples by Affymetrix SNP 6.0 Array, and *HER2* amplification was observed in 7 samples (2.7%). The cases of colorectal cancer were classified into the subgroup of “hypermutated” or “non-hypermutated” depending on the frequency of gene mutation, and all cases of *HER2* amplification were in the “non-hypermutated” subgroup.

Heppner BI, et al. evaluated the HER2 status in 1645 surgical samples of colorectal cancer by immunohistochemistry (IHC) and chromogenic in situ hybridization (CISH) [16]. Clone SP3 (Thermo Fisher Scientific) was used as the antibody for IHC, and ZytoDot 2C SPEC HER2/CEN17 Probe Kit (ZytoVision GmbH) was used for CISH. When the result on ICH was 3+ or 2+ and the ratio of *HER2*/*CEN17* on CISH was ≥ 2, the sample was assessed as positive. As a result, 26 samples (1.6%) were assessed as HER2 positive, and the HER2-positive results were significantly associated with high stages and metastases to lymph nodes. In the analysis of OS, HER2-positive patients tended to have a worse prognosis although the difference was not significant.

Valtorta E, et al. conducted a two-step research to define the HER2-positive criteria to identify patients with *HER2* amplification before conducting a clinical study of HER2-targeted treatment against unresectable or recurrent colorectal cancer (HERACLES study) [17]. In Step 1, 348 samples were finally analyzed by IHC with the HercepTest antibody (Dako) and VENTANA 4B5 antibody (Ventana), by fluorescence in situ hybridization (FISH) with PathVysion HER2 DNA Prove Kit (Abbott), and by silver-enhanced in situ hybridization (SISH; *HER2* amplification was defined as *HER2*/*CEN17* ratio ≥ 2) with Inform HER2 DNA dual color assay (Ventana). The agreement rate between FISH and SISH was 100%. When SISH was used as gold standard, false-negative results were fewer on IHC with VENTANA 4B5 than IHC with the HercepTest. When the cutoff for the proportion of positive cells was set at 50% on IHC with VENTANA 4B5, a good performance was obtained with the accuracy of 96.7%, the sensitivity of 100%, and the specificity of 94.1%. While 44

of the 348 patients had *KRAS* exon 2 mutation, no patient had *HER2* amplification or IHC3+. Based on the results of Step 1, the HER2-positive criteria were defined as "IHC3+ on IHC with VENTANA 4B5" or "IHC2+ on IHC with VENTANA 4B5 and the presence of amplification on FISH" in at least 50% of cells. In Step 2, 830 patients with *KRAS* wild-type unresectable or recurrent colorectal cancer were screened to validate the HER2-positive criteria defined in Step 1. A good performance was obtained with the accuracy of 96.7%, the sensitivity of 100%, the specificity of 96.6%, the negative predictive value of 100%, and the positive predictive value of 61%.

Richman SD, et al. assessed the HER2 status by IHC and FISH in tissue microarray (TMA) samples from 1914 patients in the QUASAR study (a prospective clinical study in patients with Stages II and III colorectal cancer) and from 1342 patients in the FOCUS study and PICCOLO study (prospective clinical studies in patients with unresectable or recurrent colorectal cancer) [18]. In the research, samples with a strongly positive result on IHC (A0485 [DAKO]) were defined as HER2 positive, and 1.3% of patients at Stages II–III and 2.2% of patients with unresectable or recurrent cancer were assessed as HER2 positive. It was also indicated that HER2 expression was significantly related to *KRAS/BRAF* wild-type tumor. No relationship of HER2 expression to the OS or PFS was observed in the cohort of the research.

In Japan, several genetic abnormalities including *HER2* amplification in gastrointestinal cancer are analyzed by next-generation sequencing (NGS) in the SCRUM-Japan GI-SCREEN. In the GI-SCREEN, the case with a gene copy number of ≥ 7 in a sample with a tumor content of ≥ 50% is defined as a patient with amplification. The results of the interim analysis in unresectable and recurrent cancers were presented by Kato, et al. at the American Society of Clinical Oncology (ASCO) Annual Meeting in 2016, and *HER2* amplification was observed in 11 (4.1%) of 266 patients with unresectable or recurrent cancer[19]. In 10 of the 11 patients, no mutation was found on *RAS* or *BRAF* genes.

As shown above, several large-cohort studies have indicated that 1.3%–4.1% of patients with colorectal cancer have HER2 overexpression or *HER2* amplification. It is also shown that *HER2* amplification is observed more frequently in patients with wild-type *RAS*/*BRAF*.

2.3.3 Relationship between *HER2* amplification and resistance to anti-EGFR antibody drugs

Because HER2 proteins form heterodimers with EGFR proteins to activate downstream signals, it is suggested that *HER2* amplification may be involved in resistance to anti-EGFR antibody drugs. In fact, some reports have shown the results that support the possibility.

In a nonclinical study by Bertotti A, et al. with the patient-derived xenograft (PDX) model generated from 85 samples of colorectal cancer, *HER2* amplification was observed in 3 samples, and

HER2 amplification and *KRAS*, *NRAS*, *BRAF*, and *PIK3CA* mutations were mutually exclusive. All of the 3 samples were cetuximab resistant[20]. It was also indicated that the combination of cetuximab + lapatinib or pertuzumab + lapatinib had an antitumor effect on the PDX model with *HER2* amplification.

Martiv V, et al. analyzed *HER2* amplification by FISH in 170 patients with unresectable or recurrent colorectal cancer treated with cetuximab or panitumumab, and *HER2* amplification (*HER2*/*CEP17* ≥ 2) in 90% or more of the cells was observed in 7 patients[21]. It was also indicated that the effect of anti-EGFR antibody drugs was significantly lower in patients with *HER2* amplification than in the other patients (median PFS: 2.5 and 6.7 months, respectively, hazard ratio [HR] 3.65, $p = 0.0026$; median OS: 4.2 months and 13 months, respectively, HR 5.05, $p = 0.0002$).

Based on the results in nonclinical and clinical studies shown above, *HER2* amplification in unresectable and recurrent colorectal cancers is a driver mutation that is involved in resistance to anti-EGFR antibody drugs and is considered a target with a substantial unmet medical need that should be developed as a new therapeutic strategy.

2.3.4 Development of HER2-targeted treatment against HER2-positive unresectable or recurrent colorectal cancer（Table 2.b）

Table 2.b Clinical studies of HER2-targeted treatment for HER2-positive metastatic colorectal cancer

Research	Phase	Number of cases	Treatment regimen	Results
Clark JW, et al. [22]	II	21	Trastuzumab<br>+ FLOX	ORR 24%<br>Median DoR 4.5 months
Ramanathan RK, et al. [23]	II	7	Trastuzumab<br>+ irinotecan	ORR 71%
HERACLES study [24]	II	27	Trastuzumab<br>+ lapatinib	ORR 30%
MyPathway study [39]	II	34	Trastuzumab<br>+ pertuzumab	ORR38%

With regard to the development of treatment against HER2-positive unresectable or recurrent colorectal cancer, two clinical studies were conducted in the early 2000s to investigate the effect of trastuzumab on HER2-positive unresectable or recurrent colorectal cancer, and the results were reported. In the study by Clark JW, et al., the efficacy of trastuzumab + FLOX therapy was evaluated in 21 previously-treated patients with HER2 IHC 2+ or 3+, unresectable or recurrent colorectal cancer. The ORR was 24% (5 of 21 patients), and the median DoR was 4.5 months[22]. In the study by Ramanathan et al., the efficacy of trastuzumab + irinotecan was evaluated in 7 patients with HER2 IHC 2+ or 3+, unresectable or recurrent colorectal cancer who were previously untreated or received one regimen, and 71% of patients (5 of 7 patients) showed objective response[23]. Although trastuzumab in combination with chemotherapy was effective to some extent against HER2-positive unresectable or recurrent colorectal cancer in the two studies, subsequently it was not developed.

Based on the nonclinical study with the PDX model mentioned above, a Phase II study, the HERACLES study, was designed to investigate the efficacy of trastuzumab + lapatinib on *KRAS* exon 2 wild-type and HER2-positive unresectable or recurrent colorectal cancer that was refractory to standard treatment[24]. In the study, 914 patients with *KRAS* exon 2 wild-type unresectable or recurrent colorectal cancer were screened with the HERACLES criteria defined in the study by Valtorta E, et al. mentioned above, and 48 patients (approximately 5%) were assessed as HER2 positive. Of them, 27 patients were enrolled in the study, and 8 patients (30%) had objective response after treatment with trastuzumab + lapatinib. The results of the HERACLES study suggested the efficacy of combination therapy with a HER2-targeted drug against HER2-positive unresectable or recurrent colorectal cancer, but subsequently the development of the trastuzumab + lapatinib therapy against HER2-positive unresectable or recurrent colorectal cancer has made little progress.

2.3.5 Concurrent therapy of trastuzumab and pertuzumab against HER2-positive unresectable or recurrent colorectal cancer

2.3.5.1 Overview of trastuzumab

Trastuzumab exerts its antitumor effect by binding specifically to the ECD subdomain IV of HER2 and exhibiting antibody dependent cell mediated cytotoxicity (ADCC) acting on natural killer cells and monocytes[25]. Trastuzumab also binds to the HER2 receptors on the surface of cells, reducing cell growth signals, and as a result, it is thought to have a mechanism that directly suppresses cell growth (Chugai Pharmaceutical in-house materials).

In the clinical development of trastuzumab, first, HER2-positive metastatic breast cancer was targeted, and in a Phase III study (the H0648g study) and Phase II study (the M77001 study),

combination therapy with trastuzumab and chemotherapy significantly prolonged progression-free survival and overall survival compared to chemotherapy alone[26, 27]. Next, Phase III studies in HER2-positive early phase breast cancer (the NSABP B-31 study, NCCTG N9831 study, HERA study, and BCIRG006 study) were conducted, and significantly prolonged progression-free survival was found[28-30]. In addition, a Phase III study of HER2-positive metastatic gastric cancer (the ToGA study) showed significantly prolonged progression-free survival and overall survival[31]. Based on the evidence, trastuzumab is widely used as standard treatment against HER2-positive early-stage breast cancer, advanced recurrent breast cancer, and advanced gastric cancer.

2.3.5.2 Over view of pertuzumab

It is thought that pertuzumab specifically binds to the dimerization domain (extracellular Subdomain II) of HER2 to block the heterodimerization of HER2 with other HER family members (HER1, HER3, and HER4), resulting in the inhibition of intracellular signal transduction pathways (MAPK and PI3K-AKT) to cause the arrest of cell proliferation and apoptosis. Like trastuzumab, pertuzumab is known to induce antibody-dependent cellular cytotoxicity.

In the clinical development of pertuzumab, the efficacy and safety of monotherapy were originally evaluated although no sufficient efficacy was obtained. Pertuzumab in combination with trastuzumab showed a synergistic antitumor effect in a nonclinical study with the model to which human breast cancer cell line, KPL-4, highly expressing HER2 was transplanted[32]. The effect was probably caused because pertuzumab in combination with trastuzumab blocked HER2 signals more comprehensively. Based on the result, the combination of pertuzumab and trastuzumab was administered to patients with HER2-positive metastatic/recurrent breast cancer who were previously treated with trastuzumab in the BO17929 study, and the results suggested that it was effective[33]. In the study, the dosage of pertuzumab was 840 mg as the initial dose and 420 mg every 3 weeks (q3w) as the maintenance dose. Following the study, pertuzumab in combination with the traditional standard treatment (trastuzumab + docetaxel) was administered to patients with HER2-positive metastatic/recurrent breast cancer in a multinational Phase III study including Japan (Study WO20698; hereinafter called the CLEOPATRA study), and a significant add-on effect was observed in the primary endpoint, PFS (the median PFS was prolonged to 18.5 months from 12.4 months) as well as in the OS[34]. The subsequent follow-up analysis demonstrated that the median OS was prolonged to 56.5 months from 40.8 months. As a result, the three-drug combination of trastuzumab, docetaxel, and pertuzumab has become the standard in the first-line treatment against HER2-positive metastatic/recurrent breast cancer[35].

Combination therapy with trastuzumab for HER2-positive metastatic gastric cancer is also under development, and a Phase III clinical study (the JACOB study) is currently underway.

2.3.5.3 Combination therapy with trastuzumab and pertuzumab

Both these antibody drugs bind to HER2, but as they bind to HER2 at different sites, they are considered to have different mechanisms of action. Trastuzumab exerts an antitumor effect by binding to ECD subdomain IV of HER2, causing an ADCC action and reducing the HER2 signal. Pertuzumab, which binds to ECD subdomain II of HER2, can sterically hinder the dimerization of HER2 with other HER2 molecules (homodimerization) and the dimerization of HER2 with different molecules such as HER3 (heterodimerization), which cannot be hindered by trastuzumab. Thus, the antibodies trastuzumab and pertuzumab, which target HER2, have complementary mechanisms of action.

In vitro studies have shown that the combination of trastuzumab and pertuzumab has synergistic activity[36]. Accordingly, we investigated this combination therapy in a human tumor xenograft model involving transplantation of each of two types of cell lines overexpressing HER2, KPL-4 (derived from breast cancer) and Calu-3 (derived from non-small cell lung cancer). The results demonstrated that in both xenograft models of Calu-3 and KPL-4, the combination of trastuzumab and pertuzumab showed a synergistic antitumor effect[32]. In addition, the combination of trastuzumab and pertuzumab completely inhibited progression of the tumor due to metastasis of KPL-4 in the host. On top of this, in a KPL-4 tumor xenograft model that showed disease progression with trastuzumab monotherapy, adding pertuzumab to trastuzumab therapy was shown to strengthen the antitumor effect.

2.3.5.4 Significance and clinical positioning of combination therapy with trastuzumab and pertuzumab for HER2-positive metastatic colorectal cancer

As stated above, *HER2* amplification is a driver genetic alteration found with a frequency of 2% to 3% in colorectal cancer. It has been reported that HER2-positive colorectal cancer has a worse prognosis than HER2-negative colorectal cancer[16], and it is likely that there is a high degree of unmet medical need for development of new treatments for HER2-positive metastatic colorectal cancer[20]. Given that the “Summary of Patient Survey, 2014” in the “White Paper & Reports” by the Ministry of Health, Labour and Welfare reports that the total number of patients with “malignant neoplasm of colon and rectum” is 261,000, and that the frequency of HER2-positive is approximately 2%–3%, the number of patients with HER2-positive colorectal cancer in Japan is estimated to be 5,220 to 7,830. The number of patients with HER2-positive colorectal cancer suitable for drug therapy is thought to be even lower.

The results of investigation of therapeutic effect in a pre-clinical study using a patient-derived xenograft (PDX) model of colorectal cancer with *HER2* amplification found no antitumor effect with trastuzumab monotherapy, but showed that there was a synergistic antitumor effect from the combination of trastuzumab and lapatinib[37]. It was also shown that trastuzumab monotherapy could

not sufficiently suppress phosphorylation of EGFR in colorectal cancer-derived cells with *HER2* amplification, but that in combination with lapatinib, phosphorylation of EGFR was suppressed, which also resulted in suppression of activation of downstream signals[37].

No pre-clinical studies of combination therapy with trastuzumab and pertuzumab for colorectal cancer with *HER2* amplification have been conducted. However, in a pre-clinical study using a cell line of gastric cancer with *HER2* amplification, combination therapy with trastuzumab and pertuzumab suppressed phosphorylation of EGFR and HER3 in addition to HER2, and was shown to have a synergistic tumor effect. Therefore, it is expected to have a similar therapeutic effect on colorectal cancer with *HER2* amplification[38]. Actually, the interim report of a Phase II clinical study (the MyPathway study*) investigating the efficacy and safety of combination therapy with trastuzumab and pertuzumab in patients with HER2-positive metastatic colorectal cancer reports a good result of 38% (13/34 patients) for the objective response rate[39]. In addition, in the same study, while patients with wild-type *KRAS* had a high objective response rate of 52% (13/25 patients), an objective response was not found in any patients with *KRAS* mutation[39].

*The MyPathway study: A Phase II study with the basket/umbrella study design being conducted at 30 sites in the United States to evaluate the efficacy and safety of each molecular targeted therapy against advanced solid tumor with *HER2*, *EGFR*, *BRAF,* or *Hedgehog* gene abnormality. In the cohort with *HER2* genetic alterations in the MyPathway study, the efficacy and safety of combination therapy with trastuzumab and pertuzumab, which is the same regimen used in this study, is being investigated in patients with metastatic colorectal cancer who meet any of the conditions, HER2 IHC3+, either *HER2*/*CEP17* ratio > 2.0 or *HER2* copies > 6.0 under *HER2* FISH or CISH, *HER2* amplification positive under next generation sequencing (NGS), or *HER2* activating mutation positive under NGS.

Given the above, combination therapy with trastuzumab and pertuzumab may become a new highly effective standard treatment for HER2-positive metastatic colorectal cancer, for which no standard treatment has been established.

2.4 Rationales for the treatment plan

2.4.1 Treatment regimen in this study

On the basis of the above background, this study was designed to evaluate the efficacy and safety of simultaneous combination therapy with trastuzumab and pertuzumab in HER2-positive metastatic colorectal cancer. With each cycle of 3 weeks, trastuzumab will be intravenously infused every 3

weeks at the first dose of 8 mg/kg and the second and subsequent doses of 6 mg/kg. Pertuzumab will be intravenously infused every 3 weeks at the first dose of 840 mg and the second and subsequent doses of 420 mg. The administration will be repeated until each subject falls under the criteria for discontinuation. Each subject will undergo study treatment on an inpatient or outpatient basis.

2.4.2 Rationales for the dosage and treatment schedule

In this study, trastuzumab will be administered every 3 weeks at the first dose of 8 mg/kg and the second and subsequent doses of 6 mg/kg, which is the dosage for treatment against HER2-positive breast cancer and HER2-positive gastric cancer. Pertuzumab will be administered every 3 weeks at the first dose of 840 mg and the second and subsequent doses of 420 mg, which is the dosage for treatment against HER2-positive breast cancer. These dosages of trastuzumab and pertuzumab have been selected because they have been widely used as an approved treatment covered by health insurance and the safety has been established. Moreover, the efficacy of the dosages has been indicated in the MyPathway study.

2.5 Posttreatment

Posttreatment in the subjects who discontinue study treatment is not specified.

The reasons are as follows. Expected posttreatment is treatment with regorafenib and trifluridine-tipiracil combination or another study treatment with a novel drug. Because these drugs are selected depending on each subject’s condition and request as well as the policy of each site, it is difficult to determine posttreatment in a single uniform way. Furthermore, posttreatment does not affect the evaluation of the primary endpoint, ORR. However, details in posttreatment will be investigated in each subject.

2.6 Study design

This is an open-label, single-arm, multicenter, Phase II study to evaluate the efficacy and safety of the concurrent therapy of trastuzumab and pertuzumab in patients with HER2-positive unresectable or recurrent colorectal cancer who are refractory or intolerant to standard chemotherapy. ORR will be the primary endpoint for efficacy evaluation. In accordance with the statistical analysis plan specified separately, the efficacy of study treatment will be analyzed by comparing the obtained data with the control data from the natural history follow-up group and from patients with HER2-positive unresectable or recurrent colorectal cancer in the SCRUM-Japan registry. Data in the SCRUM-Japan registry are collected in compliance with the Ethical Guidelines for Medical and Health Research Involving Human Subjects (it is not a study in accordance with the GCP Ordinance). When a promising result is obtained in this study, the marketing approval application of the concurrent therapy of trastuzumab and pertuzumab, which have not been approved in Japan, against a rare

disease of HER2-positive unresectable or recurrent colorectal cancer will be discussed with the pharmaceutical company that provides the study drugs.

2.6.1 Target patients

2.6.1.1 Treatment lines

This study targets patients with *RAS* wild-type metastatic colorectal cancer refractory or intolerant to standard chemotherapy who are HER2-positive. According to the 2016 physicians' guidelines for treatment of colorectal cancer (edited by the Japanese Society for Cancer of the Colon and Rectum), the chemotherapy for metastatic colorectal cancer varies depending on whether *RAS* gene mutation is present. A causative factor for this is that retrospective stratified analysis of multiple large-scale clinical studies has shown that the effect of anti-EGFR antibody drugs varies depending on whether *RAS* genes are present, and the package inserts of the two anti-EGFR antibody drugs approved for metastatic colorectal cancer in Japan also state as a "Precautions related to indications" that "indicated patients should be selected after taking into account whether *RAS* (*KRAS* and *NRAS*) gene mutation is present." The guidelines mentioned above present a treatment algorithm indicating strong treatment for *RAS* wild-type metastatic colorectal cancer, where combination therapy with fluorinated pyrimidine metabolism antagonists, oxaliplatin, irinotecan, angiogenic inhibitors, and anti-EGFR antibody drugs, followed by treatment using regorafenib and trifluridine-tipiracil hydrochloride, but regorafenib and trifluridine-tipiracil hydrochloride both have median survival periods that are only slightly different from symptomatic therapy (best supportive care), and it is stated that the risks and benefits should be taken into account when making a selection. On the other hand, in the MyPathway study in advanced solid cancer refractory or intolerant to standard treatment, the objective response rate of combination therapy with trastuzumab and pertuzumab in patients with HER2-positive colorectal cancer was reported to be 52% (13/25 patients) in patients with wild-type *KRAS*, and thus it is expected to have a far greater therapeutic effect than regorafenib and trifluridine-tipiracil hydrochloride, which are reported to have a response rate of 1% to 2%. In addition, like KRAS, NRAS is a RAS protein isoform that binds to GTP protein to change to an activated form by stimulation of upstream proteins such as EGFR, and then binds to 20 types of effector proteins such as RAF, PI3K, and RALGDS, activating the downstream signal cascade. The *NRAS* gene mutation is considered to have a similar function to the *KRAS* gene mutation[40]. In fact, it has been discovered that in metastatic colorectal cancer, the *NRAS* gene mutation, like the *KRAS* gene mutation, is related to resistance to anti-EGFR antibody drugs. In the Phase II clinical study (the MyPathway study) investigating the efficacy and safety of combination therapy with trastuzumab and pertuzumab in patients with HER2-positive metastatic colorectal cancer, objective response was not found in even one patient with *KRAS* gene mutation. Although we do not have data relating to the efficacy of combination therapy with trastuzumab and pertuzumab depending on

whether *NRAS* gene mutation is present, it is thought that in treatment targeting HER2, which is in the same human epidermal growth factor receptor (HER) family as EGFR, *NRAS* gene mutations also show the same resistance as *KRAS* gene mutations, and therefore we restricted the patients in this study to patients whose *KRAS* genes and *NRAS* genes were both wild-type. Accordingly, this study targets patients with wild-type *RAS* who are refractory or intolerant to anti-EGFR antibody drugs.

The eligibility criteria for this study do not include restrictions on the history of treatment with angiogenic inhibitors. Bevacizumab (the E3200 study and ML18147 study)[41, 42], ramucirumab (the RAISE study) [43], and aflibercept beta (the VELOUR study) [44] are each recognized to have a significant add-on effect on overall survival period relative to chemotherapy alone in second-line treatment for metastatic colorectal cancer and have received manufacturing and marketing approval in Japan (aflibercept beta has not been included in the NIH price listing). However, adverse events such as thromboembolism and gastrointestinal perforation have been reported in relation to these angiogenic inhibitors, and in some patients, cautious administration is encouraged. The 2016 physicians' guidelines for treatment of colorectal cancer (edited by the Japanese Society for Cancer of the Colon and Rectum) also recommends for bevacizumab and ramucirumab that "if it is determined that the patient is not suitable for molecular targeted drugs, for example because of the presence of serious concurrent disease or the state of the tumor, administer chemotherapy alone[45]." These angiogenic inhibitors also target vascular endothelial growth factor (VEGF) or vascular endothelial growth factor receptor (VEGFR) and have different mechanisms of action from trastuzumab and pertuzumab, the study drugs in this study, and therefore whether or not there is a history of treatment with angiogenic inhibitors is unlikely to influence the efficacy or safety of the study treatment. In fact, in the HERACLES study in patients with HER2-positive metastatic colorectal cancer, history of treatment with angiogenic inhibitors was also not restricted in the eligibility criteria[24]. Accordingly, we determined that it was reasonable to include patients in this study who do not have a history of angiogenic inhibitors, for reasons such as concurrent conditions, and we decided not to restrict history of treatment with angiogenic inhibitors in the eligibility criteria. In actual clinical practice, these angiogenic inhibitors are administered to many patients, and in the HERACLES study mentioned above, 74% of patients had a history of treatment with angiogenic inhibitors. It is predicted that many of the patients enrolled in this study will also have a history of treatment with these angiogenic inhibitors.

The expected treatment histories of the target patients in this study are shown in Figure 2.a.

Figure 2.a Prior treatment of target patients in the TRIUMPH study

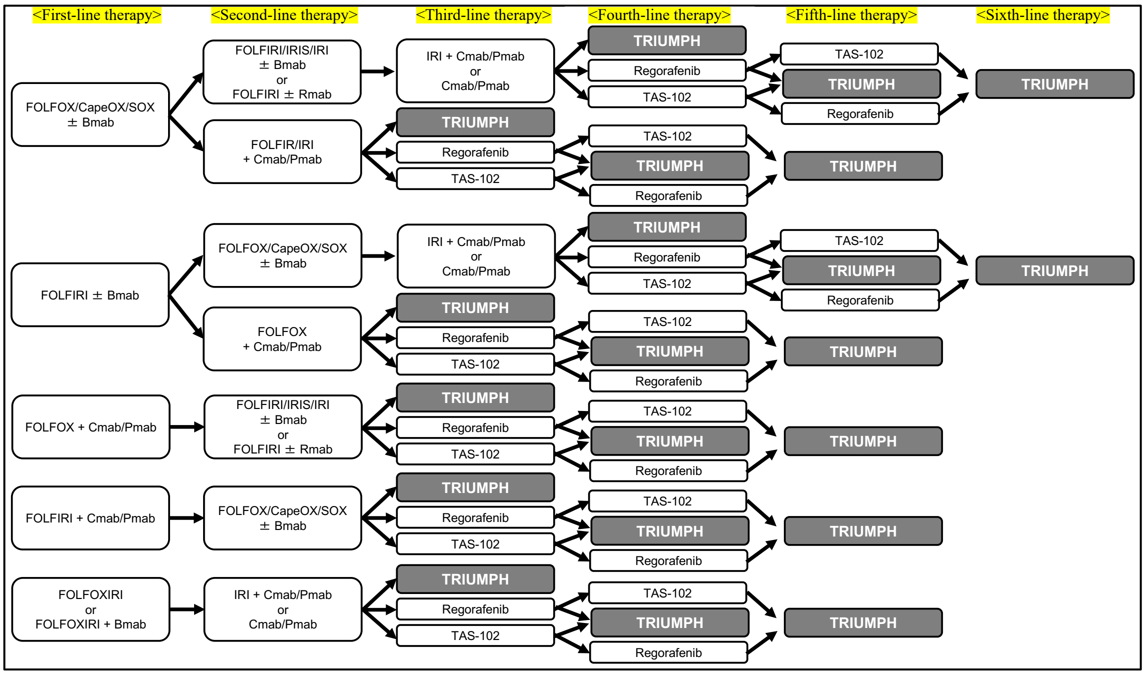

2.6.1.2 Patients with HER2-positive tumor confirmed by tissue analysis

There are no established test methods or assessment criteria for determining an HER2-positive result in colorectal cancer. At present, in breast cancer and gastric cancer, HER2 tests and assessment criteria using immunohistochemical staining (IHC) and *in situ* hybridization (ISH) are set down in breast cancer HER2 pathological diagnosis guidelines and gastric cancer HER2 pathological diagnosis guidelines respectively, and patients to administer trastuzumab (breast cancer and gastric cancer) or pertuzumab (breast cancer) are selected on the basis of results assessed using both IHC and ISH. This suggests that an HER2 test using a combination of IHC and ISH will be useful as a test for assessing eligibility for simultaneous combination therapy with trastuzumab and pertuzumab for colorectal cancer. Given the above, we decided to use an HER2 test using a combination of IHC and ISH when assessing eligibility for this study.

In this study, to reduce the risk of false negative results for patients in whom the study treatment would be effective, we selected the assessment criteria corresponding to breast cancer, “HER2 IHC score 3+” or “FISH positive (*HER2*/*CEP17* ratio ≥ 2.0)” for an HER2-positive result in colorectal cancer. As this study targets patients with a history of treatment with anti-EGFR antibody drugs, in principle we will check that they have wild-type *RAS* based on the test results using an approved extracorporeal diagnostic agent for the purpose of assessing eligibility for anti-EGFR antibody drug therapy.

2.6.1.3 Patients with HER2-positive tumor confirmed by analysis of blood sample

In recent years, progress has been made in the development of analysis of circulating tumor DNA (ctDNA) in the blood (liquid biopsy) as a non-invasive method for extracting overall genome abnormalities from a tumor with unevenly distributed genome abnormalities. In this study, we will perform a liquid biopsy using Guardant360, developed by Guardant Health, and include patients assessed as having *HER2* amplification (++ or +++) in the definition of "patients with HER2-positive metastatic colorectal cancer," and target them for evaluation of the efficacy and safety of the study drug.

Guardant360 is a panel that detects 73 cancer-related genetic abnormalities from ctDNA extracted from blood samples using proprietary digital sequencing technology developed by Guardant Health that reduces the noise accompanying sequencing for high-sensitivity detection of genetic abnormalities. In an investigation of the appropriateness of analysis compared with total exome sequencing performed at a CLIA and CAP certified laboratory (Ambry Genetics), tests showed a high level of performance, with a sensitivity of 99.9% and positive rate of 99.6% for single nucleotide variation (SNV) and a sensitivity of 95.0% and positive rate of 100% for copy number alteration (CNA) [46]. In research where liquid biopsy using Guardant360 was performed in 100 patients with breast cancer, *HER2* amplification was found in 7 patients, but a HER2-positive result was found from IHC or FISH in all patients[47]. When these 7 patients were treated with HER2 targeting drugs (including trastuzumab, lapatinib, pertuzumab, and trastuzumab emtansine), a response was found in 6 patients[47]. A case of 1 patient with metastatic colorectal cancer in whom *HER2* amplification was detected with Guardant360, who received trastuzumab and lapatinib combination therapy and achieved a response was also reported (Guardant Health in-house materials).

Accordingly, we considered that combination therapy with trastuzumab and pertuzumab can be expected to be effective in patients with metastatic colorectal cancer in whom *HER2* amplification is detected by liquid biopsy using Guardant360. This study targets *RAS* wild-type and HER2-positive patients, and in the case of patients assessed as eligible for this study from the results of blood sample analysis, those assessed as HER2-positive and *RAS* wild-type from the results of a Guardant360 liquid biopsy will be included in the scope of target patients.

The target patients in this study are shown in Table 2.c.

Table 2.c Target patients in this study

Target patients	Tumor tissue
	Wild-type *RAS*

		HER2-positive	HER2-negative, unevaluable, or not evaluated
Blood sample (ctDNA analysis)	HER2-positive and wild-type *RAS*	A	C
	HER2-negative, *RAS* mutation, unevaluable, or not evaluated	B	–

2.6.2 Endpoints

The endpoints of this study are shown below.

Primary endpoint:

Confirmed objective response rate（ORR）assessed by investigator review

The following two sets will be analyzed (analysis sets in the primary analysis are specified in Chapter 12).

- Patients with HER2 positive tumor confirmed by tissue analysis (A+B in Table 2.c)
- Patients with HER2 positive and *RAS* wild-type tumor confirmed by analysis of blood sample (A+C in Table 2.c)

Secondary endpoints:

The following items were evaluated for each of “patients with HER2 positive tumor confirmed by tissue analysis (A+B in Table 2.c)” and “patients with HER2 positive and *RAS* wild-type tumor confirmed by analysis of blood sample (A+C in Table 2.c).”

- Progression-free survival (PFS)
- Duration of response (DoR)
- Time to treatment failure (TTF)
- Disease control rate (DCR)
- Overall survival (OS)
- Confirmed ORR assessed by central review*

- Percentage change in sum of tumor diameters
- Incidences of adverse events
- ORR and PFS in the pretreatment regimens containing an anti-EGFR antibody drug (cetuximab or panitumumab)

  *Central assessment of objective tumor response may not be performed for reasons such as cost.

2.6.3 Rationales for endpoints

The results of analysis of randomized comparative studies in patients with metastatic colorectal cancer have revealed that reducing the tumor volume improves the symptoms experienced by patients. In the Phase III clinical study (the CO.17 study) that evaluated the effect of cetuximab in patients with metastatic colorectal cancer, significant greater improvements in QOL, pain, and fatigue were found in patients showing a response compared to patients not showing a response[48]. Also, in the Phase III clinical study (the CRYSTAL study) that evaluated an add-on effect from cetuximab in FOLFIRI therapy in patients with metastatic colorectal cancer, significantly greater improvements in symptoms were found in patients showing a response[49].

For the above reason, we consider that achieving a response in patients with metastatic colorectal cancer is clinically meaningful, and therefore we selected the objective response rate as the primary endpoint in this study. Investigators will make this assessment, and confirmation of response will be mandatory, to ensure that the assessed results are not due to measurement error.

The objective of this study is to evaluate the efficacy of the study treatment in each of "patients with HER2 positive tumor confirmed by tissue analysis (IHC or FISH)" and "patients with HER2 positive tumor confirmed by analysis of blood sample (liquid biopsy)," we selected the response rates in each of "patients with HER2 positive tumor confirmed by tissue analysis (A+B in Table 2.c)" and "patients with HER2 positive and *RAS* wild-type tumor confirmed by analysis of blood sample (A+C in Table 2.c)" as the primary endpoint.

We selected the following items in each of "patients with HER2 positive tumor confirmed by tissue analysis (A+B in Table 2.c)" and "patients with HER2 positive and *RAS* wild-type tumor confirmed by analysis of blood sample (A+C in Table 2.c)" as secondary endpoints: progression-free survival, duration of response, time to treatment failure, disease control rate, overall survival, percentage change in sum of tumor diameters, objective response rate (this may not be performed for reasons such as cost), incidence of adverse events, and objective response rate in the pretreatment regimens containing an anti-EGFR antibody drug (cetuximab or panitumumab).

2.6.4 Clinical hypothesis and rationales for the sample size

2.6.4.1 Clinical hypothesis

The clinical hypothesis is that the HER2-targeted treatment with trastuzumab and pertuzumab has a superior antitumor effect to that of standard treatment in patients with HER2-positive unresectable or recurrent colorectal cancer who are refractory or intolerant to treatment with fluorinated pyrimidine antimetabolites, oxaliplatin, irinotecan, cetuximab, or panitumumab.

2.6.4.2 ORR の閾値と期待値の設定根拠

The standard treatment for the target patients in this study, who are patients refractory or intolerant to fluorinated pyrimidine metabolism antagonists, oxaliplatin, irinotecan, cetuximab, or panitumumab, is treatment using regorafenib or trifluridine-tipiracil hydrochloride, but the objective response rate of these treatments is approximately 1%–2%[4, 5]. The response rates of these salvage line treatments when limited to HER2-positive patients is not clear. On the other hand, in the HERACLES study and the MyPathway study, the objective response rates in HER2-positive patients were 30% (8/27 patients, 95% confidence interval 14%–50%) and 38% (13/34 patients, 95% confidence interval 22%–56%) respectively[24, 39].

Although the objective response rate of regorafenib or trifluridine-tipiracil hydrochloride when limited to HER2-positive patients has not been reported, there are no reports that these treatments have a striking effect when their scope is limited to HER2-positive patients, and therefore in this study, we estimated that the objective response rate if HER2-positive patients received standard treatment (regorafenib or trifluridine-tipiracil hydrochloride) would be no higher than 5%, and we set this as the threshold level.

In the HERACLES study investigating the efficacy and safety of trastuzumab and lapatinib combination therapy in patients with *KRAS* wild-type and HER2-positive metastatic colorectal cancer, the centrally assessed objective response rate was 30% (8/27 patients), but response was confirmed in 7 of the 8 patients who showed a response, and therefore the confirmed objective response rate was 26% (7/27 patients) [22]. On the other hand, in the MyPathway study investigating the efficacy and safety of combination therapy with trastuzumab and pertuzumab in patients with HER2-positive metastatic colorectal cancer, the objective response rate was 52% (13/25 patients) in *KRAS* wild-type patients, but as this objective response rate was not assessed or confirmed by investigators, it is thought that the confirmed objective response rate is lower than 52%[37]. It is likely that on the treatment line for patients with metastatic colorectal cancer, who are the target patients in this study, an objective response rate of 30% is a clinically meaningful value. On the basis of the above considerations, we have set 30% as the expected value for the response rate in this study. We set 30% as the expected value of the response rate with reference to the studies mentioned above, the

HERACLES study that investigated the efficacy and safety of HER2 targeted therapy and the MyPathway study that investigated the efficacy of combination therapy similar to the treatment in this study.

To collect data in HER2-positive patients who do not undergo HER2-targeted treatment, the natural history follow-up group will be included in this study. With the data from the natural history follow-up group and from the SCRUM-Japan GI-SCREEN, the appropriateness of the threshold will be evaluated.

2.6.4.3 Rationales for the target sample size

For each of the analysis sets, “patients with HER2 positive tumor confirmed by tissue analysis (A+B in Table 2.c)” and “patients with HER2 positive and *RAS* wild-type tumor confirmed by analysis of blood sample (A+C in Table 2.c),” the threshold and expected value of the objective response rate for study treatment, which is the primary endpoint, were set at 5% and 30%, respectively, with a one-sided significance level of 2.5% and a power of 80%, and the target sample size for each analysis set was set at 18 (*on the basis of a precise method based on the binomial distribution). When at least 4 of the 18 subjects have an objective response, the result will be considered statistically significant. In each analysis set, if subjects are enrolled at a good rate, whether the power can be raised to 90% (whether the target sample size can be increased to 25) will be evaluated during the study.

2.6.4.4 Criteria for raising the target sample size

Because this is a single-arm study, efficacy data of some subjects (in particular, whether or not a response occurred) will be shared between researchers, and if the selection of enrolled patients is selected non-randomly on the basis of this investigation, this may introduce bias into the study results. With this point in mind, the decision on increasing the target sample size will be made only on the basis of the number of enrolled patients found from periodic monitoring. If 13 patients have been enrolled by the time 1 year after enrollment of the first patient in each analysis set (or if it is likely that 13 patients will be enrolled soon), researchers will consider increasing the sample size.

2.6.5 Expected number of enrolled patients and the periods of enrollment and follow-up

The period required to enroll 18 patients in the FAS has been estimated to be 2 years. The planned enrollment period is 25 months, from December 2017 to December 2019. If subjects are enrolled at a good rate after the start of the study, the target sample size will be increased to 25. Although the target of this study is patients with a rare disease of HER2-positive unresectable or recurrent colorectal cancer, it is considered possible to complete the enrollment within the planned period because the screening for HER2-positive patients will be performed efficiently in patients with

HER2 amplification who are identified by the SCRUM-Japan GI-SCREEN, where more than 1000 patients with gastrointestinal cancer are registered every year. In the above study by Kato, et al. reported in the ASCO Annual Meeting in 2016, *HER2* amplification was detected in 11 (4.1%) of 266 patients with unresectable or recurrent colorectal cancer in the 9-month period from February to October in 2015. Therefore, at least 1 patient per month is expected to be screened. Because screening for target patients will be performed in both the patients with *HER2* amplification who are already identified and those who will newly be identified, it is considered possible to enroll the planned number of patients.

The planned observation period will be until March 2022.

2.6.6 Control group

Because of the rarity of target patients for this study, it is not feasible to perform a confirmatory randomized comparison study, and therefore it is important to compare the efficacy data from administration of the study treatment in this study to the target patients with HER2-positive metastatic colorectal cancer with the outcome data (response rate, progression-free survival, overall survival, etc., hereafter referred as “natural history data”) of administration of other treatment (such as regorafenib or trifluridine-tipiracil hydrochloride). Accordingly, for evaluation of the efficacy of treatment in this study, we plan to gather natural history data by two methods and use it as control group data for comparison.

1. In this study, natural history data will be gathered within the study in patients who have HER2 positive tumors but do not meet the eligibility criteria B or meet any of the exclusion criteria.
2. Natural history data on patients with HER2-positive metastatic colorectal cancer will be gathered from the disease registry established in the AMED Project Promoting Clinical Trials for Development of New Drugs and Medical Devices “Establishing a Disease Registration System to Contribute to the Development of New Drugs for Cancer Utilizing the Cancer Genome Screening Project for Individualized Medicine in Japan (SCRUM-Japan)” (representative: Atsushi Otsu).

In this study, we plan to perform comparison of response rate and progression-free survival with the control group. We plan to compare overall survival after the end of the follow-up period following this study. Details are specified in the analysis plan for this study (or the plan for observational research for surveying prognosis after the end of the study).

2.7 Summary of expected benefits and disadvantages from participation in the study

2.7.1 Expected benefits

As with the results in the HERACLES study and the MyPathway study, the concurrent therapy of HER2-targeted trastuzumab and pertuzumab is expected to exert an antitumor effect in patients with HER2-positive unresectable or recurrent colorectal cancer.

2.7.2 Expected risks and disadvantages

Adverse events such as diarrhea and cardiac impairment may occur due to administration of trastuzumab and pertuzumab. To minimize risks for adverse events and their disadvantages, the coordinating committee will evaluate whether actual adverse events are within the expected range. If a serious or unexpected adverse event has occurred, necessary actions will be taken, including reporting it to the response and safety evaluation committee and informing the participating sites. Because biopsy and blood sampling for the exploratory research on biomarkers may cause pain and complications such as anemia, those procedures will be performed only in the subjects who give their consents after they receive a full explanation on the risks and inconvenience.

2.8 Reasons why this is conducted as an investigator-initiated study

HER2-positive unresectable or recurrent colorectal cancer is one of the great unmet medical needs because anti-EGFR antibody treatment is ineffective, and it is urgent to develop treatment against it. Based on the results of previous nonclinical and clinical studies and the MyPathway study, the concurrent therapy of trastuzumab and pertuzumab is expected to be effective against HER2-positive unresectable or recurrent colorectal cancer. However, Chugai Pharmaceutical Co., Ltd., which is the marketing authorization holder for trastuzumab and pertuzumab in Japan, is not planning to develop them as treatment for the patient population. Because the proportion of patients with HER2-positive unresectable or recurrent colorectal cancer is very low (approximately 1.3%–4.1%) in the entire patients with colorectal cancer, it is difficult to enroll those patients in a study initiated by the company. In contrast, the coordinating investigators of this study can use the SCRUM-Japan GI-SCREEN system to perform an efficient screening for HER2-positive patients. Therefore, this study has been planned as an investigator-initiated study to evaluate the efficacy and safety of the concurrent therapy of trastuzumab and pertuzumab in patients with HER2-positive unresectable or recurrent colorectal cancer.

2.9 Accompanying research

As a research accompanying this study, an exploratory research on biomarkers will be conducted in accordance with the protocol prepared separately. The results of the accompanying research will not be included in the clinical study report (CSR).

We also plan to conduct observational research (clinical research) to follow up on information such as outcomes after the end of the observation period and perform evaluation, as accompanying research.

3 Criteria and definitions used in this study

3.1 Performance Status (Eastern Cooperative Oncology Group [ECOG] classification)

(See Appendix A)

3.2 Criteria for the assessment of adverse events

The names and grades of adverse events will be based on the Common Terminology Criteria for Adverse Events (CTCAE) version 4.0 by the Japan Clinical Oncology Group (JCOG) (CTCAE v4.0 - JCOG, corresponding to the original CTCAE v4.03/MedDRA v12.0). This study will follow the operations specified by the JCOG.

3.3 Criteria for the assessment of response rates

The objective tumor response will be assessed according to the New Response Evaluation Criteria in Solid Tumours: Revised RECIST Guideline (Version 1.1), a Japanese version translated by the JCOG (see Appendix B).

3.4 HER2-positive criteria

In the evaluation of eligibility criteria for this study, the necessary HER2 tests of tumor tissue and liquid biopsy using a blood sample, and central assessment of these results, will be performed separately from this study in the following two pieces of research.

In the “research for HER2 screening of metastatic colorectal cancer (HER2 screening),” analysis of tumor tissue will be performed at SRL by IHC using the Ventana I-VIEW Pathway HER2 (4B5) (Roche Diagnostics K.K.) and FISH using the PathVysion HER-2 DNA Probe Kit (Abbott Japan Co., Ltd.). Next, patients with an IHC score of 3+ or a FISH positive result (*HER2*/*CEP17* ratio ≥ 2.0) will be assessed as HER2 positive. The results of assessment in this clinical research will be defined as the results of central pathological assessment of HER2 tests of tumor tissue in this study.

In the “research relating to liquid biopsy of malignant tumor of the gastrointestinal tract or abdomen including colorectal cancer (the GOZILA study),” ctDNA analysis will be performed at Guardant Health by liquid biopsy of blood samples using Guardant360. Patients assessed as having *HER2* amplification (++ or +++) as a result will be assessed as HER2 positive. The results of assessment in this clinical research will be defined as the results of central assessment of liquid biopsy of blood samples in this study.

4 Inclusion and exclusion criteria

4.1 Inclusion criteria of this study

Eligibility criteria A:

1. A patient aged 20 years or above
2. A patient with metastatic colorectal cancer that is histologically diagnosed as adenocarcinoma
3. A patient who has tumor tissue with wild-type *KRAS* codons 12 and 13. If other codons than *KRAS* codons 12 and 13 (*KRAS* codons 59, 61, 117, and 146 and *NRAS* codons 12, 13, 59, 61, 117, and 146) are also analyzed, the statuses for all these *RAS* codons must be wild type.
4. A patient with cancer that meets either the following criteria I or II

   I. A patient with cancer that meets either of the following based on analysis of tumor tissue in central pathological assessment* using the HER2 IHC test and HER2 FISH test

      i. IHC3+
      ii. FISH positive (*HER2*/*CEP17* ratio $\geq$ 2.0)

   II. A patient who has *HER2* amplification (++ or +++) (HER2 positive) and wild-type *RAS*[†] based on analysis of a blood sample in central assessment* using liquid biopsy

[†]Wild-type *RAS* is defined as "Relative clonality[††] is equal to or less than 30% in each *KRAS* codon 12, 13, 59, 61, 117, and 146, and *NRAS* codon 12, 13, 59, 61, 117, and 146."

[††] relative clonality (%) = % cfDNA of a certain mutation / the highest % cfDNA x 100

*The HER2 test of tumor tissue, the liquid biopsy using blood samples and central assessment of the results will be performed in clinical research conducted separately.

5. A patient who is refractory or intolerant to fluorinated pyrimidine antimetabolites, oxaliplatin, irinotecan, cetuximab, or panitumumab* (with or without a treatment history with angiogenic inhibitors such as bevacizumab, ramucirumab and aflibercept, trifluridine-tipiracil combination, and regorafenib)

*In patients with recurrence during adjuvant chemotherapy or within 6 months after the final dose, adjuvant chemotherapy is counted as treatment history.

6. A patient with an ECOG Performance Status of 0 or 1
7. A patient with an expected survival of at least 12 weeks
8. A patient who has given written consent

Eligibility criteria B:

1. A patient who has never been treated with HER2-targeted drugs (including pan-HER-targeted drugs)
2. A patient with a measurable lesion based on the RECIST Version 1.1

3. A patient with the organ functions that meet the following:
   - Neutrophil count ≥ 1,000/mm^3
   - Platelet count ≥ 75,000/mm^3
   - Hemoglobin ≥ 8.0 g/dL
   - Serum creatinine ≤ 2.0 mg/dL, or calculated (Cockcroft-Gault equation*) or measured creatinine clearance ≥ 50 mL/min
   - Total bilirubin ≤ 2.0 mg/dL (If a patient with Gilbert's syndrome has a value of > 2.0 mg/dL, the patient can be enrolled after the coordinating committee agrees.)
   - ALT and AST ≤ 100 U/L (patients with liver metastasis: ≤ 200 U/L)

   *Cockcroft-Gault equation: Creatinine clearance value = (140 − age) × body weight (kg)/(72 × serum creatinine value) (*for women, multiplying the obtained value by 0.85 gives the creatinine clearance)
4. A patient with a left ventricular ejection fraction (LVEF) of 50% or above measured by echocardiography, multigated acquisition scan (MUGA), or magnetic resonance imaging (MRI)
5. A patient with no symptomatic brain metastasis or meningeal dissemination
6. A patient who is not breast-feeding
7. A patient who is willing to use appropriate contraception throughout study treatment and for at least 7 months after the last dose of pertuzumab or trastuzumab

   *The following methods of contraception are used.
   - Abstaining from sexual intercourse
   - Using a combination hormone contraception drug
   - For women, having only one partner, who is a man who has undergone vasectomy
   - For men, undergoing vasectomy
8. A patient without a history of cardiovascular disease shown below within 6 months before the start of study treatment
   - Acute myocardial infarction
   - Angina pectoris that requires intervention
   - Symptomatic congestive cardiac failure
9. A patient without inadequately controlled hypertension (systolic pressure ≥ 180 mmHg or diastolic pressure ≥ 100 mmHg)
10. A patient with no history of inadequately controlled arrhythmia (heart rate at rest > 100 beats per minute), ventricular arrhythmia, or severe atrioventricular block (Mobitz type II second-degree atrioventricular block or third-degree atrioventricular block)
    - A patient who has chronic atrial arrhythmia with controlled pulse rate is eligible if the patient does not have any other cardiovascular abnormality.
11. A patient with a negative result on the human immunodeficiency virus (HIV) antibody test
12. A patient with a negative result on the hepatitis B surface (HBs) antigen test

13. A patient who is not receiving long-term treatment with systemic steroids at a prednisolone equivalent dose > 10 mg/day
14. A patient with no history of other malignant tumors within 3 years before the start of study treatment. However, if a patient has a history of the lesion equivalent to carcinoma in situ or intramucosal carcinoma that is judged cured after local treatment, non-metastatic prostate cancer that does not require systemic treatment, or other solid cancers that do not require treatment or to which study treatment is not considered disadvantageous.
15. A patient who has not undergone another antitumor treatment concurrently.
    - A patient who has not undergone pretreatment (chemotherapy, molecular targeted therapy, antibody therapy, or radiotherapy) within 2 weeks before the start of study treatment
    - Pretreatment with bisphosphonates and denosumab is allowed.
16. A patient who has recovered from toxicity due to pretreatment (to Grade 1 or below or to baseline). However, patients with Grade 2 peripheral sensory neuropathy, Grade 2 alopecia, Grade 2 skin hyperpigmentation, controllable hypertension, and hypocalcemia/hypomagnesemia are eligible.
17. A patient who has not undergone major surgery within 4 weeks before the start of study treatment. However, patients who have undergone colostomy earlier than 2 weeks before the start of study treatment are eligible.
18. A patient with no other serious, clinically significant abnormalities that are acute or chronic
19. A patient who does not want study treatment

Exclusion criteria:

1. A patient who had a history of bone marrow transplantation or organ transplantation.
2. A patient who is a woman of childbearing potential and has a positive result on a serum pregnancy test.
3. A patient with active hemorrhagic lesions.
4. A patient whom the investigator judges to be unsuitable for enrollment

Patients who meet both the eligibility criteria A and B will be enrolled as target for study treatment in the study. Patients who meet the eligibility criteria A, do not meet the eligibility criteria B or meet any of the exclusion criteria, will be enrolled in the natural history follow-up group and will be investigated every 3 months for information on antitumor treatment and survival but not for adverse events.

The follow-up period in the study treatment group will be until the subject dies, is lost to follow-up, or withdraws consent, or until the end of the study observation period, whichever comes first.

4.2 Follow-up of natural history

Patients who meet the eligibility criteria A, and do not meet the eligibility criteria B or meet any of the exclusion criteria, will be enrolled in the natural history group and followed up every 3 months to obtain information about their antitumor treatment and whether they are alive. Adverse events will not be followed up. The follow-up period will be until the subject dies, is lost to follow-up, or withdraws consent, until the end of the study observation period or until the subject is enrolled in the study treatment group, whichever comes first.

5 Enrollment and allocation

5.1 Enrollment procedure

Figure 5 Flowchart of enrollment

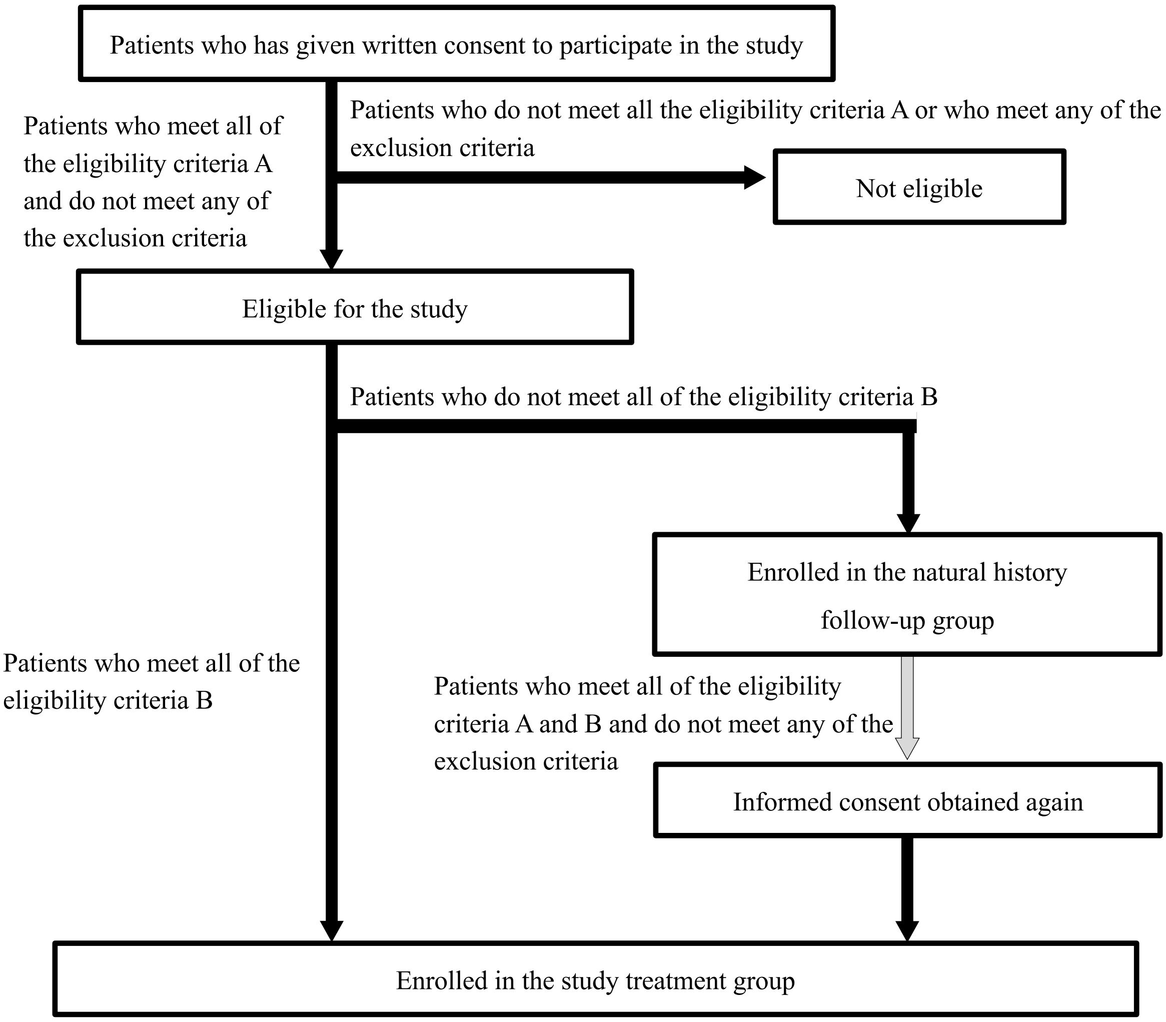

1. Study treatment group: Investigators will confirm that a patient meets all of the eligibility criteria A and B and does not meet any of the exclusion criteria and enroll the patient by the Web enrollment system.

2. Natural history follow-up group: Investigators will confirm that a patient meets all of the eligibility criteria A, does not meet any of the exclusion criteria and does not meet all of the eligibility criteria B, and enroll the patient by the Web enrollment system.

Contact information and business hours for subject enrollment

The URL for Web enrollment and other information will be informed separately in the EDC Entry Manual.

(Subjects can be enrolled 24 hours a day. If access is closed due to maintenance, etc., investigators will be notified in advance.)

■Contact information for inquiries on enrollment

Clinical Research Support Office, National Cancer Center Hospital East

TEL: 04-7133-1111 (extension 5106); E-mail: triumph_core@east.ncc.go.jp

Weekdays: 10:00 to 17:00 (closed on holidays, Saturdays and Sundays)

■Contact information for inquiries on subject inclusion criteria

Coordinating investigator (representative until March 31, 2019): Wataru Okamoto

Chief of BB/TR Support Section, Clinical Research Support Office, National Cancer Center Hospital East

6-5-1 Kashiwanoha, Kashiwa, Chiba, 277-8577, Japan

TEL: 04-7133-1111 (extension 91551); E-mail: wokamoto@east.ncc.go.jp

(From April 1, 2019)

Cancer Treatment Center, Hiroshima University Hospital

1-2-3, Kasumi, Minami-ku, Hiroshima, Hiroshima, 734-8551, Japan

TEL：082-257-5555

Coordinating investigator: Yoshiaki Nakamura

Department of Gastrointestinal Oncology, National Cancer Center Hospital East

6-5-1 Kashiwanoha, Kashiwa, Chiba, 277-8577, Japan

TEL: 04-7133-1111 (extension 91397); E-mail: yoshinak@east.ncc.go.jp

Coordinating investigator: Akihiro Sato

Head of Clinical Research Support Office, National Cancer Center Hospital East

6-5-1 Kashiwanoha, Kashiwa, Chiba, 277-8577, Japan

TEL: 04-7133-1111 (extension 91247); E-mail: asato@east.ncc.go.jp

Coordinating investigator (representative from April 1, 2019): Takayuki Yoshino

Director, Department of Gastrointestinal Oncology, National Cancer Center Hospital East

6-5-1 Kashiwanoha, Kashiwa, Chiba, 277-8577, Japan

TEL: 04-7133-1111 (extension 93011); E-mail: tyoshino@east.ncc.go.jp

5.2 Notes for enrollment

1) As there are two analysis sets in this study, after the target sample size for either of the analysis sets has been reached, enrollment of some populations may be stopped. (See "Section 5.3 Operations related to enrollment in two analysis sets")

2) The target sample size of this study may be increased, and enrollment may be continued after the initial target sample size is reached. (See "Section 2.6.4.4 Criteria for raising the target sample size")

3) Enrollment after the start of study treatment is not allowed, with no exception.

4) If the entry on the enrollment and eligibility confirmation form is insufficient, the enrollment will be withheld until all entry is completed.

5) The enrollment number will be issued after the eligibility of the patient is confirmed. The enrollment will be completed at the time of the issue of the enrolment number.

6) The subject who has been enrolled once will not be cancelled (deleted from the database), unless the subject withdraws his or her consent including consent to the use of his or her data for research. If a subject is enrolled redundantly, the information (enrollment number) at the first enrollment will be used in every instance.

7) If a patient has been enrolled in the natural history follow-up group but meets the eligibility criteria A and B, the patient may be re-enrolled in the study treatment group after providing informed consent again. If a patient is re-enrolled in the study treatment group, the information (enrollment number) for the study treatment group will be used.

8) If wrong or double enrollment is found, investigators should immediately inform the responsible person.

9) If a subject is regularly visiting another hospital or department at enrollment, investigators should inform the physician in charge about the subject's participation in this study.

5.3 Operations related to enrollment in two analysis sets

The expected enrollment in the two analysis sets in this study (A+B and A+C in Table 2.c) is unclear, and it is possible that the target sample size of one set will be reached first. In this study, if

either of the sets has reached the target sample size, patients in B (if A+B reaches the target sample size first) or C (if A+C reaches the target sample size first) will not be enrolled in the study treatment group, but will be enrolled in the natural history follow-up group. Even if the study is operated in this way, enrollment in A will be continued, so that at the time of the primary analysis, enrollment in one of the analysis sets exceeds the target sample size. The extra enrolled patients will be excluded from the primary analysis, but for reference, analysis including them will also be performed. Enrollment in excess of target sample sizes based on statistical evidence will be avoided as far as possible, but in this study, as both analysis sets contain A, it is not possible to end enrollment in only one of the sets. On the basis of the above considerations, we have determined that the best approach is to end enrollment only in the parts of the analysis sets that do not overlap (B and C).

Table 2.c Target patients in this study

Target patients		Tumor tissue	
		Wild-type *RAS*	
		HER2 positive	HER2 negative, unevaluable, not evaluated
Blood sample (ctDNA analysis)	HER2 positive and wild-type *RAS*	A	C
	HER2 negative, *RAS* mutation, unevaluable, or not evaluated	B	–

6 Treatment plan and criteria for change in treatment

Treatment and change in treatment will be performed in accordance with the description in this chapter, unless the safety of subjects is endangered. If the investigator or subinvestigator (hereinafter called investigators) considers it medically dangerous to follow the protocol, treatment should be changed according to the medical judgment of investigators.

6.1 Study treatment

The study treatment in this study is the concurrent administration of the study drugs trastuzumab and pertuzumab. First, pertuzumab will be administered and each subject will be monitored for at least 60 minutes after treatment. After this, trastuzumab will be administered over a period of at least 90 minutes and then each subject will be monitored for at least 60 minutes after treatment (as stated in Sections 6.1.1 and 6.1.2, the monitoring periods may be shortened to 30 minutes from the second dose onwards). If a subject meets the discontinuation criteria for pertuzumab only, investigators will judge whether study treatment should be continued as trastuzumab monotherapy after discussion with the coordinating committee. Pertuzumab monotherapy will not be administered because efficacy and safety have not been established in other types of cancer.

Study treatment should be started within 8 days after enrollment (the date of enrollment inclusive). If study treatment in a subject has not been started within 8 days after enrollment (the date of enrollment inclusive), investigators should inform the coordinating committee and discuss the handling of the subject. Each subject can undergo study treatment on an inpatient or outpatient basis.

The definitions of the terms used in this protocol are as follows.

- Discontinuation: To stop treatment with study drugs and not to resume it
- Cessation: To proceed the next treatment schedule without administering one or more doses of study drugs
- Cessation period: The period in which study drugs are not administered

6.1.1 Pertuzumab

- Pertuzumab will be intravenously infused on Day 1 of each treatment cycle every 3 weeks, at the dose of 840 mg in Cycle 1 and at the dose of 420 mg in subsequent cycles.
- Treatment with pertuzumab in a subject will be continued until the subject meets the discontinuation criteria specified in Section 6.2.1.
- The first dose of pertuzumab (Day 1 of Cycle 1) should be infused over at least 60 minutes. After infusion, investigators should monitor for 60 minutes whether infusion-associated

reactions (IARs) occur such as fever, chills, headache, pruritus, nausea, vomiting, and abnormal vital signs. If these symptoms occur, a decrease in the infusion rate or the interruption of treatment may be useful. Treatment can be resumed after the symptoms are alleviated. Supportive therapy with oxygen, beta-agonists, antihistamines, antipyretics, etc., may be useful for alleviating the symptoms.

- When the first dose is well tolerated by a subject, the subject can receive the subsequent infusions over 30 minutes and can be monitored for 30 minutes.
- An antipyretic, antihistamine, or steroid may be given before the infusion of pertuzumab.
- The dose reduction due to toxicity will not be allowed for pertuzumab. Treatment can be delayed due to toxicity including cardiac toxicity such as symptomatic or asymptomatic decrease in the LVEF.
- If a subject receives pertuzumab 3 weeks or more after the scheduled date of treatment, it should be given at the dose of 840 mg again. Subsequently, the dose of 420 mg will be administered every 3 weeks. The infusion period and monitoring period can be kept at 30 minutes.

6.1.2 Trastuzumab

- Trastuzumab will be intravenously infused on Day 1 of each treatment cycle every 3 weeks, at the dose of 8 mg/kg in Cycle 1 and at the dose of 6 mg/kg in subsequent cycles.
- The dose (mg) calculated from the body weight will be rounded down to 10. For example, with the body weight of 63 kg, 63 × 8 = 504 (Cycle 1) and 63 × 6 = 378 (Cycle 2) are rounded down to 10: 500 and 370 mg, respectively.
- After the start of treatment, if a change in body weight of more than 10% relative to baseline (or relative to the newly applied body weight) is found, the dose will be recalculated. The newly applied body weight is the body weight when the dose is recalculated.
- Treatment with trastuzumab in a subject will be continued until the subject meets the discontinuation criteria specified in Section 6.2.1.
- The first dose of trastuzumab (Day 1 of Cycle 1) should be infused over at least 90 minutes. After infusion, investigators should monitor for 60 minutes whether IARs occur such as fever, chills, headache, pruritus, nausea, vomiting, and abnormal vital signs. If these symptoms occur, a decrease in the infusion rate or the interruption of treatment may be useful. Treatment can be resumed after the symptoms are alleviated. Supportive therapy with oxygen, beta-agonists, antihistamines, antipyretics, etc., may be useful for alleviating the symptoms.

- When the first dose is well tolerated by a subject, the subject can receive the subsequent infusions over 30 minutes and can be monitored for 30 minutes.
- An antipyretic, antihistamine, or steroid may be given before the infusion of trastuzumab. Premedication for preventing IARs due to trastuzumab may be given before the infusion of pertuzumab.
- The dose reduction due to toxicity will not be allowed for trastuzumab. Treatment can be delayed due to toxicity including cardiac toxicity such as symptomatic or asymptomatic decrease in the LVEF.
- If a subject receives trastuzumab more than 1 week after the scheduled date of treatment, it should be given at the dose of 8 mg/kg again. Subsequently, the dose of 6 mg/kg will be administered every 3 weeks. The infusion period and monitoring period can be kept at 30 minutes.

6.2 Discontinuation criteria for study treatment

6.2.1 Discontinuation criteria for study treatment

If a subject meets any of the following criteria, study treatment in the subject should be discontinued.

1) When the primary disease has progressed (including radiologically or clinically obvious progression)
2) When a subject requests the discontinuation of study treatment
3) When Grade 4 non-hematological toxicity related to study treatment is observed (excluding transient abnormal laboratory values)
4) When a subject meets the discontinuation criteria for study treatment shown in Section 6.3, Criteria for change in study treatment
5) When treatment with trastuzumab or pertuzumab cannot be resumed within 63 days after interruption
6) When a subject becomes pregnant
7) When a subject is lost to follow-up
8) When a subject dies during study treatment
9) When investigators consider it necessary to withdraw a subject from the study for other reasons

6.2.2 Data Collection and Follow-up of Patients after Discontinuation of the protocol treatment

If a subject meets the discontinuation criteria, investigators should stop study treatment in the subject, enter the timepoint and reason of discontinuation and the subject's course on the case report form (CRF), and perform the tests specified for the post-treatment period. However, the tests may not be performed if investigators consider it impossible or unnecessary to perform specified tests from an ethical viewpoint or in consideration of the safety and benefits of the subject.

6.3 Criteria for discontinuation of the protocol treatment

Treatment with trastuzumab and pertuzumab can be interrupted for evaluating or treating the cardiotoxic adverse events shown in Section 6.3.1. It can also be interrupted due to the occurrence of other adverse events if the investigators consider that this is necessary. If an adverse event is considered likely to be related to pertuzumab alone, treatment with pertuzumab alone can be interrupted and trastuzumab alone continued. Interruption of trastuzumab alone is not allowed.

If the start of treatment is postponed, for example due to interruption, the subsequent schedule is based on taking the date of the start of treatment after the postponement to be Day 1 of the cycle. However, the dose reduction of trastuzumab and pertuzumab is not allowed.

If pertuzumab is administered 3 weeks or more after the scheduled date of treatment in any cycle, it should be given at the dose of 840 mg again.

If trastuzumab is administered more than 1 week after the scheduled date of treatment in any cycle, it should be given at the dose of 8 mg/kg again.

For continuing study treatment, it must be resumed within 3 cycles, that is, within 63 days after Day 1 of the last cycle. If treatment with trastuzumab or pertuzumab cannot be resumed within 63 days after the last dose or a subject meets the discontinuation criteria, investigators should discontinue all study treatment in the subject and follow-up the subject in accordance with Section 8.5.

6.3.1 Delay or discontinuation of treatment due to cardiac toxicity

Figure 6 Algorithm for judging the continuation and discontinuation of study treatment

based on the LVEF

Algorithm for asymptomatic decrease in the LVEF

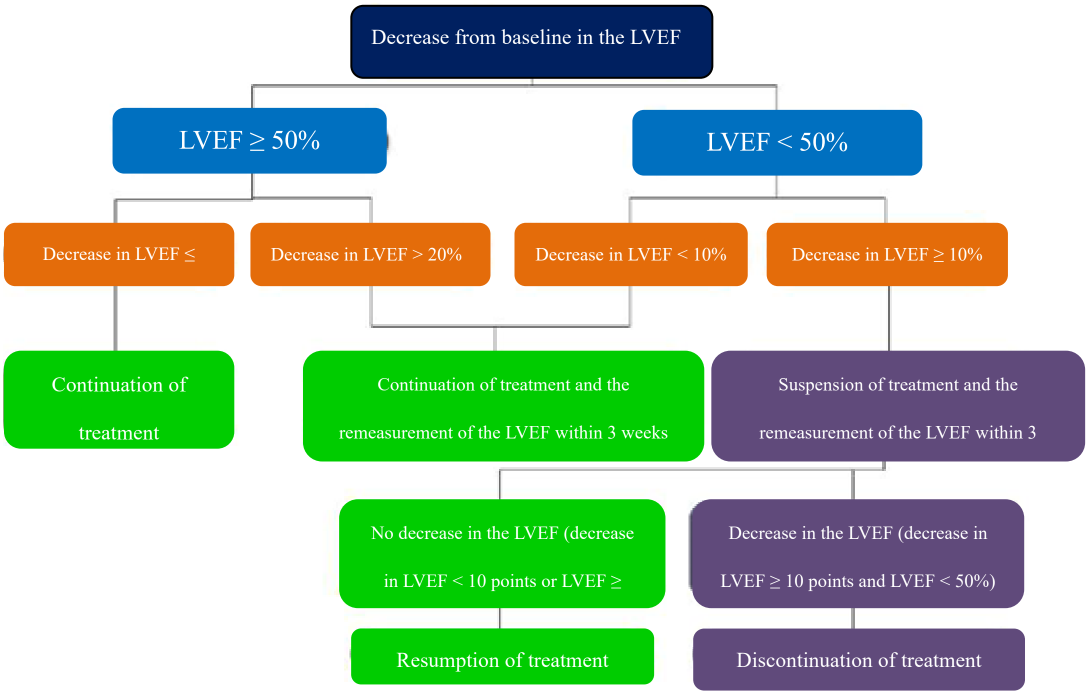

LVEF = left ventricular ejection fraction

In this study, each subject must have a LVEF of ≥ 50% at baseline. During study treatment, the LVEF should be measured once every 4 cycles or more frequently. To ensure the safety of each subject, investigators should additionally perform LVEF measurement and other appropriate examinations such as chest X-ray tests if an adverse event occurs that they consider may be associated with cardiac dysfunction. They should perform the specified evaluation of LVEF as scheduled.

If a subject is diagnosed with symptomatic left ventricular systolic dysfunction (LVSD), investigators should permanently discontinue treatment with trastuzumab and pertuzumab in the subject and enter the reason for the discontinuation of study treatment as intolerable toxicity. They should treat and monitor symptomatic LVSD in accordance with standard treatment.

At present, there are no data enough to evaluate the significance of asymptomatic decrease in the LVEF in terms of prognostic prediction. For asymptomatic subjects with an absolute LVEF value that has decreased by ≥ 10 percentage points from baseline and is < 50% in this study, investigators should temporarily discontinue treatment with trastuzumab and pertuzumab and measure the LVEF again within 3 weeks. As a result of measuring LVEF again, if a decrease in the LVEF is confirmed (absolute LVEF value that decreased by ≥ 10 percentage points from baseline and decreased to <

50%), investigators should permanently discontinue treatment with trastuzumab and pertuzumab in the subject and enter the reason for discontinuation as intolerable toxicity. Investigators should evaluate decreases in the LVEF in accordance with the algorithm in Figure 6 to judge the discontinuation or continuation of study treatment.

6.3.2 Infusion-associated reactions

Treatment with trastuzumab and pertuzumab may cause symptoms associated with the infusion, such as nausea, fever, diarrhea, chills, fatigue, headache, and allergic reaction. Most hypersensitivity reactions are mild or moderate and are resolved by intervention.

In patients with lymphangitis, multiple metastases, extensive lung disease such as recurrent pleural effusion, and existing lung disorder, treatment with trastuzumab may increase the risk for serious symptoms associated with infusion. If a patient with chronic lung disease is enrolled in this study, investigators should carefully consider the risks.

If Grade 4 allergic reaction, bronchospasm, or acute respiratory distress syndrome (ARDS) has occurred after the infusion of trastuzumab or pertuzumab, investigators should discontinue study treatment in the subject and enter the reason for discontinuation as intolerable toxicity.

If an IAR has occurred in a subject, investigators may decrease the infusion rate of trastuzumab or pertuzumab, or interrupt treatment with the drug. If investigators consider it clinically appropriate, they can manage the subject by supportive therapy with oxygen and medications (such as intravenous fluid, antihistamines, antipyretics, and corticosteroids). They should monitor the subject until all of the signs and symptoms due to IARs resolve completely. Before the subsequent infusions of trastuzumab or pertuzumab, antipyretics or antihistamines, or corticosteroids as needed, may be given to the subject.

Symptoms associated with infusion or lung symptoms may rarely occur 6 hours or more after the start of the infusion of trastuzumab or pertuzumab. Investigators should inform subjects of possible late-onset symptoms and instruct them to contact investigators if they have these symptoms.

If Grade 4 allergic reaction, bronchospasm, or acute respiratory distress syndrome (ARDS) occurs after the infusion of trastuzumab or pertuzumab, investigators should discontinue study treatment in the subject and enter the reason for discontinuation as intolerable toxicity. Also, if a subject cannot tolerate treatment with trastuzumab or pertuzumab due to an IAR, investigators should discontinue study treatment with trastuzumab or pertuzumab and enter the reason for discontinuation as intolerable toxicity.

6.4 Concomitant therapies

Concomitant therapies include all medications used by each subject in the period from the start of study treatment until 30 days after the last dose of study treatment or the start of posttreatment, whichever comes earlier. Investigators should collect information on all concomitant therapies and record them on the subject's CRF.

Interventions and surgical procedures during the study period that are not specified in the protocol should also be considered concomitant therapies and be recorded in the CRF. Information on concomitant drugs and therapies should include the name of the drug, the date of the intervention or procedure, and the reasons for the intervention or procedure. Medications used for treatment against cardiac toxicity (confirmed or suspected) including asymptomatic decrease in the LVEF should be reported with special attention.

It is unnecessary to collect information on therapeutic drugs other than anticancer drugs against the worsening of the primary disease. Drugs used for tests and diagnosis do not need to be recorded in the CRF unless the information is necessary to evaluate the causal relationship of adverse events.

6.4.1 Permitted concomitant therapies

If nausea, vomiting, or diarrhea occurs, effective supportive therapy should be started. Loperamide is recommended as the initial treatment against diarrhea.

If rash occurs after the administration of drugs, topical or systemic treatment may be given. Topical drugs include steroids for external use. Recommended systemic drugs include minocycline at 200 mg/day.

It is allowed to treat febrile neutropenia with a hematopoietic growth factor (granulocyte colony stimulating factor [G-CSF]) in accordance with the guideline of each study site or other guidelines (such as the ASCO guideline) although it should not be used as a primary or secondary prophylactic drug against neutropenia. The administration of G-CSF should be stopped by 48 hours before the start of the next cycle of study treatment.

It is allowed to briefly administer inhaled corticosteroids against asthma and low-dose oral corticosteroids (prednisolone equivalent dose ≤ 10 mg/day) for preventing vomiting, controlling IARs, or stimulating appetite.

For IARs after treatment with trastuzumab or pertuzumab, it is allowed to use antihistamines, nonsteroidal anti-inflammatory drugs, acetaminophen, corticosteroids, pressor drugs, epinephrine, or other drugs in accordance with the therapeutic strategy of each study site.

If zoledronic acid and denosumab are being administered for bone metastases before enrollment, they may be continued.

6.4.2 Prohibited concomitant therapies

Throughout the study treatment period, it is not allowed for each subject to undergo any kind of cancer therapy (including surgery, chemotherapy, molecular targeted drugs, antibody drugs, hormone therapy, immunotherapy, Chinese herbal medicines, and radiotherapy).

7 Information on study drugs

7.1 Pharmaceutical information on trastuzumab

1) Name: Trastuzumab Injection 150 mg

2) Ingredient:

   [Nonproprietary name] Trastuzumab (genetical recombination) (JAN)

   [Structural formula] A glycoprotein that consists of 2 light chain molecules comprising 214 amino acids each and 2 heavy chain molecules comprising 449 amino acids each

3) Strength and dosage form: An injection that contains 150 mg of trastuzumab (genetical recombination) per vial to be dissolved before use (freeze dried)

4) Storage: It should be stored at 2°C–8°C.

7.2 Pharmaceutical information on pertuzumab

1) Name: Pertuzumab Injection 420 mg

2) Ingredient:

   [Nonproprietary name] Pertuzumab (genetical recombination) (JAN)

   [Structural formula] A glycoprotein that consists of 2 light chain molecules comprising 214 amino acids each and 2 heavy chain molecules comprising 449 amino acids each

3) Strength and dosage form: An injection that contains 420 mg of pertuzumab (genetical recombination) per vial to be diluted in the solvent before use (vial)

4) Storage: It should be stored at 2°C–8°C under protection from light

7.3 Control of study drugs

The study drugs, trastuzumab and pertuzumab, will be supplied to each study site by Chugai Pharmaceutical Co., Ltd. via a delivery company, etc.

The study drug storage manager at each study site should receive, handle, store, and control the study drugs and prepare and store the records in accordance with the study drug control procedures provided by the investigator.

The study drugs should not be administered by methods other than those specified in this protocol.

7.4 Expected adverse events

With regard to adverse events after treatment with trastuzumab and pertuzumab, the events are considered to be “unknown” when the occurrence or the tendency of the occurrence (such as the

number, frequency, and conditions) cannot be expected from the current investigator's brochure of each drug, and the events are considered to be "known" when they can be expected from the investigator's brochures.

8 Endpoints, laboratory test, and evaluation schedule

The endpoints in this study are shown below. Investigators should record all information specified in this protocol.

Table 8.a Observation and test parameters

*Information also recorded in the natural history follow-up group

**Treatment**	The doses of trastuzumab and pertuzumab, the date of treatment, the date of discontinuation (interruption), the presence or absence of interruption and the reason
**Patient characteristics ***	Sex, age (at informed consent), medical history, complications, the name of clinical diagnosis, the timepoint (day, month, and year) of tumor tissue and blood sampling for central HER2-positive assessment, MSI status, registration number of GI-screen 2013-01-CRC/HER2 screening/GOZILA study, the presence or absence of other genetic abnormalities (OCP/OCA and Guardant360), the presence or absence of pretreatment and details if any
**HER2 status ***	HER2 IHC score by HER2 tests (central pathological assessment), *HER2*/*CEP17* ratio by *HER2* FISH, and copy number, presence or absence of *HER2* amplification detected by Guardant360, *HER2* copy number, the presence or absence of *RAS* gene mutation
**Physical examination**	Body height and weight
**Vital signs**	Systolic and diastolic blood pressures, pulse rate, and body temperature
**General condition***	ECOG PS
**Clinical findings and concomitant drugs/therapies**	Signs and symptoms at Grade 1 or higher, adverse events, the name of the drug (therapy), the date of administration, and the reason for concomitant treatment
**Electrocardiogram**	Resting 12-lead electrocardiogram
**LVEF**	Echocardiography, MUGA, or MRI
**Hematology**	Hemoglobin, white blood cell count, neutrophil count, and platelet count
**Biochemistry**	AST (GOT), ALT (GPT), ALP, LDH, albumin, total bilirubin, BUN, creatinine, and electrolytes (Na, K, and Cl)
**Tumor markers**	CEA and CA19-9
**Pregnancy test**	Serum test should be performed if pregnancy is suspected in urine test. Pregnancy test should be performed in premenopausal women and women within a year after the last menstruation.
**Infection test**	HIV antibody and HBs antigen
**Imaging test***	Thoracoabdominal and pelvic contrast-enhanced CT* or abdominal and pelvic contrast-enhanced MRI*, and chest X-ray, brain CT, and brain MRI as needed.<br>*If a subject is allergic or hypersensitive to contrast agents used for CT and MRI, the subject may undergo the test without contrast agents.

	If a subject discontinues study treatment for reasons other than the progression of the primary disease based on radiological assessment, the assessment in the subject should be continued until confirmed progression or the start of a new anticancer therapy.
**Biomarkers (optional)**	Tumor biopsy and blood sampling
**Posttreatment** *	Details in posttreatment (the start date and last date of treatment, the date of confirming progression, the date of discontinuation, and best overall response are observed in the natural history follow-up group)
**Survival** *	The date of death or the last date of confirming survival and the cause of death if applicable

8.1 Study calendar

Investigators should comply with the schedule shown in Table 8.b. The allowable window of visits and laboratory tests is ±3 days as long as they are performed in an appropriate sequence. The delayed schedule due to long holidays such as year end and New Year holidays should not be considered a deviation. For allowable windows for study treatment and tests, also see Table 8.c, allowable windows for study treatment and tests.

Table 8.b Study calendar

	At enrollment		Treatment per protocol<br>Each cycle (1 cycle: 21 days)	Discontinuation of study treatment†	30 days after the last dose‡	Follow-up#
Day	−28 to −1	−7 to −1	1	0 to +7 days	−3 to +7 days	Every 3 months
Informed consent	●*1					
Patient characteristics	●					
HER2 status (tumor tissue/blood)	●*2					
Body height and weight	●		○*3			
Vital signs	●		●*4	●	●	
ECOG PS	●		●*4	●	●	
Clinical findings (including adverse events) and concomitant drugs/therapies	●		●*4	●	●	
12-lead electrocardiogram	●					
LVEF (echocardiography /MUGA/MRI)	●		Every 12 weeks*5			
Study treatment			●			

Laboratory test						
Hematology and biochemistry		●	●	●	●	
Tumor markers		●	●*6	●		
Pregnancy test		○	○*7			
Infection test	●					
Biopsy and blood sampling for biomarkers	○*8		○*8	○*8		
Imaging test (thoracoabdominal and pelvic CT/MRI)	●*9 (Within 21 days before enrollment)		●*6	○*10		●*11,12
Posttreatment						●*12
Survival						●*12

●: Mandatory items; ○: Items to be performed as needed.

†The day of the discontinuation of study treatment is defined as the day when investigators have determined the discontinuation of study treatment in the subject. The evaluation at discontinuation can be performed within 7 days after the day of discontinuation. If it is impossible to perform tests and observation within the allowable range, investigators should perform them as soon as possible.

‡In subjects who have discontinued study treatment, safety follow-up should be performed by 30 days after the last dose of study treatment or the start of posttreatment, whichever comes earlier. If a subject cannot visit the study site before a new anticancer therapy starts, the subject should be followed up by phone. If safety follow-up examination is performed within 2 weeks after the examination at discontinuation, the examination at discontinuation and the safety follow-up examination may be performed at the same time. If the day of the discontinuation of study treatment is more than 30 days after the date of the end of study treatment, a safety follow-up examination is not necessary.

#It should be performed every 3 months beginning from the day of the discontinuation of study treatment (taking a month to be 4 weeks).

*1: Informed consent should be obtained before enrollment and after the HER2 status is found out.

*2: As the HER2 status for tumor tissue and blood samples, the result by central assessment should be used.

*3: The measurement is not mandatory during the study treatment period. It is unnecessary to record the result on the CRF if it is measured, unless there is a change in body weight of more than ±5 kg. If investigators consider a decrease in the body weight over time to be clinically significant, it should be recorded as an adverse event.

*4: It should be evaluated before the start of infusion in each cycle.

*5: The LVEF should be measured by echocardiography, MUGA, or MRI before infusion on the day of administration every 4 cycles (Cycles 5, 9, 13, ...).

*6: They should be measured every 6 weeks beginning from Day 1 of Cycle 1until Day 1 of Cycle 9. After Cycle 9, they should be measured every 9 weeks.

*7: The pregnancy test should be performed every 12 weeks until 7 months after discontinuation of study treatment in premenopausal women and women within a year after the last menstruation.

*8: Only in the subjects who have received explanations from investigators and have given their consent, tumor tissue samples should be collected within 3 weeks before the start of study treatment and within 2 weeks after discontinuation of study treatment and blood samples should be collected within 3 weeks (±3 days) after the start of study treatment and within 2 weeks after discontinuation of study treatment. If an investigator determines that an additional biopsy is necessary, it can be performed. Investigator should obtain a consent from the patient before the additional biopsy.

*9: It should be performed within 14 days before enrollment (inclusive of the same days of the week as the day of enrollment). The result before informed consent can be used if it is obtained within 14 days before enrollment.

*10: The imaging test is not mandatory if a subject discontinues study treatment because of the radiological result (progressive disease).

*11: The natural history follow-up group should be measured every 9 weeks after enrollment. As this is not included in evaluation of the study treatment, measurements outside the allowable window shown below are not treated as deviations.

*12: If an investigator decides that the treatment plan for an enrolled patient is best supportive care (BSC),

evaluation of images is optional in the natural history follow-up group during the period when cancer drug therapy is not administered.

Table 8.c Allowable windows for study treatment and tests

Item	Timepoint specified in the protocol	Allowable window
Laboratory test (Cycle 1)	Before infusion on Day 1	−7 days
Laboratory test (Cycle 2 and subsequent cycles)	Before infusion on Day 1 of each cycle	−3 days
LVEF evaluation	Before infusion on Day 1 every 4 cycles	−3 days
Study treatment	3 weeks after Day 1 of the previous cycle (Day 22)	±3 days
Tumor markers and imaging test (during study treatment)	Until Day 1 of Cycle 9: every 6 weeks (42 days)	±2 weeks (14 days)
	Cycle 9 and subsequent cycles: every 9 weeks (63 days)	
Imaging test (natural history follow-up group)	Every 9 weeks (63 days)	±2 weeks (14 days)
Imaging test for confirming CR and PR	At least 4 weeks (28 days) after the previous imaging test	It is not allowed to perform it 27 or fewer days after the previous imaging test

8.2 Collection, tests, and observations during the study and their procedures

Investigators should perform the following items at the timepoints specified in Section 8.1, Study calendar.

8.2.1 Informed consent

Informed consent should be obtained from a patient after an HER2-positive result is found from tumor tissue or a blood sample and before any of the study procedures is performed on the patient.

Procedures for obtaining consent are shown in Section 13.2.2.

8.2.2 Patient characteristics

・Sex and age (at informed consent)

・Medical history and complications

• The name of clinical diagnosis

Location of (one of the main) primary tumors, the presence or absence of primary tumor, the locations of metastases, the histological type of (one of the main) tissues used for central HER2-positive assessment, and the timepoint of the diagnosis of metastatic colorectal cancer

- The timepoint (day, month, and year) of tissue and blood sampling for central HER2-positive assessment

・MSI status (if they are assessed)

- Registration number of GI-screen 2013-01-CRC, HER2 screening, and GOZILA study

  This study will make secondary use of the results for genetic abnormalities in tumor tissue, HER2 status, and genetic abnormalities in blood sample obtained in each of these studies.

- Genetic abnormalities of tumor tissue analyzed with the Oncomine Cancer Research Panel (OCP)/Oncomine Comprehensive Assay (OCA) (If OCP/OCA analysis is performed with the tumor tissue used for central HER2-positive assessment, the result of this will be gathered. If OCP/OCA analysis is not performed with the tumor tissue used for central HER2-positive assessment, the results of OCP/OCA analysis with the tumor tissue sampled on the closest date to the enrollment date that is before the enrollment date.)

- Genetic abnormalities in blood samples analyzed with Guardant360

・Following information should be collected in terms of pretreatment, including whether a subject has undergone the pretreatments.

The date of surgery against the primary tumor or metastatic lesion and the details

Details in adjuvant chemotherapy, the last date of administration, and the reason for discontinuation

Details in chemotherapy, the start date and last date of administration, and the reason for discontinuation

Details in the regimens containing an anti-EGFR antibody drug (cetuximab or panitumumab), the start date and last date of administration, the date of confirming progression, the reason for discontinuation, and best overall response

Whether radiotherapy has been administered, location, the start date and last date of administration, radiation dose, etc.

Whether other therapies have been administered, details in the therapy, and treatment period

8.2.3 HER2 status

HER2 IHC score by central pathological assessment (performed separately in a clinical research), *HER2*/*CEP17* ratio by FISH and copy number, presence or absence of *HER2* amplification detected by Guardant360, *HER2* copy number, and presence or absence of *RAS* gene mutation

8.2.4 Measurement of body height, weight, and vital signs

Body height, weight, and vital signs (systolic and diastolic blood pressures, pulse rate, and body temperature) should be measured. Vital signs should be measured in the same position at all specified timepoints in each subject.

8.2.5 ECOG Performance Status

The ECOG Performance Status should be measured (see Appendix A).

8.2.6 Clinical findings (including adverse events) and concomitant drugs/therapies

Grade 1 and higher adverse events (signs and symptoms), all concomitant drugs and therapies used, the dates of administration, and the reasons for the concomitant drugs/therapies should be recorded from the start of study treatment until 30 days after the last dose of study treatment or the start of posttreatment, whichever comes earlier. For the handling of adverse events, see Section10, Report of adverse events.

8.2.7 Resting 12-lead electrocardiogram

Resting 12-lead electrocardiogram should be recorded after a subject is confirmed in a resting state. The QT interval and qualitative abnormalities including the ST-segment form, T-wave form, and the presence of U waves should be evaluated.

8.2.8 LVEF

The LVEF should be measured by echocardiography, MUGA, or cardiac MRI.

8.2.9 Status of study treatment

Investigators should record the status of study treatment (no change, interruption, or discontinuation) in each subject's CRF.

8.3 Laboratory test

Investigators should measure the parameters shown in Table 8.a in accordance with the study calendar shown in Table 8.b. If the screening examination has been performed within 7 days before Day 1 of Cycle 1, the results at the screening can be used for the laboratory test at Day 1.

8.3.1 Hematology and biochemistry

Investigators should measure the parameters shown in Table 8.a in accordance with the study calendar shown in Table 8.b and when clinically indicated. If necessary, clinically significant laboratory test should be repeated until the result returns to baseline or is stabilized clinically or another therapy is started.

8.3.2 Tumor markers

Investigators should measure the parameters shown in Table 8.a in accordance with the study calendar shown in Table 8.b.

8.3.3 Pregnancy test

For women of childbearing potential, the urine pregnancy test should be performed to confirm a negative result within 7 days before enrollment in the study. If pregnancy is suspected on the urine pregnancy test, the serum pregnancy test should be performed to confirm the result. Women not of childbearing potential include women in postmenopausal state (amenorrhea for more than a year) and women with a history of tubal ligation or hysterectomy. If applicable, investigators should clearly record it in the subject's source document.

The pregnancy test should be performed every 12 weeks (±2 weeks) starting from Day 1 of Cycle 1 until 7 months after discontinuation of study treatment in premenopausal women and women within a year after the last menstruation.

8.3.4 Infection test

HIV antibodies and HBs antigens should be measured before the enrollment of each subject. If the test has been performed within 12 months before enrollment, the result can be used.

8.3.5 Biopsy and blood sampling for biomarkers (optional)

Tumor samples will be sampled within 3 weeks after the start of study treatment, within 2 weeks after discontinuation of study treatment, and when an investigator determines that an additional biopsy is necessary, and analyzed in translational research (TR) accompanying this study. If the conditions for GI-screen 2013-01-CRC retesting are met, tumor samples will also be submitted for GI-screen 2013-01-CRC. The procedure from sampling to submission of tumor samples will be carried out according to the procedure for TR. Blood samples will also be analyzed in the GOZILA research. Accordingly, 2 × 10 mL blood samples per time will be sampled and submitted according to the procedure of the GOZILA research (optional) within 3 weeks (±3 days) after the start of study treatment and within 2 weeks after discontinuation of study treatment. The collected tumor samples and blood samples, except for those the research representative considers to be unnecessary, will be

stored under strict conditions according to the TR protocols prepared separately, in principle for 10 years after the end of the research, by the TR office (National Cancer Center Japan) Aichi Cancer Research Center Institute (tumor sample), and Division of Health Medical Computational Science, Health Intelligence Center, The Institute of Medical Science, The University of Tokyo and Guardant Health or National Cancer Center (blood sample) respectively.

8.4 Imaging test

In all subjects, the objective tumor response will be assessed according to the RECIST Guideline Version 1.1 at the following timepoints until disease progression is confirmed. Tumors in the chest, abdomen, and pelvis (when clinically indicated) should be evaluated. Thoracoabdominal and pelvic contrast-enhanced CT or MRI scan should be performed (if a subject is allergic to contrast agents, the subject may undergo the test without contrast agents).

- Within 21 days before enrollment in this study

  Response will be assessed every 6 (±2) weeks beginning from Day 1 of Cycle 1 until Day 1 of Cycle 9. After Cycle 9, it should be assessed every 9 weeks.

- If a subject discontinues study treatment for reasons other than the progression of the primary disease based on radiological assessment, the subject should be assessed according to the schedule until confirmed progression or the start of a new anticancer therapy.

- Response should be assessed every 8 (±2) weeks after the discontinuation of study treatment and in the natural history follow-up group.

Investigators should assess the objective tumor response according to the RECIST Guideline Version 1.1 (see Appendix B).

8.5 Follow-up

After the discontinuation of study treatment or enrollment in the natural history follow-up group, each subject should be followed up until the subject's death or March 2022 in terms of details in posttreatment and outcome. In the natural history follow-up group, each subject should be followed up for the start date and last date of administration, date of confirmed progression, date of discontinuation, and best overall response until the second regimen administered after enrollment. If it is impossible to directly follow up a subject for some reason such as changing hospitals or participating in another clinical study, investigators should make efforts to check the outcome if at all possible by contacting the hospital to which the subject is transferred, for example, and should record the result in the source document.

9 Data collection

9.1 Handling and retention of CRF data

Data will be managed, and CRF data will be managed and retained by Office of Clinical Research Support, National Cancer Center Hospital East. In the plan, access restriction such as inputting in and browsing the database will be set, and records will be properly controlled and kept.

9.2 Identification of source documents

Source documents in this study will be as follows:

- Medical records
- Informed consent form
- Laboratory test data
- Diagnostic imaging films, etc.

10 Reporting of adverse events

10.1 General toxicity related to trastuzumab and pertuzumab

10.1.1 Risks of hypersensitivity reactions (including anaphylaxis) associated with infusion

Infusion of monoclonal antibodies may cause symptoms associated with the infusion such as nausea, fever, diarrhea, chills, fatigue, headache, or allergic reaction. These reactions normally appear during or directly after administration. Generally (as with reactions associated with the infusion of other antibodies), IARs due to trastuzumab and pertuzumab are more frequent and severe at the initial dose, their frequency and severity decreases with time, and they completely resolve. IARs due to pertuzumab are not influenced by a history of treatment with trastuzumab or combination with trastuzumab, and the incidence and severity of these reactions is similar whether or not the subject has a history of treatment with trastuzumab or receives combination therapy with trastuzumab. Trastuzumab and pertuzumab should be administered in a healthcare environment with emergency facilities, equipment that can handle emergencies, and staff trained in monitoring of the subject's medical condition and responding to healthcare emergencies. After the initial dose of trastuzumab and pertuzumab, monitor the subject for adverse reactions for at least 60 minutes. If symptoms associated with the infusion are found, monitor the subject until signs and symptoms disappear. For a subject who develops symptoms associated with infusion, the subject's condition may be managed by decrease in the infusion speed or interruption of infusion of trastuzumab or pertuzumab, and, if an investigator decides that it is clinically appropriate, with supportive therapy with oxygen and drug therapy (for example, intravenous fluid infusion, antihistamines, antipyretics, or corticosteroids).

To prevent symptoms associated with infusion (nausea, fever, diarrhea, chills, fatigue, or headache), antipyretics, antihistamines, or corticosteroids may be administered before administration of pertuzumab or trastuzumab. If the subject is sufficient tolerant in Cycle 1, the monitoring period in subsequent cycles can be shortened to 30 minutes.

In subjects who develop dyspnea or clinically significant decreased blood pressure (assessed by the investigator), discontinue infusion of pertuzumab. In subjects who develop Grade 4 allergic reaction, bronchospasm, or ARDS, discontinue treatment.

10.1.2 Risks of decreased left ventricular ejection fraction and symptomatic cardiac failure

As both antibody drugs target the HER2 receptor, both trastuzumab and pertuzumab are associated with a risk of cardiac failure. The LVEF of all patients enrolled in clinical studies of trastuzumab and pertuzumab is measured periodically by heart ultrasound, MUGA scan, or heart MRI. Decreased

LVEF has been found in patients administered trastuzumab and pertuzumab, but the majority of patients have improved or recovered to baseline during follow-up.

It is necessary to perform LVEF measurements according to the evaluation schedule during the treatment period and after the end of administration of pertuzumab. If symptomatic LVSD occurs, or a strikingly decreased LVEF (decreased by ≥ 10% from baseline and to an absolute value < 50%), discontinue study treatment (Figure 6). Treat and monitor symptomatic LVSD according to the standard treatment method. Subjects with such conditions should be evaluated by a heart specialist, and the results of evaluation should be recorded on the CRF.

The process for management of study treatment for subjects with asymptomatic decreased LVEF (decreased by ≥ 10% from baseline and to an absolute value < 50%) is shown in Figure 6. Make a decision on whether to continue or discontinue study treatment based on two factors: the LVEF measurement and the amount of change from baseline.

10.2 General toxicity related to pertuzumab

10.2.1 Risk of toxicity related to epithelial growth factor receptor (HER1)

Pertuzumab targets HER2, but as it also forms heterodimers with other members of the HER family (for example, epithelial growth factor receptor [EGFR]), it may cause toxicity in the same way as EGFR tyrosine-kinase inhibitors (TKIs). In a Phase II single-dose study, diarrhea was found in approximately 60% of patients administered pertuzumab (and up to 90% in a study of combined administration), and in the majority of patients, it was of Grade 1 or 2 in severity. If diarrhea occurs, recommend early treatment with loperamide and also consider fluid supplementation.

Rashes have also been found during administration of EGFR-TKIs. The severity of rashes is generally mild to moderate, and some patients can be treated with standard acne medication (including topical or oral antibiotics). As of the present, in a Phase II single-dose study, a rash was found in approximately 17% of patients administered pertuzumab (and up to 40% in a study of combined administration), and overall, it was of Grade 1 or 2 in severity.

10.3 Evaluation of Adverse Events

10.3.1 Definition of Adverse Events

An adverse event (AE) is any unfavorable and unintended sign (including an abnormal laboratory value), symptom, or disease occurred in a patient administered the investigational drug, whether or not considered related to the investigational drug.

When a ≥ Grade 1 subjective symptom or objective finding has been observed since before treatment initiation (at baseline evaluation), it will be handled as an AE only when the grade of the concerned AE worsened from the baseline value.

10.3.2 Method for Recording AEs

When grading an AE, the closest definition should be selected among the definitions of Grades 0 to 5.

When a sign (including an abnormal laboratory value) or symptom is included in a diagnosis, the diagnostic term based on the CTCAE should be entered in the CRF to the extent possible instead of individual signs or symptoms.

Abnormalities of laboratory values alone should be reported as AEs only when the investigator determined that they fall into any of the following cases:

(1) Where a clinical sign or symptom is induced;

(2) Where it is judged to be clinically important;

(3) Where requiring treatment;

(4) Where requiring an additional test (excluding a retest only); or

(5) Where requiring treatment discontinuation or dose reduction for the investigational drug.

10.3.3 Items to Be Recorded for AEs

For AEs occurred, the following information should be reported in the CRF:

(1) AE term;

(2) Severity (CTCAE Grade 0 to 5 and serious/non-serious);

(3) Causal relationship with the each drug;

(4) Date of onset, date of outcome and outcome; and

(5) Presence or absence of therapeutic action.

(6) Whether the event is an ECI.

10.3.4 Assessment of the Causal Relationship

The causal relationship with the each investigational drug should be assessed on the following two categories:

"Definite," "probable," "possible" or "unassessable" → "The causal relationship cannot be ruled out."

"Unlikely" or "not related" → "The causal relationship can be ruled out."

Table 10.a Causality assessment

	Assessment	Description of assessment
A causal relationship cannot be ruled out	Definite	It is assessed that the adverse event clearly occurred/became severe due to the study treatment and there is almost no possibility that it is due to aggravation of the primary disease or other factors (concurrent conditions, other drugs/treatment, or coincidental conditions).
	Probable	It is assessed that the adverse event likely occurred/became severe due to the study treatment and it is unlikely that it is due to aggravation of the primary disease or other factors (concurrent conditions, other drugs/treatment, or coincidental conditions).
	Possible	It is assessed that, if anything, it is plausible that the adverse event occurred/became severe due to the study treatment and there is little possibility that it is due to aggravation of the primary disease or other factors (concurrent conditions, other drugs/treatment, or coincidental conditions).
	Unassessable	There is insufficient data for assessment, and assessment is not feasible
A causal relationship can be ruled out	Unlikely	It is assessed that, if anything, it is more plausible that the adverse event is due to aggravation of the primary disease or other factors (concurrent conditions, other drugs/treatment, or coincidental conditions) rather than that it occurred/became severe due to the study treatment.
	Not related	It is assessed that the adverse event is clearly due to aggravation of the primary disease or other factors (concurrent conditions, other drugs/treatment, or coincidental conditions) and there is almost no possibility that it occurred/became severe due to the study treatment.

10.3.5 Follow-up of the Clinical Course at the Onset of AEs

When an AE occurred, the investigator should immediately take an appropriate therapeutic action.

A symptom (laboratory value) should be followed up until resolution or remission is confirmed even after Safety Follow-up Visits (see the Section 10.3.6). However, the follow-up of an AE may be terminated if falling into at least any one of the following cases:

In the case of terminating the follow-up of an AE:

- Where it is not an SAE, is assessed that the causal relationship can be ruled out, and occurred at least 30 days after the day of the last dose;
- Where the principal investigator determined that the symptom is stable and is not of a medically significant concern;
- Where subsequent treatment was implemented so that the causal relationship with the investigational drug is unassessable;
- Where the clinical course cannot be followed up due to reasons such as transfer to another hospital;
- Where the patient refused to be followed up; or
- Where the patient died.

10.3.6 AE Evaluation Period

In this study, the AE evaluation period will be from the "the start day of the administration of the investigational drug" to "Safety Follow-up Visits - approximately 30 days after the last dose of trial treatment or before the initiation of a new anti-cancer treatment, whichever comes first." AEs occurring this period will be collected.

Even if it is after the AE evaluation period, AEs assessed as related to the trial treatment should be collected.

After PD is conformed, it is unnecessary to collect newly occurred AEs associated with the progression of the primary disease.

Adverse events will not be assessed in the natural history follow-up group.

10.4 Reporting of Serious Adverse Events (SAEs)

10.4.1 Definition of SAE

Of the AEs specified in the Section "10.3 Evaluation of Adverse Events", AEs falling into any of the following items are defined as "SAEs."

①An event that results in death or is life-threatening;

② An event that results in persistent or significant disability/incapacity;

③ An event that is a congenital anomaly/congenital defect; or

④ An event that requires inpatient hospitalization or prolongation of existing hospitalization

However, excluding the following events:

- Hospitalization or death due to the primary disease after PD is confirmed;
- Hospitalization or prolongation of existing hospitalization for reducing a burden to a patient who visits the hospital from a remote place;
- Hospitalization or prolongation of existing hospitalization planned beforehand;
- Hospitalization or prolongation of existing hospitalization not related to AEs; or
- Hospitalization or prolongation of existing hospitalization for no more than 24 hours only for following up of the course.

⑤ Medically important events are defined as those which possibly jeopardize the patient or require a medical or surgical intervention to prevent the outcomes listed above.

10.4.2 Events of clinical interest (ECI)

For “subjects with asymptomatic decreased LVEF that requires treatment or leads to discontinuation of study treatment,” even if the event does not meet the seriousness criteria, prepare a “SAE Report” for the event as an event of clinical interest (ECI) and report it to the coordinating committee within 24 hours in writing (by e-mail or fax) or orally (by telephone). When the initial report is made without using the “SAE Report,” a report should be promptly made using the said form (e-mail or fax). In addition, after obtaining additional information, an additional report should be made as necessary. However, when not corresponding to SAEs, it is not required to make a report to the head of the study site.

10.4.3 Reporting procedure of the Investigator to the head of the study site and the coordinating committee

1) Initial report

When an SAE (see the Section 10.4.1) occurred, the investigator should immediately take an appropriate action. The subinvestigators should promptly report it to the principal investigator.

The investigator should reported an SAE occurred in writing (e-mail* or fax) or orally (telephone) to the head of the study site and the coordinating committee within 24 hours after learning its onset. When the initial report is made without using the “SAE Report,” a report should be promptly made using the said form (e-mail or fax).

In addition, the details of the SAE should be reported in writing to the head of the study site and the coordinating committee within 5 business days after learning its onset.

* Reporting by e-mail is recommended.

2) Additional report

The principal investigator should follow up the SAE (see the Section 10.3.5). Additional information should be recorded in the "SAE Report" (additional report) and promptly sent to the head of the study site and the coordinating committee within 5 business days after learning its onset.

Contact information; The coordinating committee / Clinical Research Support Office

E-mail： triumph_core@east.ncc.go.jp

TEL： 04-7133-1111 (extension: 5200) FAX： 04-7134-6860

The investigator should provide further information upon request of the coordinating committee, the head of the study site, the institutional review board (IRB) and the investigational drug suppliers.

10.4.4 Reporting obligation of the coordinating committee and reporting procedures

The coordinating committee should determine the seriousness, causal relationship, expectedness and the necessity for reporting to the regulatory authority (Pharmaceuticals and Medical Devices Agency (PMDA)) (pursuant to "Article 80-2, Paragraph 6 of the Pharmaceutical Affairs Law (PAL)" and "Article 273 of the Enforcement Regulations of the PAL") for SAEs notified by initial and additional reports.

The details of reporting procedures should be as specified in the "Procedures for Handling of Safety Information."

1) Report to each principal investigator
The coordinating committee should promptly report the notified SAE with the details of the abovementioned determinations to each principal investigator. Each principal investigator should, as necessary, report them as soon as possible to the head of the study site in accordance with rules at each study site.

2) Report to the PMDA
When reporting to the PMDA is found necessary, the coordinating committee should handle the matter in accordance with Article 273 of the Enforcement Regulations of the PAL and relevant notifications.

3) Report to the efficacy and safety evaluation committee
When a review of an SAE by the efficacy and safety evaluation committee is judged to be necessary, the coordinating committee should report it in writing and seek its opinions on the appropriateness of the comments of the principal investigator and coordinating committee on the AE and handling (assessments of the seriousness, causal relationship and expectedness of the AE) of the AE.

4) Report to the investigational drug suppliers

The coordinating committee should promptly notify SAEs reported by the investigator to the investigational drug suppliers, Chugai Pharmaceutical Co., Ltd. In addition, the coordinating committee should, as necessary, provide information other than the “SAE Report” to the investigational drug suppliers upon their request.

10.4.5 Duties of the efficacy and safety evaluation committee

The efficacy and safety evaluation committee should examine the details of AE reports and advise, in writing, handling such as whether or not to continue the study and the necessity of protocol revisions to the coordinating committee. For their procedures, the separately stipulated “Procedures for the Efficacy and Safety Evaluation Committee” should be followed.

10.5 Collection of safety information

When safety information on the investigational drugs is received from the investigational drug suppliers, the coordinating committee should handle it in accordance with Article 273 of the Enforcement Regulations of the PAL and relevant notifications. The coordinating committee should report the safety information to each principal investigator. Each principal investigator should report it to the head of the study site as soon as possible in accordance with rules at each study site.

For the details of reporting procedures, the “Procedures for Handling of Safety Information” should be followed.

In addition, the necessity of revising the protocol and written information to patients as well as an explanation to the patients should be determined and implemented if needed.

10.6 Follow-up of pregnancy

10.6.1 Pregnancy in female subjects

For female subjects of childbearing potential, perform a pregnancy test every 12 weeks (±2 weeks) during the study or until 7 months after discontinuation of study treatment, and instruct the subject to inform the investigator immediately if pregnancy is discovered during this period. The investigator should report the pregnancy to the coordinating committee within 24 hours of learning of it, in writing (by e-mail or fax) or orally (by telephone). If the initial report is made without using a “pregnancy report form,” promptly report it using this form (by e-mail or fax). The investigator should discuss the risks of pregnancy and the possible influence on the fetus with the subject and then discontinue the study drug. Continue monitoring of the subject until the end of the pregnancy.

10.6.2 Pregnancy in female partners of male subjects

Instruct male subjects to inform the investigator immediately if their female partner becomes pregnant during the study or within 7 months after discontinuation of study treatment. The investigator should report the pregnancy to the coordinating committee within 24 hours of learning of it, in the same way as in Section 10.6.1. Try to gather and report details about the course and outcome of pregnancy in partners of male subjects exposed to the study drug. The pregnant partner must sign a consent form relating to the use and disclosure of pregnancy and health information in order to approve follow-up of the pregnancy.

When the consent form has been signed, the investigator should report the latest information relating to the course and outcome of the pregnancy on a “pregnancy report form.”

An investigator dealing with the male subject or pregnant partner may provide information about the risks of pregnancy and the possible influence on the fetus to support their decision, after information has been received through the cooperation of the physician providing treatment or the obstetrician.

11 Assessment of response and definitions of endpoints

11.1 Assessment of response

Assessments of tumor response should be made in accordance with the RECIST guideline Ver 1.1 (Appendices B).

Chest and abdomen and pelvic contrast CT or MRI (Simple CT or MRI is acceptable if the patients are allergic to contrast media.) should be performed in accordance with Table 8.b to make the assessments based on image measurements. The tests should be carried out at Week 4 (28 days) or later for confirming CR or PR for best overall response.

11.2 Definitions of endpoints

11.2.1 Confirmed objective response rate (ORR) assessed by investigator review

The ORR is the ratio of subjects with a best overall response of either CR or PR assessed by an investigator according to RECIST ver. 1.1. Confirmation of assessment of the best overall response is required.

11.2.2 Confirmed Objective Response rate (ORR) assessed by central review

The ORR is the ratio of subjects with a best overall response of either CR or PR assessed by central review according to RECIST ver. 1.1. Confirmation of assessment of the best overall response is required. However, central assessment of objective tumor response may not be performed for reasons such as cost.

11.2.3 Progression free survival (PFS)

The period will be from the day of enrollment, as the starting date of the computation, to the day when progression is determined or the day of death of any cause, whichever comes earlier.

- "Progression" will be PD based on diagnostic imaging according to overall response by irRECIST and RECIST v1.1. The day of the imaging test will be defined as the day of progression. When the clinical judgment of PD is observed and it is difficult to judge radiological PD based on diagnostic imaging due to the deterioration of patients condition, the day of the clinical judgment of PD is the day of progression.
- Surviving patients, who are not assessed as progression will be censored on the last day when no progression is confirmed on imaging (last day of PFS confirmed) (If information on progression or progression-free is obtained from medical institutions to which the patients are transferred or referred, a statement on medical information provision

documenting the rationales for a diagnosis should be received and retained. Notification only by telephone will not be acceptable).

- For patients who died without being assessed as progression, whether to consider to be an “event on the day of death” or “censoring on the last day of PFS confirmed” should be decided at the time of data review performed prior to data fixation. In principle, they should be “an “event on the day of death” unless the period from the last day of PFS confirmed to the day of death is long.
- When another treatment is given as subsequent treatment in withdrawals due to AEs or refusal, they should be censored on the start day of the subsequent treatment.
- The development of secondary cancer (asynchronous double cancer) should not be handled as an event or censoring but as PFS until other events are observed.

11.2.4 Duration of response (DoR)

This is the period from the day when the overall response according to RECIST ver. 1.1 is found to be CR or PR until the day when progression (PD based on the imaging test) is determined, or until the date of death due to any cause, whichever is earlier.

- Progression is PD (progressive disease) based on the imaging test for the overall response according to RECIST ver. 1.1, and the date of the imaging test is taken as the date of progression.
- In surviving subjects not found to have progression, data will be censored as of the last date when the specified imaging test finds that progression is not present. (If information about progression or absence of progression is received from a medical institution to which a subject is referred or transferred, receive and store the treatment information provision form containing the evidence for the diagnosis. Making contact by telephone only is not allowed.)
- For a subject who dies without being assessed as having progression, decide at the case review meeting held before data locking whether to treat the case as “an event on the date of death” or “censored as of the last date when the specified imaging test finds that progression is not present.” Unless there is a long period between the last date when the specified imaging test finds that progression is not present and the date of death, in principle, treat the case as “an event on the date of death.”

11.2.5 Time to treatment failure (TTF)

This is the period from the enrollment date until the date when progression is determined, the date of death due to any cause, or the date when the study treatment is discontinued.

- The date of discontinuation of study treatment is the date when the discontinuation of study treatment is decided. The definition of discontinuation is as in Section 6.2.1, and the date of discontinuation of study treatment is the earliest date when any of items 1) to 9) is confirmed.
- “Progression” is PD (progressive disease) based on the imaging test for the overall response according to RECIST ver. 1.1 or progression of the primary disease that cannot be found from the imaging test. If progression is determined on the basis of imaging test, the date of progression is the date of the imaging test, and if it is clinical progression, the date of progression is the date of clinical assessment.
- Data for subjects not assessed as having progression during the study treatment will be censored as of the last date when it is confirmed that there is no clinical progression (the last progression-free survival date). (If information about progression or absence of progression is received from a medical institution to which a subject is referred or transferred, receive and store the treatment information provision form containing the evidence for the diagnosis. Making contact by telephone only is not allowed.)
- For a subject who dies without being assessed as having progression during the study, decide at the case review meeting held before data locking whether to treat the case as “an event on the date of death” or “censored as of the last progression-free survival date.” Unless there is a long period between the last progression-free survival date and the date of death, in principle, treat the case as “an event on the date of death.”
- Data for subjects who are discontinued for a reason such as an adverse event or withdrawal of the subject’s consent and who receive other treatment as posttreatment will be censored on the date when posttreatment starts.
- The occurrence of secondary cancer (allochronic overlapping cancer) will not be treated as an event or censored, and the period until another event is observed will be treated as progression-free survival.

11.2.6 Disease control rate (DCR)

The DCR will be defined as the proportion of patients who achieved CR or PR, or SD for ≥ 6 weeks for best overall response according to RECIST v1.1.

11.2.7 Overall survival (OS)

The period will be from the day of enrollment, as the starting date of the computation, to the day of death of any cause.

Surviving patients should be censored on the last day of PFS confirmed (Confirmation of survival by telephone inquiry will be acceptable. However, the fact of confirming survival should be documented in medical records.).

Patients who are lost to follow up should be censored on the last day when their survival is confirmed before being lost to follow up.

11.2.8 Percentage change in sum of tumor diameters

The best percentage change in the sum of tumor diameters of measurable lesions will be shown in a waterfall plot. The best amount of change will be the maximum tumor response rate relative to baseline, or the minimum growth rate relative to baseline in subjects where no decrease is found.

The time course of the percentage change in the sum of tumor diameters from baseline will be shown in a spider plot. The origin of the horizontal axis of this plot, the time axis, will be the date of the start of study treatment.

11.2.9 Incidence of AEs

All treated patients will be defined as a denominator, and the frequency of the worst grade according to the CTCAE v4.03-JCOG during all cycles will be calculated for each AE associated with the below mentioned protocol treatment.

11.2.10 ORR in regimens including anti-EGFR antibody drugs (cetuximab or panitumumab) in pretreatment

In all regimens, including anti-EGFR antibody drugs (cetuximab or panitumumab) in pretreatment, whether in monotherapy or combination therapy, the ORR is the ratio of subjects with a best overall response of either CR or PR according to RECIST ver. 1.1.

12 Statistical matters

The Statistical Analysis Plan is summarized below. The details are presented in the separately prepared “Statistical Analysis Plan.

12.1 Handling of the patients

The handling of the patients will be decided in accordance with the criteria for patient handling. The criteria for patient handling will be developed based on a data review.

12.2 Definitions of analysis populations

Each analysis population is defined as follows:

Table 12.a Analysis populations

Abbreviation	Analysis population	Definition
All enrolled patients	As shown on the left	A population of enrolled patients excluding those with overlapping or incorrect enrollment.
FAS	Full Analysis Set	The set of all enrolled subjects, excluding subjects who met all eligibility criteria A and B but met any of the exclusion criteria (ineligible subjects)
SP	Safety Population	A population of all enrolled patients who received at least one dose of the protocol treatment

There is a supplementary explanation about FAS in Section 12.4. Figure 12.a shows the patient flow chart for this study.

Figure 12.a Patient flow chart

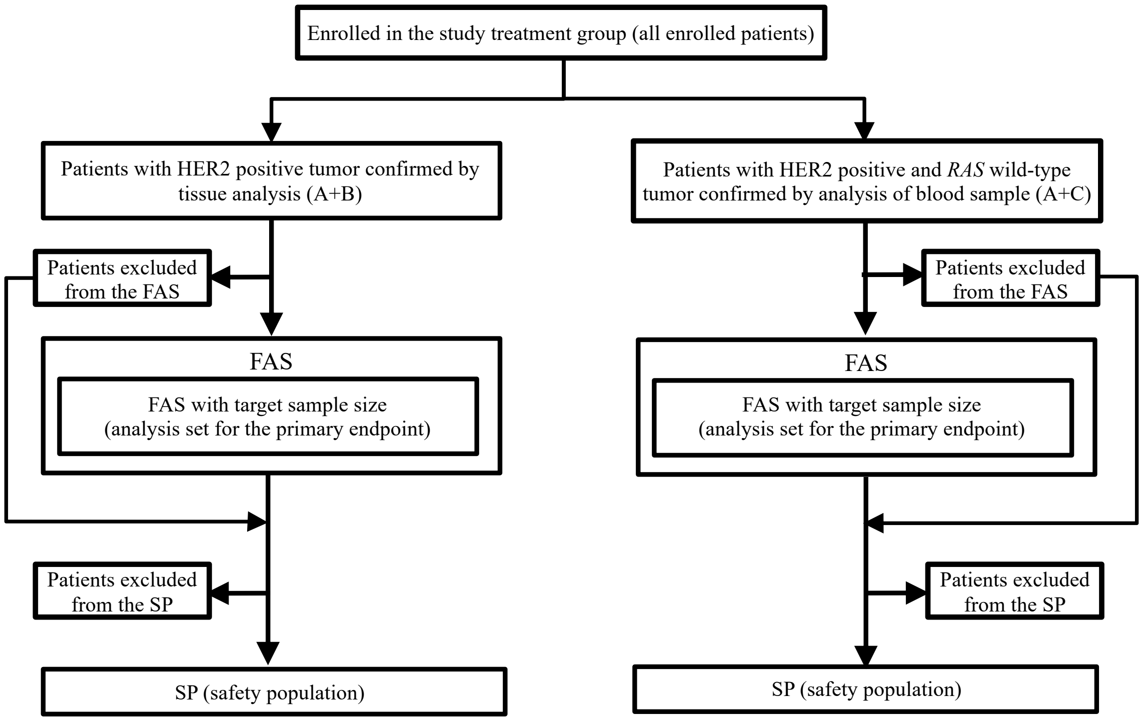

12.3 Positioning of analysis and analysis method

In this study, analysis will be performed a total of two times, in (1) and (2) below. Analysis (3) will be performed as observational research after the end of the study.

(1) Primary analysis

The primary analysis is positioned as the analysis performed after the data necessary for analysis of the primary endpoint described in Section 12.4.1 have been gathered for 18 subjects (or 25 subjects if the target sample size is increased) in the FAS. The analyzed items are listed in Section 12.9.

(2) Analysis for the clinical study report

Data that could not be analyzed as the primary analysis due to reasons such as the data gathering schedule, or items that could not be analyzed, and items for which updates are required after the primary analysis will be analyzed. The analyzed items are listed in Section 12.10.

(3) Final analysis

Analysis including follow-up data after the end of the study will be performed. As we plan to gather this follow-up data as observational research, details of the analyzed items are stated in the research plan for observational research.

12.4 Efficacy analysis

12.4.1 Primary endpoint

On the basis of the definition in Section 11.2.1, the ORR by investigators' assessment and 95% confidence intervals based on the Clopper and Pearson method will be constructed. As hypothesis tests for this study, the ORR will be calculated for the 18 subjects in the FAS with the target sample size, and if there are ≥ 4 subjects with a response based on RECIST ver. 1.1, this will be considered statistically significant. If the target sample size is changed to 25 subjects during the study, it will be considered statistically significant if there are ≥ 5 subjects with a response based on RECIST ver. 1.1. For reference, the same analysis will be performed on the FAS including extra enrolled subjects and on all enrolled subjects.

If subgroup analysis is performed, the treatment effects among subgroups will be summarized using a suitable analysis method such as Fisher's exact probability test or a logistic regression model.

The analysis set for the primary endpoint is the FAS. In this study there are the following two cohorts (see Table 2.c). The analysis sets for the primary analysis will be the FAS for each cohort. For reference, analysis will also be performed for all enrolled subjects, to evaluate efficacy in subjects excluded from the FAS.

- Patients with HER2 positive tumor confirmed by tissue analysis (A+B in Table 2.c)
- Patients with HER2 positive and *RAS* wild-type tumor confirmed by analysis of blood sample (A+C in Table 2.c)

12.4.2 Secondary endpoints

Analysis of secondary efficacy endpoints is specified as follows.

(1) ORR assessed by central review
On the basis of the definition in Section 11.2, the ORR assessed by central review and 95% confidence intervals based on the Clopper and Pearson method will be constructed. If subgroup analysis is performed, the treatment effects among subgroups will be summarized using a suitable analysis method such as Fisher's exact probability test or a logistic regression model. As stated in Section 11.2, this item will not be analyzed if central assessment is not performed.

(2) Disease control rate (DCR)
On the basis of the definition in Section 11.2, the DCR will be the ratio of subjects with a best overall response of CR, PR, or SD according to RECIST ver. 1.1. If subgroup analysis is performed, the treatment effects among subgroups will be summarized using a suitable analysis method such as Fisher's exact probability test or a logistic regression model.

(3) Progression-free survival (PFS)
Progression-free survival, progression-free survival rates at timepoints, and median progression-free survival will be estimated using the Kaplan-Meier method, 95% confidence intervals for progression-free survival rates at timepoints will be estimated using Greenwood's formula, and 95% confidence intervals for median progression-free survival will be estimated using the method of Brookmeyer and Crowley. If subgroup analysis is performed, the treatment effects among subgroups will be summarized using a suitable survival time analysis method such as the log rank test or Cox's proportional hazard model.

(4) Duration of response (DoR), time to treatment failure (TTF), overall survival (OS)
Analysis will be performed in the same way as for PFS.

(5) Percentage change in the sum of tumor diameters
The percentage change in the sum of tumor diameters will be shown in a waterfall plot. The time course of the percentage change in the sum of tumor diameters will be shown in a spider plot.

(6) ORR in regimens including anti-EGFR antibody drugs (cetuximab or panitumumab) in pretreatment
On the basis of the definition in Section 11.2, the ORR will be the ratio of subjects with a best overall response of either CR or PR according to RECIST ver. 1.1 (reported as pretreatment).

There will be four analysis sets for secondary endpoints, as in Section 12.4.1.

12.5 Safety analysis

The analysis set for safety analysis will be the SP.

As incidence of adverse events, in addition to tabulation of the frequency of adverse events, the incidence of Grade 3 or higher adverse events and the incidence of Grade 4 or higher adverse events will be calculated. If interval estimates for incidences are necessary, 95% confidence intervals will be calculated using the method of Clopper and Pearson. As necessary, information such as spider plots summarizing time courses will be reported.

12.6 Handling of data

The handling of data (for example, inclusion criteria for analysis sets) will be decided based on discussions between the coordinating committee and investigators.

12.6.1 Handling of missing values and outliers

In principle, missing values will not be imputed and analysis of outliers will not be performed. However, if it is learned before data locking that there are missing values or outliers that may have a

significant influence on the results of analysis, action for dealing with these data will be included in the statistical analysis plan.

12.6.2 Handling of additional analysis

Analysis performed after the statistical analysis plan is finalized will be positioned as additional analysis. Either the results of additional analysis will be reported in a way that makes it clear that the analysis was not planned in advance, or they will be reported by preparing an additional analysis report separately.

12.7 Target sample size

The target sample size for each of the analysis sets, "patients with HER2 positive tumor confirmed by tissue analysis" and "patients with HER2 positive and *RAS* wild-type tumor confirmed by analysis of blood sample," will be set at 18. If subjects are enrolled at a good rate after the start of the study, the target sample size will be set at 25.

12.7.1 Rationales for determination

See the section 2.6.4.3.

12.8 Interim analysis

Interim analysis will not be performed in this study.

12.9 Primary analysis

The analysis in Sections 12.4 and 12.5 will be performed as the primary analysis. The results of the primary analysis will be collected in the statistical analysis report.

12.10 Analysis performed to prepare the clinical study report

The following list of chapters will be used as a guide for the table items to list in the clinical study report, and summary indicators will be calculated as necessary. The following list is only a guide, and may change during tabulation.

<Efficacy evaluation (analysis set: all enrolled subjects and FAS)>
- 11.2 Demographic data
- 11.4.1.1 Objective response rate (ORR)
- 11.4.1.2 Disease control rate (DCR)
- 11.4.1.3 Progression-free survival (PFS)
- 11.4.1.4 Duration of response (DoR)
- 11.4.1.5 Time to treatment failure (TTF)
- 11.4.1.6 Overall survival (OS)

• 11.4.1.7 Percentage change in sum of tumor diameters
• 11.4.1.8 ORR in regimens including anti-EGFR antibody drugs (cetuximab or panitumumab) in pretreatment

<Safety evaluation (analysis set: SP)>
• 12.2.1 Incidence of adverse events (number of subjects and incidence by worst grade)
• 12.4.2 Summary statistics and time courses for laboratory test values

<Tables referred to but not included in the text of clinical study report>
• 14.3.2 Listings of deaths, other serious and significant adverse events
• 14.3.4 Laboratory value listing (each subject)

<Appendix>
• 16.2.1 Listing of discontinued subjects
• 16.2.2 Listing of subjects with significant protocol deviations
• 16.2.3 Listing of subjects excluded from the FAS
• 16.2.4a Listing of demographic data
• 16.2.4b Pretreatment history
• 16.2.4c Listing of concurrent drugs and therapies
• 16.2.5 Compliance
• 16.2.6 Listing of individual efficacy response data (tumor evaluation, OS, PFS, posttreatment, tumor markers)
• 16.2.7 Adverse event listings (each subject)

12.11 Final analysis

Analysis will be performed during the observation period after the end of the study. Details of the analysis will be specified in the observation research plan. As main analyzed items, we plan to update data for survival time endpoints such as PFS and OS.

12.12 Remarks

12.12.1 Comparison with the comparative control group

As this study is a single-arm study, if possible we would like to compare it with efficacy data for treatment other than the study treatment administered in the same population, as a past control or simultaneous control. In this study, the following two databases can be used as comparative controls of patients with HER2-positive metastatic colorectal cancer. We plan to analyze these data as comparative controls.

- The natural history follow-up group in this study
- Data extracted from the SCRUM-Japan registry

The endpoints we plan to use for comparison are the “ORR by treating physicians’ assessment (*in this study, the definition is “ORR by investigators’ assessment,” but in the above registry, the assessors are the treating physicians),” which is the primary endpoint in this study, progression-free survival, and overall survival. We plan to compare ORR and progression-free survival with the control group in the primary analysis and compare overall survival with the control group in the final analysis. We plan to use multivariate analysis methods such as trend scores proposed by the causal inference framework, but as these methods are appropriate when the sample size is sufficiently large, we will change the analysis method used depending on the status of enrollment in this study and the registry. Analysis methods will be specified in the statistical analysis plan before data locking of the primary analysis.

There are the following concerns about statistical analysis using data of the registry or natural history follow-up group, and therefore we will take measures to handle these concerns in the statistical analysis plan as far as possible.

- The possibility of bias in the treatment results due to the influence of interval censoring or missing data associated with problems such as imaging evaluation not being performed according to a specified schedule.
- The possibility that sensitivity analysis with multiple specified start dates will be necessary to align the treatment lines in the control group and study treatment group.

The procedure for extracting registry data (who will extract which data at what time) will also be specified in the statistical analysis plan.

12.12.2 Integrated analysis with external data

As stated above, in advance of this study, the HERACLES study (trastuzumab and lapatinib combination therapy) and MyPathway study (combination therapy with trastuzumab and pertuzumab) were conducted in patients with HER2-positive metastatic colorectal cancer. In this study, if possible, integrated analysis with the data gathered in these preceding studies will be performed.

As in Section 12.12.1, procedures for data management and analysis methods will be specified in the statistical analysis plan (or, if necessary, an observational research plan for performing integrated analysis).

12.12.3 Analysis sets

The analysis in Sections 12.9 to 12.11 will be performed separately for each of "patients with HER2 positive tumor confirmed by tissue analysis (A+B in Table 2.c)" and "patients with HER2 positive and *RAS* wild-type tumor confirmed by analysis of blood sample (A+C in Table 2.c)," but as necessary, analysis will also be performed on each of the cohorts A to C or on the entire integrated populations (specifically, all enrolled subjects or the all subjects in the FAS).

12.12.4 Other exploratory analysis

1) Demographic and other baseline characteristics
   These will be summarized by a suitable descriptive statistical method.
2) Tabulation of treatment compliance data
   Details of summarization of treatment courses or tabulation of data such as reasons for discontinuation will be specified in the statistical analysis plan.
3) Subgroup analysis
   Subgroup analysis of efficacy analysis will be performed for the following factors. Since this analysis is not guaranteed to have sufficient statistical power and adjustments for multiplicity will not be made, the results of subgroup analysis will be interpreted merely as exploratory results.

   - Sex (male/female)
   - Age (< 70 years/≥ 70 years)
   - ECOG PS (0 / 1)
   - Site of primary tumor (right side/left side)
   - *BRAF* status in OCP/OCA (wild-type/mutation)
   - *PIK3CA* status in OCP/OCA (wild-type/mutation)
   - *RAS* status in Guardant360 (wild-type/mutation)
   - *BRAF* status in Guardant360 (wild-type/mutation)
   - *PIK3CA* status in Guardant360 (wild-type/mutation)
   - MSI status (MSS, MSI-L/MSI-H)
   - Number of metastases to organs (≤ 1 / ≥ 2)
   - Presence or absence of primary tumor (present/absent)
   - Number of pretreatment regimens (≤ 2 / ≥ 3)

- History of angiogenic inhibitors (bevacizumab, ramucirumab, aflibercept) (present/absent)
- Response in regimens including anti-EGFR antibody drugs in pretreatment (present/absent)
- HER2 assessment in tumor tissue (positive/negative)
- HER2 IHC score in tumor tissue (0, 1+ / 2+ / 3+)
- FISH *HER2*/*CEP17* ratio in tumor tissue (high/low)
- FISH *HER2* copy number in tumor tissue (high/low)
- *HER2* amplification in blood sample (Guardant360) (++ / +++)

13 Ethical matters

13.1 Policies, laws and regulations with which the study complies

This clinical study will be conducted in compliance with the protocol, Declaration of Helsinki (http://www.med.or.jp/wma/), Article 80-2 of the PAL, "Ministerial Ordinance on Good Clinical Practice (GCP)" (Ordinance No. 28 of the Ministry of Health and Welfare dated March 27, 1997) and its revisions, and related notifications.

13.2 Informed consent

13.2.1 Explanation to the patients

The investigator should give the written information to patients approved by the IRB to the patients and orally explain the following information in detail:

1) That the study involves research
2) The purpose of the study
3) The name, title and contact address of the principal investigator or subinvestigators
4) The method of the study (including the experimental aspect of the study and inclusion/exclusion criteria for the patients)
5) Expected clinical benefits and foreseeable risks or inconveniences
6) The availability of alternative therapies and their important potential benefits and risks
7) The expected duration of the patient's participation in the study.
8) That the patient's participation in the study is voluntary and that the patient may refuse to participate or withdraw from the study, at any time, without penalty or loss of benefits to which the patient is otherwise entitled.
9) That the monitor(s), the auditor(s), and the regulatory authority(ies) will be granted direct access to the patient's source documents without violating the confidentiality of the patient, and that, by signing the informed consent form, the patient is authorizing such access.
10) If the results of the study are published, the patient's identity will remain confidential.
11) The person(s) to contact for further information regarding the study and the rights of patients, and whom to contact in the event of study-related injury.
12) The compensation and/or treatment available to the patient in the event of study-related injury
13) The approximate number of patients involved in the study
14) That the patient will be informed in a timely manner if information becomes available that may be relevant to the patient's willingness to continue participation in the study
15) The foreseeable circumstances and/or reasons under which the patient's participation in the study may be terminated.
16) The anticipated expenses, if any, to the patient for participating in the study.
17) The anticipated payment, if any, to the patient for participating in the study.
18) The patient's responsibilities
19) Type of the IRB investigating and reviewing the appropriateness of the study, matters to be

investigated and reviewed by each IRB, and other study-related matters concerning the IRB.

20) That the patient may check the written procedures for the IRB set forth in the preceding item and should request if he or she wants to do so. In addition, if the written procedures for the IRB, etc. are disclosed on a website, the fact that the address of the website is provided. If not disclosed, the fact that they are publicly available for review.

21) That data may be secondarily used.

13.2.2 Informed consent

The investigator should request the patients to participate in the study after giving an explanation on the study and sufficient amount of time to think to the patients, and confirming that they have understood well about the contents of the study. If the patients personally consent to take part in the study, each of the investigator who gave the explanation, the study collaborator who provided a supplementary explanation, and the patient who received the explanations and consented should record the dates of the explanations or consent and sign the informed consent form approved by the IRB. The investigator should retain the signed informed consent form in the medical records and hand a copy of the signed informed consent form to the patient.

In the case where information, which may affect the patients' willingness to continue participating in the study, is obtained, the investigator should promptly notify it to the patients who are in the study, verify his or her willingness to continue his or her participation in the study, and record such a fact in a document. In addition, when the principal investigator judges that a revision of the written information to patients is necessary based on the information or because of other reasons, it should be promptly revised, and approval of the IRB should be obtained. After approval of the IRB, an explanation should be provided again using the revised written information to patients, and written informed consent should be obtained again.

13.3 Protection of personal information and patient identification

Personal information and information concerning privacy such as medical data should be recognized as those requiring strict protection and careful handling under the spirit of respecting individuals' personality, and full management measures should be taken to protect privacy. We will comply with the Act for Partial Revision of the Act on the Protection of Personal Information and the Act on the Use of Numbers to Identify a Specific Individual in Administrative Procedures (Act No. 65, September 9, 2015).

13.3.1 Purposes of using personal information, items to be used and methods of use

1) Purposes of use

In this study, the patients' personal information will be used for the purpose of properly implementing monitoring, etc.

2) Items to be used

In consideration of the minimum necessity for identifying the patients and inquiries, items to be used will be as follows:

Enrollment No., patient identification (ID) No., and birth date

In other words, personal information other than the above items such as the patient's name will not be disclosed from the study sites to external parties. When study results are provided to parties other than the study sites, all information enabling to identify individuals will be anonymized and then provided.

3) Handling methods

In order to gather the personal information and medical information of subjects, investigators or study coordinators will enter it on CRFs and report it to the data center. In addition, personal information should not be exchanged by e-mail.

13.3.2 Secondary use of data

Provided approval is received from the coordinating committee and the pharmaceutical company providing the study drugs, we may make secondary use (for example, for TR, overseas approval application data for drugs or extracorporeal diagnostic agents, or meta-analysis) of the data obtained in this study, in a format where there is no link to personally identifying information.

13.3.3 Safety management responsibility system

When using personal information, safety management measures should be taken in accordance with rules at each study site to minimize the risk of information leakage. The data center should properly manage personal information in accordance with the guidelines for personal information handled by the National Cancer Center.

13.3.4 Handling of disclosure of the patients' information

The person who handles a request for the disclosure of privacy information possessed by the study by the patients if any, will be in principle, the investigator at the study site where the patient is enrolled.

13.4 Approval of the institutional review board (IRB)

13.4.1 Approval at the start of the study

When implementing this study, the principal investigator shall submit the protocol and documents specified by the GCP Ordinance such as the written information to patients to the head of the study site and receive approval of the IRB. As soon as approval of the IRB is granted, the principal investigator should send a copy of the written approval of the IRB and written information to patients (study site version) to the Clinical Study Coordinating Secretariat, and retain the original of the written approval of the IRB.

The contents of the protocol shall not be modified by the individual study sites. The protocol in common among all the study sites shall be used. When the IRB requested to modify the main text of the protocol, the principal investigator should discuss with the coordinating committee to consider its handling.

13.4.2 Approval of the IRB for the appropriateness of continuing the study

The appropriateness of continuing the study shall be reviewed by the IRB once a year. If the IRB approved the continuation of the study, the principal investigator should send a copy of the written approval of the IRB to the Clinical Study Coordinating Secretariat, and retain the original of the written approval of the IRB.

13.4.3 Changes in the contents of the protocol

When revising the protocol, written information to patients and other relevant documents, it should be made in accordance with procedures for preparation of each document.

13.4.4 Categories of changes in the contents of the protocol

In this study, changes to the protocol will be handled as revisions or amendments, and additional explanations of the protocol will be handled as notification letters or Q&As.

1) Revision

A revision refers to a change in the protocol. When the coordinating investigator determines that approval of the efficacy and safety evaluation committee is needed, a review should be requested.

2) Notification letters or Q&As

Notification letters or Q&As do not explain changes to the contents of the protocol, but are additional explanations distributed to parties related to the study by the coordinating committee in order to reduce variation in the interpretation of the text or call particular attention to something. They do not require review or approval by the response and safety evaluation committee.

13.4.5 Approval of the IRB at the time of protocol revision

When the protocol is revised during the study, the revised documents shall be approved by the IRB.

13.5 Management of conflict of interest (COI)

The conflict of interest (COI) for persons involved in this study such as the principal investigator, subinvestigators and coordinating investigators will be properly managed in accordance with the rules at the study sites. In addition, the COI for the companies has been managed in accordance with the companies' office regulations and compliance programs.

The investigational drug suppliers will not involve in any essential part of the study such as the operation of the study and the interpretation of results. However, contracts will be exchanged for the evaluation of the biomarkers (additional study) as joint studies.

13.6 Compensation

In the event of injuries attributable to the study in the patients, the study site should provide compensation in accordance with the "Procedures for compensation for injuries" even if the study site is not legally responsible.

The details of compensation in this study will be the provision of medical care, and no medical expense, medical benefit or compensation money will be paid. In principle, compensation will not prevent the execution of the patients' right to seek damages.

14 Monitoring and audits

14.1 Monitoring

Monitoring will be performed in accordance with the monitoring procedures, and central monitoring and monitoring by visiting the sites will be conducted.

Monitoring by visiting the sites will check that the clinical study is properly implemented and the reliability of data is adequately kept through direct access to source documents. After the monitoring is performed, a monitoring report should be prepared and submitted to the coordinating committee, sponsor-investigator and the head of the study site

14.2 Protocol deviations and violation

The investigator should record all acts of protocol deviations regardless of reasons. Of acts of deviations, for incompliance with the protocol for avoiding immediate hazards to the patients or other medically inevitable reasons, the principal investigator should report it in writing to the head of the study site and submit a copy of the written report sent to the head of the study site to the coordinating committee in accordance with the procedures specified at each study site.

Deviations will be classified into any of the following items after review by the coordinating committee:

1) Major deviation

“Major deviations” are defined as deviations from the provisions of the protocol that are clinically inappropriate and meet two or more of the following items:

(1) Affecting the evaluations of the endpoints in the study;

(2) Intentional or systematic

(3) Hazardous or markedly high degree of deviation

2) Deviation

Deviations excluding the item (1)

14.3 Audits

Auditors will carry out audits in accordance with the “Audit procedures” and “Audit plan” and check that the clinical study is properly implemented and the reliability of data is adequately kept through direct access to source documents

14.4 Direct access

The study site should collaborate in monitoring, audits and inspections by the IRB and regulatory authority and, as necessary, provide all study-related records such as source documents for direct

access. If the pharmaceutical company providing the study drugs makes a request for direct access, this will be handled in the same way.

15 Special note

15.1 Central assessment of the objective tumor response

In this study, central assessment of the objective tumor response will be performed by an independent organization. The investigator will submit images of the subjects for central assessment. A "procedure for central assessment of the objective tumor response" containing details of central assessment and the central assessment organization will be prepared and implemented. Decisions on whether to continue study treatment will be made by the investigator or the physician in charge of treatment on the basis of the assessment of the objective tumor response. Central assessment of the objective tumor response may not be performed for reasons such as cost.

15.2 Retention of records

15.2.1 Sponsor-investigator

Records will be stored for 5 years after the date of manufacturing and marketing approval (or, if it is learned that the data will not be attached to an application, the date 3 years after notification of this is received or the date 3 years after the discontinuation or end of this study, whichever is later). For the details, the "Procedures for retention of records" should be followed.

15.2.2 Study sites

The head of the study site and the founder of the IRB should retain essential documents, records and other relevant materials to be kept in accordance with the GCP Ordinance until the below mentioned days whichever comes later. However, when it is necessary to archive the said documents for a longer period of time than this, the archiving period and method should be discussed with the coordinating committee.

When retaining the records, the head of the study site should designate a record archiving manager:

1) Day of marketing approval for the investigational drug (When development discontinuation or the fact of not attaching study results to the approval application is notified, the day three years after the day when development discontinuation is decided or the fact of not attaching the study results to the application is notified)
2) Day three years after the discontinuation or termination of the study

15.3 Completion of the study

When the study is completed, the principal investigator should inform in writing such a fact to the head of the study site and report a summary of study results in writing.

15.4 Discontinuation at the study site

When finding that major or continuous incompliance with the GCP Ordinance or protocol by the study site interferes with the proper conduct of the study, the sponsor-investigator may prematurely terminate the study at the study site. In such a case, the sponsor-investigator should report the fact of terminating the study at the study site to the head of the study site. Also, the sponsor-investigator should report the regulatory authority in writing that the study has been discontinued.

The investigator should promptly notify such a fact to the patients, provide appropriate medical care and take other necessary measures.

15.5 Interruption of the study and discontinuation of the entire study

15.5.1 Interruption of the study

When the onset of AEs is found to be beyond the acceptable range while the study is ongoing or when the principal investigator and coordinating committee judge that the study has to be interrupted because serious ADRs or new information on the investigational drugs markedly damage the patients' safety, the principal investigator should promptly notify in writing such a fact and the details of the reason for interruption to the head of the study site. The coordinating committee should inform the regulatory authority in writing that the study has been interrupted.

15.5.2 Discontinuation of the entire study

When the principal investigator and coordinating committee judge that the entire study has to be discontinued because serious ADRs or new information on the investigational drugs markedly damage the patients' safety, the principal investigator should promptly notify in writing such a fact and the reason for discontinuation to the head of the study site. The principal investigator should inform the regulatory authority in writing that the study has been discontinued.

The investigator should promptly notify the patients, provide appropriate medical care and take other necessary measures.

16 Study organization

This is a multicenter, investigator-initiated clinical study so that the coordinating committee will be organized.

16.1 Study implementation structure

See Appendix 1.

16.2 Funding source of the study

This study was conducted with a research grant (research grant number: 16lk0201054h0001) assigned under “1-2 Promotion of Clinical Research and Investigator Initiated Trials Using Disease Registration Systems (Patient Registries)” (representative: Wataru Okamoto, Clinical Research Support Office [until March 31, 2019], Takayuki Yoshino, Department of Gastrointestinal Oncology [from April 1, 2019], National Cancer Center Hospital East) of “1. Promotion of Clinical Research and Clinical Trials Contributing to the Drug Development to Meet Patient Needs” in “2016 Project Promoting Clinical Trials for Development of New Drugs and Medical Devices ” conducted by the Japan Agency for Medical Research and Development by March 31, 2021, and from Chugai Pharmaceutical Co., Ltd. from April 1, 2021. The combination therapy drugs trastuzumab and pertuzumab were provided by Chugai Pharmaceutical Co., Ltd.

The Clinical Research Support Unit, National Cancer Center Hospital East in charge of data management, monitoring, audits and the Clinical Study Coordinating Secretariat organizes the structure through the Clinical Research Safety Assurance Support Project for Non-approved Drugs, “Clinical Trials Core Hospital Development Project, National Cancer Center Hospital East”.

17 Attribution of study results and publication of study results

Study results will belong to the National Cancer Center. The presentation or publication of study results at academic conferences or in papers will be decided by the coordinating committee upon discussion with the principal investigators and other relevant persons at the time of presentation or publication.

18 REFERENCES

1. Torre LA, Bray F, Siegel RL, Ferlay J, Lortet-Tieulent J, Jemal A. Global cancer statistics, 2012. CA Cancer J Clin 2015;65:87-108.
2. Katanoda K, Hori M, Matsuda T, et al. An updated report on the trends in cancer incidence and mortality in Japan, 1958-2013. Jpn J Clin Oncol 2015;45:390-401.
3. Japanese Society for Cancer of the Colon and Rectum (JSCCR) guidelines 2016 for the treatment of colorectal cancer. 2016.
4. Grothey A, Cutsem EV, Sobrero A, et al. Regorafenib monotherapy for previously treated metastatic colorectal cancer (CORRECT): an international, multicentre, randomised, placebo-controlled, phase 3 trial. The Lancet 2013;381:303-12.
5. Mayer RJ, Van Cutsem E, Falcone A, et al. Randomized trial of TAS-102 for refractory metastatic colorectal cancer. N Engl J Med 2015;372:1909-19.
6. Hynes NE, Stern DF. The biology of erbB-2/neu/HER-2 and its role in cancer. Biochim Biophys Acta 1994;1198:165-84.
7. Di Fiore PP, Pierce JH, Kraus MH, Segatto O, King CR, Aaronson SA. erbB-2 is a potent oncogene when overexpressed in NIH/3T3 cells. Science 1987;237:178-82.
8. Hudziak RM, Schlessinger J, Ullrich A. Increased expression of the putative growth factor receptor p185HER2 causes transformation and tumorigenesis of NIH 3T3 cells. Proc Natl Acad Sci USA 1987;84:7159-63.
9. Guy CT, Webster MA, Schaller M, Parsons TJ, Cardiff RD, Muller WJ. Expression of the neu protooncogene in the mammary epithelium of transgenic mice induces metastatic disease. Proc Natl Acad Sci USA 1992;89:10578-82.
10. Drebin JA, Link VC, Stern DF, Weinberg RA, Greene MI. Down-modulation of an oncogene protein product and reversion of the transformed phenotype by monoclonal antibodies. Cell 1985;41:695-706.
11. Drebin JA, Link VC, Greene MI. Monoclonal antibodies reactive with distinct domains of the neu oncogene-encoded p185 molecule exert synergistic anti-tumor effects in vivo. Oncogene 1988;2:273-7.
12. Drebin JA, Link VC, Greene MI. Monoclonal antibodies specific for the neu oncogene product directly mediate anti-tumor effects in vivo. Oncogene 1988;2:387-94.
13. Fendly BM, Winget M, Hudziak RM, Lipari MT, Napier MA, Ullrich A. Characterization of murine monoclonal antibodies reactive to either the human epidermal growth factor receptor or HER2/neu gene product. Cancer Res 1990;50:1550-8.
14. Yang JL, Ow KT, Russell PJ, Ham JM, Crowe PJ. Higher expression of oncoproteins

c-myc, c-erb B-2/neu, PCNA, and p53 in metastasizing colorectal cancer than in nonmetastasizing tumors. Ann Surg Oncol 1996;3:574-9.
15. Cancer Genome Atlas N. Comprehensive molecular characterization of human colon and rectal cancer. Nature 2012;487:330-7.
16. Ingold Heppner B, Behrens HM, Balschun K, et al. HER2/neu testing in primary colorectal carcinoma. Br J Cancer 2014;111:1977-84.
17. Valtorta E, Martino C, Sartore-Bianchi A, et al. Assessment of a HER2 scoring system for colorectal cancer: results from a validation study. Mod Pathol 2015;28:1481-91.
18. Richman SD, Southward K, Chambers P, et al. HER2 overexpression and amplification as a potential therapeutic target in colorectal cancer: analysis of 3256 patients enrolled in the QUASAR, FOCUS and PICCOLO colorectal cancer trials. J Pathol 2016;238:562-70.
19. Kato T OW, Hamaguchi T, Hara H, Taniguchi H, Mizukami T, Denda T, Moriwaki T, Esaki T, Yuki S, Oki E, Kajiwara T, Kudo T, Naruge D, Tamura T, Fujii S, Doi T, Ohtsu A, Shitara K, Yoshino T. The Nationwide Cancer Genome Screening Project in Japan, SCRUM-Japan GI-SCREEN: Efficient identification of cancer genome alterations in advanced colorectal cancer. J Clin Oncol 2016;34:abstr 3591.
20. Bertotti A, Migliardi G, Galimi F, et al. A molecularly annotated platform of patient-derived xenografts ("xenopatients") identifies HER2 as an effective therapeutic target in cetuximab-resistant colorectal cancer. Cancer Discov 2011;1:508-23.
21. Martin V, Landi L, Molinari F, et al. HER2 gene copy number status may influence clinical efficacy to anti-EGFR monoclonal antibodies in metastatic colorectal cancer patients. Br J Cancer 2013;108:668-75.
22. Clark JW ND, Hollis DR, Mayer RJ. Phase II trial of 5-fluororuacil (5-FU), leucovorin (LV), oxaliplatin (Ox), and trastuzamab (T) for patients with metastatic colorectal cancer (CRC) refractory to initial therapy. Proc Am Soc Clin Oncol 2003;22 (suppl):abstr 3584.
23. Ramanathan RK, Hwang JJ, Zamboni WC, et al. Low overexpression of HER-2/neu in advanced colorectal cancer limits the usefulness of trastuzumab (Herceptin) and irinotecan as therapy. A phase II trial. Cancer Invest 2004;22:858-65.
24. Sartore-Bianchi A, Trusolino L, Martino C, et al. Dual-targeted therapy with trastuzumab and lapatinib in treatment-refractory, KRAS codon 12/13 wild-type, HER2-positive metastatic colorectal cancer (HERACLES): a proof-of-concept, multicentre, open-label, phase 2 trial. Lancet Oncol 2016;17:738-46.
25. Lewis GD, Figari I, Fendly B, et al. Differential responses of human tumor cell lines

to anti-p185HER2 monoclonal antibodies. Cancer Immunol Immunother 1993;37:255-63.
26. Slamon DJ, Leyland-Jones B, Shak S, et al. Use of chemotherapy plus a monoclonal antibody against HER2 for metastatic breast cancer that overexpresses HER2. N Engl J Med 2001;344:783-92.
27. Marty M, Cognetti F, Maraninchi D, et al. Randomized phase II trial of the efficacy and safety of trastuzumab combined with docetaxel in patients with human epidermal growth factor receptor 2-positive metastatic breast cancer administered as first-line treatment: the M77001 study group. J Clin Oncol 2005;23:4265-74.
28. Romond EH, Perez EA, Bryant J, et al. Trastuzumab plus adjuvant chemotherapy for operable HER2-positive breast cancer. N Engl J Med 2005;353:1673-84.
29. Goldhirsch A, Gelber RD, Piccart-Gebhart MJ, et al. 2 years versus 1 year of adjuvant trastuzumab for HER2-positive breast cancer (HERA): an open-label, randomised controlled trial. Lancet 2013;382:1021-8.
30. Slamon D, Eiermann W, Robert N, et al. Adjuvant trastuzumab in HER2-positive breast cancer. N Engl J Med 2011;365:1273-83.
31. Bang YJ, Van Cutsem E, Feyereislova A, et al. Trastuzumab in combination with chemotherapy versus chemotherapy alone for treatment of HER2-positive advanced gastric or gastro-oesophageal junction cancer (ToGA): a phase 3, open-label, randomised controlled trial. Lancet 2010;376:687-97.
32. Scheuer W, Friess T, Burtscher H, Bossenmaier B, Endl J, Hasmann M. Strongly enhanced antitumor activity of trastuzumab and pertuzumab combination treatment on HER2-positive human xenograft tumor models. Cancer Res 2009;69:9330-6.
33. Baselga J, Gelmon KA, Verma S, et al. Phase II trial of pertuzumab and trastuzumab in patients with human epidermal growth factor receptor 2-positive metastatic breast cancer that progressed during prior trastuzumab therapy. J Clin Oncol 2010;28:1138-44.
34. Baselga J, Cortes J, Kim SB, et al. Pertuzumab plus trastuzumab plus docetaxel for metastatic breast cancer. N Engl J Med 2012;366:109-19.
35. Swain SM, Baselga J, Kim SB, et al. Pertuzumab, trastuzumab, and docetaxel in HER2-positive metastatic breast cancer. N Engl J Med 2015;372:724-34.
36. Nahta R, Hung MC, Esteva FJ. The HER-2-targeting antibodies trastuzumab and pertuzumab synergistically inhibit the survival of breast cancer cells. Cancer Res 2004;64:2343-6.
37. Leto SM, Sassi F, Catalano I, et al. Sustained Inhibition of HER3 and EGFR Is Necessary to Induce Regression of HER2-Amplified Gastrointestinal Carcinomas.

Clin Cancer Res 2015;21:5519-31.

38. Yamashita-Kashima Y, Iijima S, Yorozu K, et al. Pertuzumab in combination with trastuzumab shows significantly enhanced antitumor activity in HER2-positive human gastric cancer xenograft models. Clin Cancer Res 2011;17:5060-70.
39. Hurwitz H RK, Burris HA, Kurzrock R, Sweeney C, Meric-Bernstam F, Vanderwalde AM, Spigel DR, Bose R, Fakih Marwan, Swanton C, Guo S, Bernaards C, Beattie MS, Sommer N, Hainsworth JD. Pertuzumab + trastuzumab for HER2-amplified/overexpressed metastatic colorectal cancer (mCRC): Interim data from MyPathway. J Clin Oncol 2017;35:abstr 676.
40. Karnoub AE, Weinberg RA. Ras oncogenes: split personalities. Nat Rev Mol Cell Biol 2008;9:517-31.
41. Giantonio BJ, Catalano PJ, Meropol NJ, et al. Bevacizumab in combination with oxaliplatin, fluorouracil, and leucovorin (FOLFOX4) for previously treated metastatic colorectal cancer: results from the Eastern Cooperative Oncology Group Study E3200. J Clin Oncol 2007;25:1539-44.
42. Bennouna J, Sastre J, Arnold D, et al. Continuation of bevacizumab after first progression in metastatic colorectal cancer (ML18147): a randomised phase 3 trial. Lancet Oncol 2013;14:29-37.
43. Tabernero J, Yoshino T, Cohn AL, et al. Ramucirumab versus placebo in combination with second-line FOLFIRI in patients with metastatic colorectal carcinoma that progressed during or after first-line therapy with bevacizumab, oxaliplatin, and a fluoropyrimidine (RAISE): a randomised, double-blind, multicentre, phase 3 study. Lancet Oncol 2015;16:499-508.
44. Van Cutsem E, Tabernero J, Lakomy R, et al. Addition of aflibercept to fluorouracil, leucovorin, and irinotecan improves survival in a phase III randomized trial in patients with metastatic colorectal cancer previously treated with an oxaliplatin-based regimen. J Clin Oncol 2012;30:3499-506.
45. Watanabe T, Muro K, Ajioka Y, et al. Japanese Society for Cancer of the Colon and Rectum (JSCCR) guidelines 2016 for the treatment of colorectal cancer. Int J Clin Oncol 2017.
46. Lanman RB, Mortimer SA, Zill OA, et al. Analytical and Clinical Validation of a Digital Sequencing Panel for Quantitative, Highly Accurate Evaluation of Cell-Free Circulating Tumor DNA. PLoS One 2015;10:e0140712.
47. Liang DH, Ensor JE, Liu ZB, et al. Cell-free DNA as a molecular tool for monitoring disease progression and response to therapy in breast cancer patients. Breast Cancer Res Treat 2016;155:139-49.

48. Au HJ, Karapetis CS, O'Callaghan CJ, et al. Health-related quality of life in patients with advanced colorectal cancer treated with cetuximab: overall and KRAS-specific results of the NCIC CTG and AGITG CO.17 Trial. J Clin Oncol 2009;27:1822-8.
49. Lang I, Kohne CH, Folprecht G, et al. Quality of life analysis in patients with KRAS wild-type metastatic colorectal cancer treated first-line with cetuximab plus irinotecan, fluorouracil and leucovorin. Eur J Cancer 2013;49:439-48.

Appendix A ECOG Performance Status

Table A.1 ECOG Performance Status

PS	Contents
0	Fully active. Being able to carry on all pre-disease performance without restriction.
1	Restricted in physically strenuous activity but ambulatory and able to carry out work of a light or sedentary nature, e.g., light house work, office work.
2	Ambulatory and capable of all self-care but unable to carry out any work activities. Up and about more than 50% of waking hours.
3	Capable of only limited self-care, confined to bed or chair more than 50% of waking hours.
4	Completely disabled. Cannot carry on any self-care. Totally confined to bed or chair.

Appendix B Assessment according to RECIST ver. 1.1

Assessment will be performed as follows, according to RECIST ver. 1.1.

1. Definitions of measurable lesions

A lesion falling into any of the following definitions is measurable (measurable lesion):

1) A lesion other than malignant lymph nodes (non-nodal lesions) meeting any of the following definitions:

(1) When CT or MRI with a slice thickness of ≤ 5 mm is used, the longest diameter of the lesion must be ≥ 10 mm.

(2) When CT or MRI with a slice thickness of > 5 mm is used, the longest diameter of the lesion must be at least twice the slice thickness.

(3) Osteolytic bone metastasis with soft tissue components meeting the Item (1) or (2)

(4) Absence of other measurable non-cystic lesions and the presence of a cystic lesion meeting the Item (1) or (2)

2) A lymph node must be ≥ 15 mm in short axis when assessed by CT with a slice thickness of ≤ 5 mm.
(A lymph node with ≥ 10 mm to < 15 mm in short axis is a non-target lesion, and a lymph node with < 10 mm in short axis is not a lesion.)

3) When chest X-ray is used, the longest diameter of the lesion must be ≥ 20 mm, and the lesion must be surrounded by the lung parenchyma (not adjacent to the mediastinum or thoracic wall)

4) For clinical lesions (e.g., superficial skin lesions), the longest diameter must be ≥ 10 mm as documented by color photography including a ruler

All lesions other than those listed above are non-measurable (non-measurable lesions).

Attention should be paid that non-measurable lesions include the following lesions irrespective of imaging technique or lesion size:

- Bone lesions (excluding osteolytic lesions with measurable soft tissue components)
- Cystic lesions (excluding measurable lesions defined in the above Item 1)-(4))
- Lesions previously locally treated with radiation therapy
- Leptomeningeal disease
- Ascites, pleural or pericardial effusion
- Lymphangitic involvement of skin or lung
- Abdominal masses/abdominal organomegaly that is palpable but not measurable by imaging techniques

2. Selection and baseline documentation of target lesions

In this study, of measurable lesions identified at enrollment, 10 lesions at a maximum or 5 lesions at a maximum per organ will be selected in the order of the largest diameter (longest axis for non-nodal lesions; and short axis for malignant lymph nodes) and defined as target lesions. When selecting lesions, representative of all involved organs should be included to the extent

possible, and reproducible repeated measurements should be taken into consideration (Even if the diameter is large, a lesion difficult to measure should be avoided.).

For the selected target lesions, the sites in the order from the head to the caudal end of the body, test method, date of test, longest axis for non-nodal lesions, short axis for malignant lymph nodes, and the sum of diameters of all the target lesions (hereinafter referred to as the sum of diameters) will be recorded in the case report form (CRF).

3. Baseline documentation of non-target lesions

All lesions other than those selected as target lesions should be identified as non-target lesions, irrespective of measurability. Their sites, test method and date of test will be recorded in the CRF. Multiple non-target lesions involving the same organ may be documented as a single lesion (e.g., multiple enlarged pelvic lymph nodes or multiple liver metastases).

4. Assessment of Tumor Response

According to “8.4. Imaging test”, the target and non-target lesions will be assessed using the same test method as that used at baseline. The diameters of the target lesions, and whether the non-target lesions disappeared or progressed will be recorded in the CRF.

5. Response criteria for target lesions

- Complete Response (CR)

  At least a 30% decrease in the sum of diameters of target lesions, taking as reference the baseline sum diameters.

- Partial Response (PR)

  At least a 30% decrease in the sum of diameters of target lesions, taking as reference the baseline sum diameters.

- Progressive Disease (PD)

  At least a 20% increase in the sum of diameters of target lesions, taking as reference the smallest sum on study (this includes the baseline sum if that is the smallest on study). The sum must also demonstrate an absolute increase of at least 5 mm.

- Stable Disease (SD)

  Neither sufficient shrinkage to qualify for PR nor sufficient increase to qualify for PD, taking as reference the smallest sum diameters while on study.

- Not all Evaluated

  No test can be performed for some reason, or lesions cannot be assessed as any of CR, PR, PD or SD.

  Percent reduction in the sum of diameters = (Pre-treatment sum of diameters - sum of diameters at assessment) /(Pre-treatment sum of diameters) × 100%

Percent increase in the sum of diameters = (Sum of diameters at assessment – smallest sum of diameters) /(Smallest sum of diameters) × 100%

* All target lesions should have their actual measurements recorded whenever they are measurable (e.g., even when < 5 mm). However, if target lesions are reported as "too small to measure," irrespective of CT slice thickness, the diameter will be recorded as 0 mm if the lesion has likely disappeared, or as 5 mm if the lesion is faintly seen but too small to measure.

* When the percent reduction meets the criteria for PR, and the percent increase meets the criteria for PD at the same time, PD should be determined.

* For lesions that split during treatment, the fragmented portions should be added together to calculate the sum of diameters.

* If multiple lesions have coalesced during treatment and they are no longer separable, the diameter of the coalesced lesion should be added to calculate the sum of diameters. If lesions coalesce, and a plane between them may be maintained, the diameter of each individual lesion should be added to calculate the sum of diameters.

6. Response criteria for non-target lesions

- Complete Response (CR):

  Disappearance of all non-target lesions and all lymph nodes must be non-pathological in size (< 10 mm short axis).

- Non-CR/non-PD

  Persistence of one or more non-target lesion(s) (including persistence of non-target lymph nodes measuring ≥ 10 mm in short axis)

- Progressive Disease (PD)

  "Unequivocal progression" of existing non-target lesions (including recurrence).

  When the patient also has measurable disease: In this setting, to achieve "unequivocal progression" on the basis of the non-target disease, there must be an overall level of substantial worsening in non-target disease such that, even in presence of SD or PR in target disease, the overall tumor burden has increased sufficiently to merit discontinuation of therapy. When the response of target lesions is SD or PR, an increase in the tumor burden of a non-target lesion much higher than a decrease in the tumor burden is considered to be "unequivocal progression." If not, it is assessed as Non-CR/non-PD.

  When the patient has only non-measurable disease: As an idea, "unequivocal progression" is an increase in a non-target lesion that is determined to clearly exceed the tumor burden representing a 20% increase in the diameter and an additional 73% increase in volume.

- Not all Evaluated

  No test can be performed for some reason or when lesions cannot be assessed as any of CR, Non-CR/non-PD or PD.

7. Presence or absence of new lesions

When a lesion, which was not present at baseline, is identified after the start of treatment, it should be considered to be the appearance of a “new lesion.” The finding of a “new lesion” should be unequivocal: Not attributable to any difference from baseline in scanning technique, change in imaging modality or findings thought to represent something other than tumor. For example, necrosis of a liver metastatic lesion appeared as a new cystic lesion should not be handled as a new lesion. A lesion identified on a follow-up examination in an anatomical location that was not required to be scanned at baseline (pre-enrollment evaluation) is handled as a new lesion.

If a lesion disappears and reappears at a subsequent time point, it should continue to be measured. However, the patient’s response at the point in time when the lesion reappears will depend upon the status of his/her other lesions. For example, if the patient’s tumor had reached a CR status and the lesion reappeared, then the patient would be considered PD at the time of reappearance. In contrast, if the tumor status was a PR or SD and one lesion which had disappeared then reappears, its maximal diameter should be added to the sum of the remaining lesions for a calculated response: in other words, the reappearance of an apparently “disappeared” single lesion amongst many which remain is not in itself enough to qualify for PD: that requires the sum of all lesions to meet the PD criteria. The rationale for such a categorization is based upon the realization that most lesions do not actually “disappear” but are not visualized because they are beyond the resolving power of the imaging modality employed.

If a lesion may be new but equivocal, the lesion should not be recorded as a new lesion, but imaging examination should be repeated after a clinically appropriate interval. If a repeat imaging test confirms that it is a new lesion, then a new lesion should be declared using the date of the imaging test when it is confirmed to be a new lesion.

Negative FDG-PET at baseline, with a positive FDG-PET at follow-up is a sign of PD for overall response based on a new lesion (a positive FDG-PET scan lesion means one which is FDG avid with an uptake greater than twice that of the surrounding tissue on the attenuation corrected image). No FDG-PET at baseline and a positive FDG-PET at follow-up: If the positive FDG-PET at follow-up corresponds to a new site of disease confirmed by CT of MRI, and if the positive FDG-PET confirms a lesion not identified on CT or MRI at baseline, this is a new lesion.

8. Overall Response

Overall response will be assessed based on a combination of the response of target lesions, response of non-target lesions and the appearance of a new lesion in accordance with Table B.1. below.

表 B.1 Overall response at each time point: Patients with target lesions

Target lesions	Non-target lesions	New lesions	Overall response
CR	CR	No	CR
CR	Non-CR/non-PD	No	PR
CR	Not evaluated	No	PR
PR	Non-PD or not all evaluated	No	PR
SD	Non-PD or not all evaluated	No	SD
Not all evaluated	Non-PD	No	NE
PD	Any	Yes or No	PD
Any	PD	Yes or No	PD
Any	Any	Yes	PD

9. Best Overall Response

Overall response will be considered "good" in the order of CR > PR > SD > PD > NE. Best overall response will be assessed based on the overall response during the entire study period in accordance with the criteria specified below.

- Complete Response (CR):

The CR status for overall response is maintained in at least two consecutive tests performed at intervals of 4 weeks (28 days) or more. The day of confirming CR for overall response in the second test is considered to be the "day of confirming CR."

- Partial Response (PR):

The PR or higher status for overall response (CR or PR) is maintained in at least two consecutive tests performed at intervals of 4 weeks (28 days) or more. The day of confirming the PR or higher status for overall response in the second test and PR is confirmed for best overall response is considered to be the "day of confirming PR."

- Stable Disease (SD):

No CR or PR is achieved for best overall response, but PD is not assessed for overall response before 6 weeks after the start of treatment, and SD or higher is assessed in at least one test for overall response.

- Progressive Disease (PD):

No CR, PR or SD is assessed for best overall response and but is confirmed to be PD for overall response.

- Not Evaluable (NE):

All overall responses were NE.

If images cannot be assessed due to obvious progression of disease or death before the initial assessment of response, the result will be taken to be PD. If images cannot be assessed because of a reason such as discontinuation due to an adverse event or withdrawal of consent by the research participant before the initial assessment of response, the result will be taken to be NE.

表 B.2 Best overall response

Overall response First time point	Overall response Subsequent time point	BEST overall response
CR	CR	CR
CR	PR	SD, PD, or PR[a]
CR	SD	SD provided minimum criteria for SD duration met, otherwise, PD[b]
CR	PD	SD provided minimum criteria for SD duration met, otherwise, PD[b]

CR	NE	SD provided minimum criteria for SD duration met, otherwise NE[b]
PR	CR	PR
PR	PR	PR
PR	SD	SD
PR	PD	SD provided minimum criteria for SD duration met, otherwise, PD[b]
PR	NE	SD provided minimum criteria for SD duration met, otherwise NE[b]
NE	NE	NE

a. If a CR is truly met at first time point, then any disease seen at a subsequent time point, even disease meeting PR criteria relative to baseline, makes the disease PD at that point (since disease must have reappeared after CR). Best response would depend on whether minimum duration for SD was met. However, sometimes 'CR' may be claimed when subsequent scans suggest small lesions were likely still present and in fact the patient had PR, not CR at the first time point. Under these circumstances, the original CR should be changed to PR and the best response is PR.

b. In this study, the minimum interval for SD is 6 weeks.

Clinical Research Support Office (OCRS)
National Cancer Center Hospital East

EPOC1602
Multicenter Phase II study in patients with HER2-positive metastatic colorectal cancer
TRIUMPH study

STATISTICAL ANALYSIS PLAN
ver3.1

Confidentiality Statement

This Statistical Analysis Plan is confidential information and will be provided to the Principal Investigator, Research Office, physicians involved in the research, investigators, collaborators (including external outsourced contractors such as SMO), and medical institutions that participate in this study, as well as to the Ethics Review Committee (or its equivalent organization) and the Efficacy Safety Evaluation Committee. This Statistical Analysis Plan will not be disclosed to any third party or used for any purpose other than this study without the written consent of the Principal Investigator.

Signatures

Role	Signature/Date
Clinical trial coordinating physician (representative)	
OCRS manager	
Chief of OCRS statistics operations	
Chief of statistical analysis	

Version history

Version	Date	Author(s)	Note
1.0	Dec 17, 2018	Shogo Nomura<br>Masashi Wakabayashi<br>Gakuto Ogawa	First edition
2.0	Jul 1, 2019	Shogo Nomura<br>Masashi Wakabayashi	Addition to 1.1 and 11.1
2.1	Aug 9, 2019	Shogo Nomura<br>Masashi Wakabayashi	Addition to 11.1
3.0	Feb 20, 2020	Shogo Nomura<br>Masashi Wakabayashi<br>Kei Ikeno	Addition and revision to 9.2
3.1	Jul, 11, 2020	Shogo Nomura<br>Masashi Wakabayashi<br>Kei Ikeno	Addition and revision to 11.2

1. Objective of this document

This Statistical Analysis Plan (SAP) seeks to establish the data analysis plan for the EPOC1602 clinical trial supported by the Clinical Research Support Office (OCRS) at the National Cancer Center Hospital.

1.1. Statistical Analysis Plan and its Corresponding Clinical Trial Implementation Plan

Statistical Analysis Plan	Corresponding Clinical Trial Implementation Plan
version 3.1	version 6.0

(Hereafter, the clinical trial implementation plan is referred to as the protocol)

【additional notes in ver2.0】

The main analysis will be conducted according to the latest version of the Research Implementation Plan version 7.0.

1.2. Applied manuals, etc.

- ❑ SOP of STAT group (NCCHE-OCRS-SOP-Stat-001）
- ❑ STAT group manual (NCCHE-OCRS-SOP-Stat-001-1−5）
- ❑ SOP of DM group (NCCHE-OCRS-DM-001, NCCHE-OCRS-DM-001-1−13）

1.3. Position of each analysis

Quoted from protocol 12.3

In this clinical trial, ① and ② below will be performed twice. Analysis ③ will be performed in an observational study following the clinical trial.

① Main analysis
This analysis will be performed after the data necessary to analyze the primary endpoint described in 12.4.1 has been collected from 18 cases of FAS (25 cases if the sample size is revised upward). The analysis items are described in Section 12.9.

② Analysis for the preparation of a Clinical Study Report
This analyzes the items that are not or cannot be analyzed by the main analysis owing to data collection schedule, etc., as well as items that need to be updated from the main analysis. The analysis items are described in Section 12.10.

③ Final analysis
This includes the analysis of tracking data after the clinical trial. The tracking data are to be collected as an observational study, and details of the analysis items are described in the protocol of the observational study.

1.3.1. Case review meeting

A case review meeting will be held once this SAP is established. The decisions made at the meeting will be recorded in minutes, which will be maintained by the DM group.

2. Objective of this study

The objective of this study is to “assess the efficacy and safety of simultaneous combination therapy with trastuzumab and pertuzumab in patients with *HER2*-positive unresectable, advanced/recurrent colorectal cancer that is refractory or intolerant to standard chemotherapy.” If this study statistically shows that the treatment results exceed the threshold of the confirmed objective response rate (ORR), which is the primary endpoint assessed by investigator review, and that secondary endpoints indicate improvement in the efficacy and safety, then simultaneous combination therapy with trastuzumab and pertuzumab will be considered as a promising treatment, and the next phase of the clinical trial will be discussed.

3. Study design

3.1. Overview

Quoted from protocol 2.6

This study is an unblinded, single-arm, multicenter phase II clinical trial that assesses the efficacy and safety of simultaneous combination therapy with trastuzumab and pertuzumab in patients with *HER2*-positive unresectable advanced/recurrent colorectal cancer that is refractory or intolerant to standard chemotherapy. The efficacy will be assessed by ORR as the main endpoint. In accordance with the SAP, the efficacy of the clinical trial drugs will be compared and analyzed using data from patients with *HER2*-positive unresectable advanced/recurrent colorectal cancer in the natural history group and SCRUM-Japan Registry as control. In addition, data from the SCRUM-Japan Registry will be collected in clinical studies that comply with the “Ethical Guidelines for Medical and Health Research Involving Human Subjects” (This study is not based on the GCP Ministerial Ordinance). If promising results are obtained in this study, we will consult with the clinical trial drug provider on the application for regulatory approval of trastuzumab and pertuzumab, which have not been approved domestically for use in *HER2*-positive unresectable advanced/recurrent colorectal cancer—a rare disease.

3.1.1. Endpoints

Quoted from protocol 2.6.2

This clinical trial has the following endpoints:

Primary endpoint:

Confirmed ORR assessed by investigator review

The following two target groups will be analyzed (Analysis target groups in the main analysis are specified in Chapter 12):

- Patients with *HER2*-positive tumor confirmed by tissue analysis (A + B in Table 2b)
- Patients with *HER2*-positive and *RAS* wild-type tumor confirmed by blood sample analysis (A + C in Table 2b)

Secondary endpoints:

The following items will be assessed for each of "patients with *HER2*-positive tumor confirmed by tissue analysis (A + B in Table 2b)" and "patients with *HER2*-positive and *RAS* wild-type tumor confirmed by blood sample analysis (A + C in Table 2b)."

- Progression-free survival (PFS)
- Duration of response (DoR)
- Time to treatment failure (TTF)
- Disease control rate (DCR)
- Overall survival (OS)
- Confirmed ORR assessed by central review*
- Rate of change in the sum of the diameters of tumors
- The occurrence rate of adverse events
- The ORR of regimens with anti-EGFR antibody drug (cetuximab or panitumumab) in previous treatment

3.2. Analysis target groups

The analysis target was determined on the basis of the guideline of the Implementation Plan. All analyses will be performed on each of the “cohort with *HER2*-positive tumor confirmed by tissue analysis” and the “cohort with *HER2*-positive and *RAS* wild-type tumor confirmed by blood sample analysis.”

Quoted from protocol 12.2

Each analysis target group is defined below:

Table 12a. Analysis target groups

Abbreviation	Analysis target group	Definition
All enrolled cases	As shown in the left	The group of subjects enrolled in this clinical trial excluding duplicate and false enrollments
FAS	Full Analysis Set (Largest analysis target group)	The group of all enrolled subjects that meet eligibility criteria A and B, excluding the ones that meet any of the exclusion criteria (ineligible cases)
SP	Safety Population (Safety analysis target group)	Of all enrolled subjects, the group that received this clinical trial treatment at least once

A supplementary explanation on FAS has been added to Section 12.4. The patient flowchart of this clinical trial is shown below.

Figure. Patient flowchart

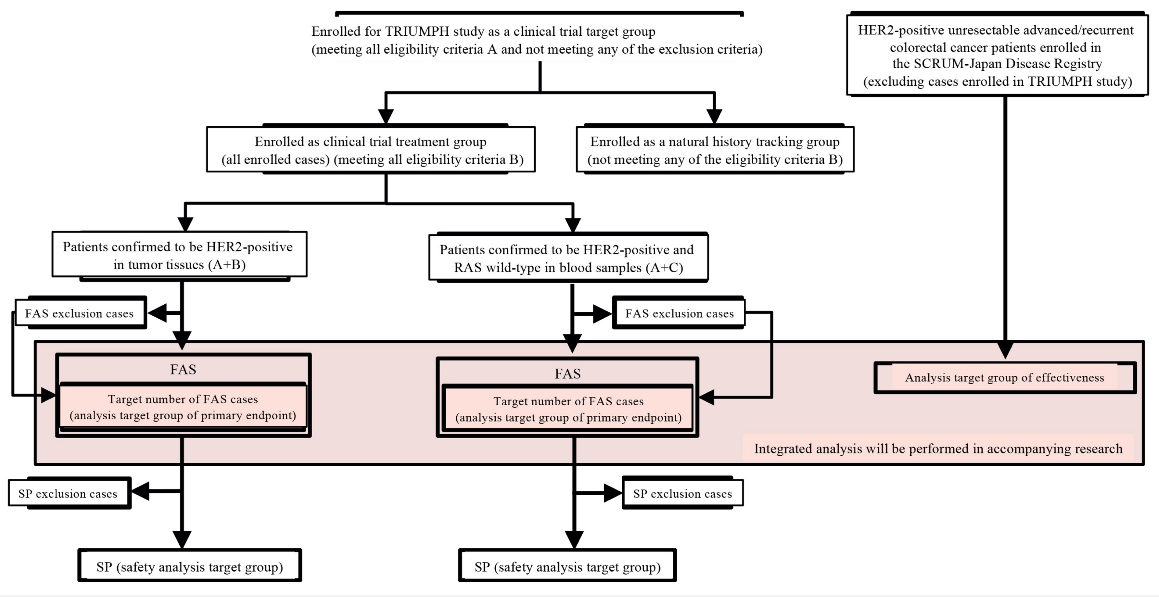

※ The analysis target groups are defined as mentioned below and applied in Chapter 4 and later:

- All enrolled cases: Of the subjects enrolled in this clinical trial, all groups enrolled as the clinical trial treatment group
- All enrolled cases (tissue): Of the subjects enrolled in this clinical trial, the cohort of patients with *HER2*-positive tumor confirmed by tissue analysis
- All enrolled cases (liquid): Of the subjects enrolled in this clinical trial, the cohort of patients with *HER2*-positive and *RAS* wild-type tumor confirmed by blood sample analysis
- All enrolled cases (natural history group): Of the subjects enrolled in this clinical trial, all groups enrolled as natural history group
- Full analysis set (FAS; tissue): Of FAS, the cohort of patients with *HER2*-positive tumor confirmed by tissue analysis
- FAS (tissue-primary): Of FAS (tissue), 25 cases from the earliest enrollment
- FAS (liquid): Of FAS, the cohort of patients with *HER2*-positive and *RAS* wild-type tumor confirmed by blood sample analysis
- FAS (liquid-primary): Of FAS (liquid), 25 cases from the earliest enrollment
- SP (tissue): Of SP, the cohort of patients with *HER2*-positive tumor confirmed by tissue analysis
- SP (liquid): Of SP, the cohort of patients with *HER2*-positive and *RAS* wild-type tumor confirmed by blood sample analysis

3.3. Allocation method of the administration group

Not applicable as the administration group is not randomly assigned in this study.

3.4. Sample size

Quoted from protocol 2.6.4.3

For each of analysis groups “patients with *HER2*-positive tumor confirmed by tissue analysis (A + B in Table 2c)” and “patients with *HER2*-positive and *RAS* wild-type tumor confirmed by blood sample analysis (A + C in Table 2c),” the threshold of ORR, which is the primary endpoint, is set to 5%, the expected value to 30%, the one-sided significance level to 2.5%, the power of detection to 80%, and the sample size to 18 (*a setting based on an the accurate method with binomial distribution). The observation of a response in 4 or more of the 18 cases will be considered as a statistically significant result. If any of the analysis target groups shows a rapid accumulation of cases, whether the power of detection can be revised upward to 90% (revising sample size upward to 25) will be discussed during the clinical trial.

Quoted from protocol 2.6.4.4

This is a single-arm clinical trial. Thus, its results will be biased if efficacy data (whether a response is observed, in particular) of some cases are shared among researchers, and the selection of enrolled cases becomes arbitrary based on its review. Because of this, the upward revision of the sample size will be considered only on the basis of the number of enrollments that are monitored regularly. If 13 cases are enrolled one year after the enrollment of the first case in each analysis target group (or if the enrollment of 13 cases in the near future is highly probable), then an upward revision of the number of subjects will be discussed by the researchers.

If the power of detection is revised upward to 85% (sample size is revised upward to 19) owing to the rapid accumulation of cases, the observation of a response in 4 or more of the 19 cases will be considered as a statistically significant result. Similarly, if the power of detection is revised upward to 90% (sample size is revised upward to 25), the observation of a response in 5 or more of the 25 cases will be considered as a statistically significant result.

The enrollment prospects of the two analysis target groups in this clinical trial (A + B or A + C in Table 2c) are uncertain, and either group may reach the sample size sooner than the other. Once either group reaches the sample size, B (when A + B reaches the sample size first) or C (when A + C reaches the sample size first) will not be enrolled in the clinical trial treatment group. However, because enrollment of A continues, one of the analysis target groups will have an excess number of subjects at the time of the main analysis. In this case, the main analysis will be performed on the sample size that is set based on statistical grounds by excluding the excess enrollment cases, but the analysis including these excess cases will also be performed as a reference. Although excess enrollment of subjects will be avoided, both analysis target groups of this study contain A, and the enrollment in either of the analysis groups cannot be terminated. Therefore, we can terminate the enrollment of only non-overlapping parts (B or C) of the analysis target groups.

The first case was enrolled on January 24, 2018; since then, 13 cases of A + B and 12 cases of A + C have been enrolled in approximately 6 months (as of July 27, 2018). Therefore, more than 13 cases will likely be enrolled in each group of A + B and A + C within one year after the enrollment of the first case of each analysis target group. Thus, the sample size was revised upward from 18 to 25. In addition, the agreement on the upward revision of the subject

size is found in the information below:

Saved file path：https://ncc123.sharepoint.com/sites/cns-portal/cts/doclib19/EPOC1602_TRIUMPH/20_会議資料_情報配信/20180808_上方修正の相談

Saved file name：20180808_TRIUMPH_minutes(上方修正等について)_fix.pdf

In addition, listed below are the boundaries by the number of enrolled cases when the data are reviewed with an ORR threshold of 5%, expected value of 30%, and one-sided significance level of 2.5%:

Number of enrolled cases	Number of response cases that are boundaries	ORR corresponding to the boundary (95% CI*)
17 cases	4 cases	23.5% (6.8–49.9%)
18 cases	4 cases	22.2% (6.4–47.6%)
19 cases	4 cases	21.1% (6.1–45.6%)
20 cases	4 cases	20.0% (5.7–43.7%)
21 cases	4 cases	19.0% (5.4–41.9%)
22 cases	5 cases	22.7% (7.8–45.4%)
23 cases	5 cases	21.7% (7.5–43.7%)
24 cases	5 cases	20.8% (7.1–42.2%)
25 cases	5 cases	20.0% (6.8–40.7%)
26 cases	5 cases	19.2% (6.6–39.4%)
27 cases	5 cases	18.5% (6.3–38.1%)
28 cases	5 cases	17.9% (6.1–36.9%)
29 cases	5 cases	17.2% (5.8–35.8%)
30 cases	5 cases	16.7% (5.6–34.7%)

*The confidence interval was calculated using the Clopper–Pearson method.

【Added in ver3.0】

This study set two analysis target groups (A + B or A + C in Table 2c), and a one-tailed hypothesis test was performed using a 2.5% level of significance for each group without considering the adjustment of test multiplicity associated with the two hypothesis tests. The family-wise error rate is 5% at most (one side) for the two hypothesis tests for each group.

4. Analysis plan

※ The analysis items listed in 4.3 to 4.9 will be analyzed separately for “patients with *HER2*-positive tumor confirmed by tissue analysis” and “patients with *HER2*-positive and *RAS*-wild-type tumor confirmed by blood sample analysis.” The items listed in 4.3 to 4.5 and 4.9 will also be collected and analyzed in the natural history group. However, items other than “4.3. Collection of background factors” will be listed in a table.

4.1. Technical items

Data will be analyzed and collected using the following versions of software:

- ❑ SAS 9.4 (SAS Institute Inc., Cary, NC, USA) 64bit version
- ❑ Microsoft Excel 2010, 2013

4.2. Collection of enrollment status

Subjects: All enrolled cases

4.2.1. Summary of test implementation status

- ❑ Number of enrolled cases per institution
- ❑ Number of cases for each analysis target group

4.3. Collection of background factors

Subjects: FAS (tissue), FAS (liquid), all enrolled cases (tissue), all enrolled cases (liquid), all enrolled cases (natural history group)

The following items on background factors at the time of enrollment and data on previous treatments will be collected. If the collected items are continuous variables, general summary statistics (mean, median, minimum, maximum, first quartile, third quartile, etc.) will be calculated.

4.3.1. Background factors at the time of enrollment

	N(%)		N(%)
Age		**WBC**	
median	XX	median	XX
Q1－Q3	XX- XX	Q1－Q3	XX- XX
min－max	XX- XX	min－max	XX- XX
Under 70	XX(XX.X)	**LDH**	
70 or older	XX(XX.X)	median	XX
Gender		Q1－Q3	XX- XX
Male	XX(XX.X)	min－max	XX- XX
Female	XX(XX.X)	**ALP**	
**Performance status**		median	XX
0	XX(XX.X)	Q1－Q3	XX- XX
1	XX(XX.X)	min－max	XX- XX
Primary lesion		**CEA**	
Cecum	XX(XX.X)	median	XX
Ascending colon	XX(XX.X)	Q1－Q3	XX- XX
Transverse colon	XX(XX.X)	min－max	XX- XX
Descending colon	XX(XX.X)	**CA19-9**	
Sigmoid colon	XX(XX.X)	median	XX
Rectal sigmoid (Rs)	XX(XX.X)	Q1－Q3	XX- XX
Rectum (Ra/Rb)	XX(XX.X)	min－max	XX- XX
Primary lesion site		Serum HER2	
Right	XX(XX.X)	median	XX
Left	XX(XX.X)	Q1－Q3	XX- XX
Tissue type		min－max	XX- XX
pap	XX(XX.X)		
tub1	XX(XX.X)		
tub2	XX(XX.X)		
por1	XX(XX.X)		
por2	XX(XX.X)		
muc	XX(XX.X)		
sig	XX(XX.X)		
Other	XX(XX.X)		

	N(%)		N(%)
**HER2 status**		Metastatic organ (Lung)	
IHC 3+	XX(XX.X)	No	XX(XX.X)
DISH positive	XX(XX.X)	Yes	XX(XX.X)
**BRAF status(OCP/OCA)**		Metastatic organ (Peritoneum)	
wild	XX(XX.X)	No	XX(XX.X)
mutant	XX(XX.X)	Yes	XX(XX.X)
Cannot be analyzed	XX(XX.X)	Metastatic organ (Lymph node)	
**PIK3CA status(OCP/OCA)**		No	XX(XX.X)
wild	XX(XX.X)	Yes	XX(XX.X)
mutant	XX(XX.X)	Metastatic organ (Other)	
Cannot be analyzed	XX(XX.X)	No	XX(XX.X)
**KRAS status(Guardant360)**		Yes	XX(XX.X)
wild	XX(XX.X)	Number of metastatic organ	
mutant	XX(XX.X)	0	XX(XX.X)
Cannot be analyzed	XX(XX.X)	1	XX(XX.X)
**NRAS status(Guardant360)**		2 or more	XX(XX.X)
wild	XX(XX.X)	Primary lesion	
mutant	XX(XX.X)	No	XX(XX.X)
Cannot be analyzed	XX(XX.X)	Yes	XX(XX.X)
**BRAF status(Guardant360)**		HER2-IHC score (tumor tissue)	
wild	XX(XX.X)	0	XX(XX.X)
mutant	XX(XX.X)	1+	XX(XX.X)
Cannot be analyzed	XX(XX.X)	2+	XX(XX.X)
**PIK3CA status(Guardant360)**		3+	XX(XX.X)
wild	XX(XX.X)	HER2/CEP17 ratio (tumor tissue)	
mutant	XX(XX.X)	median	XX
Cannot be analyzed	XX(XX.X)	Q1 – Q3	XX– XX
**MSI status**		min – max	XX– XX
MSS	XX(XX.X)	HER2 copy number (tumor tissue)	
MSI-L	XX(XX.X)	median	XX
MSI-H	XX(XX.X)	Q1 – Q3	XX– XX
Untested	XX(XX.X)	min – max	XX– XX
Metastatic organ (Liver)		HER2 amplification (blood sample)	
No	XX(XX.X)	++	XX(XX.X)
Yes	XX(XX.X)	+++	XX(XX.X)

4.3.2. Treatment history

	N(%)
Surgery	
No	XX(XX.X)
Yes	XX(XX.X)
Radiation therapy	
No	XX(XX.X)
Yes	XX(XX.X)
Adjuvant chemotherapy	
No	XX(XX.X)
Yes	XX(XX.X)
Previous treatment history (Fluoropyrimidine-based therapy)	
No	XX(XX.X)
Yes (refractory/intolerant)	XX(XX [XX/XX])
Previous treatment history (Oxaliplatin)	
No	XX(XX.X)
Yes (refractory/intolerant)	XX(XX [XX/XX])
Previous treatment history (Irinotecan)	
No	XX(XX.X)
Yes (refractory/intolerant)	XX(XX [XX/XX])
Previous treatment history (anti-EGFR)	
No	XX(XX.X)
Yes (refractory/intolerant)	XX(XX [XX/XX])
Breakdown*1	
Cetuximab	XX(XX.X)
Panitumumab	XX(XX.X)

	N(%)
Previous treatment history (anti-VEGF)	
No	XX(XX.X)
Yes (refractory/intolerant)	XX(XX [XX/XX])
Breakdown*2	
Bevacizumab	XX(XX.X)
Ramucirumab	XX(XX.X)
Aflibercept	XX(XX.X)
Number of enforced regimens (Palliative)	
1	XX(XX.X)
2	XX(XX.X)
3	XX(XX.X)
4	XX(XX.X)
5	XX(XX.X)
2 or less	XX(XX.X)
3 or more	XX(XX.X)

*1: Patient with previous treatment history (anti-EGFR)

*2: Patient with previous treatment history (anti-VEGF)

4.4. Collection of medication data

Subjects: SP (tissue) and SP (liquid)

The following information will be collected as basic data for the clinical trial treatment group:

- ❑ Number of treatment courses (N, median, minimum, maximum, first quartile, third quartile, mean, and standard deviation)
- ❑ Treatment period (N, median, minimum, maximum, first quartile, third quartile, mean, and standard deviation)
    - ❑ Definition of treatment period
        - • Patients who have been determined to terminate protocol treatment as of the data cutoff date

        (Treatment period) = (Protocol treatment termination determination date) − (Treatment start date of the first course) + 1
        - • Patients with ongoing protocol treatment as of data cutoff date

        (Treatment period) = (Data cutoff date) − (Treatment start date of the first course) + 1
- ❑ The proportion of patients with experience of administration postponement (number and proportion of patients who experienced postponement of administration even once through the treatment course, and the reason for postponement)
    - ➢ The proportion of patients with experience of administration postponement
    - ➢ Reason for administration postponement
        - ✧ Reason for postponement of pertuzumab administration
        - ✧ Reason for postponement of trastuzumab administration
- ❑ The proportion of patients with experience of pertuzumab withdrawal (number of patients who experienced pertuzumab withdrawal even once through the treatment course, and the reason for withdrawal)
    - ➢ The proportion of patients with experience of pertuzumab withdrawal
    - ➢ Reason for drug withdrawal
- ❑ The proportion of patients with experience of postponed administration or withdrawal of pertuzumab (number and proportion of patients who experienced postponement or withdrawal of pertuzumab even once through the treatment course)
    - ➢ The proportion of patients with experience of administration postponement or withdrawal of pertuzumab

Subjects: All enrolled cases (natural history group)

※ The following information will be collected in the natural history group and listed in a table:

- ❑ Regimen No.
- ❑ Names of treatment regimens
- ❑ Administration period (treatment start date and last administration date)
- ❑ Date of termination decision and reason for termination

In addition, data of up to 2 regimens will be collected in the natural history group.

4.4.1. Reason for termination of protocol treatment

Subjects: SP (tissue) and SP (liquid)

- ❑ Collection of treatment progress (treatment completion, ongoing treatment, and treatment termination)

- For the cases of protocol treatment termination, the number of cases corresponding to the following reason for termination will be collected, and the details of the reason for termination will be described:
    - Reason for termination
        - Progression of the underlying disease, adverse events, rejection by patient (not associated with adverse events), death during protocol treatment, physician review, etc.
    - Special notes on “details of the reason for termination related to adverse events”
        - Enrollment number, the reason for termination, protocol treatment duration, names of adverse events, and dates of the first onset of adverse events

4.4.2. Special notes

- No subgroup analysis will be performed on the medication data.

4.5. Efficacy analysis plan

4.5.1. 【Primary endpoint】Confirmed ORR assessed by investigator review (according to RECIST guideline ver1.1)

4.5.1.1. Main analysis method

Subjects: FAS (tissue-primary) and FAS (liquid-primary)

The following will be calculated and analyzed:

- Breakdown of the best overall effect (CR, PR, SD, PD, and NE)
- Review on whether there are more response cases (the cases in which the best overall effect is CR or PR) than the number of boundary response subjects
- Clopper–Pearson 95% confidence interval
- *P*-value when the threshold of 5% is the null hypothesis

4.5.1.2. Analysis method using a model

Subjects: FAS (tissue), FAS (liquid), all enrolled cases (tissue), and all enrolled cases (liquid)

- Odds ratio and its 95% Wald confidence interval determined using the univariate logistic regression with factors used in subgroup analysis as covariates

4.5.1.3. Other data collection and analysis

Subjects: FAS (tissue), FAS (liquid), all enrolled cases (tissue), all enrolled cases (liquid), and all enrolled cases (natural history group)

The following will be calculated and analyzed:

- Breakdown of the best overall effect (CR, PR, SD, PD, and NE)
- Response cases (cases in which the best overall effect is CR or PR)
- Clopper–Pearson 95% confidence interval

4.5.2. Confirmed ORR assessed by central review (according to RECIST guideline ver1.1)

※ If the central review is not conducted owing to cost, etc., the analysis may not be performed.

Subjects: FAS (tissue), FAS (liquid), all enrolled cases (tissue), all enrolled cases (liquid), and all enrolled cases (natural history group)

- Breakdown of the best overall effect (CR, PR, SD, PD, and NE)
- Number of response cases

- ❑ Clopper–Pearson 95% confidence interval
- ❑ Odds ratio and its 95% Wald confidence interval by univariate logistic model with factors used in subgroup analysis as covariates

4.5.3. Progression-free survival

The definition is as follows:

Start date	Event	Termination
Enrollment date	Progression or all deaths, whichever is earlier	The progression-free survival (PFS) and untraceable cases will be terminated at the final PFS survival confirmation date

※ For the cases in which a subject has died without imaging for a long time, whether "event on death date" or "terminated on final PFS confirmation date" will be determined at the case review meeting held before establishing the data.

Subjects: FAS (Tissue), FAS (Liquid), all enrolled cases (Tissue), all enrolled cases (Liquid), and all enrolled cases (natural history group)

- ❑ Kaplan–Meier curve (confidence intervals and confidence bands will not be plotted)
- ❑ Number and breakdown of events (death and progression)【Added in ver3.0】
- ❑ The proportion of patients with PFS at 3, 6, and 12 months and its 95% confidence interval based on Greenwood's formula
- ❑ Number at risk
- ❑ Median PFS and its Brookmeyer–Crowley 95% confidence interval
- ❑ Hazard ratio and its 95% Wald confidence interval by univariate Cox regression with factors used in the subgroup analysis as covariates

4.5.4. Duration of response

The definition is as follows:

Start date	Event	Termination
The day when CR or PR is first confirmed by the overall effect by RECIST ver1.1 (first response confirmation date)	Progression or all deaths, whichever is earlier	Sustained response and untraceable cases will be terminated at the final PFS confirmation date

※ For cases in which the subject has died without imaging for a long time, whether "event on death date" or "terminated on final PFS confirmation date" will be determined at the case review meeting held before establishing the data.

The subjects are the following 5 groups:

・Of FAS (tissue), cases in which a response is confirmed by the doctor review

・Of FAS (liquid), cases in which a response is confirmed by the doctor review

・Of all enrolled cases (tissue), cases in which a response is confirmed by the doctor review

・Of all enrolled cases (liquid), cases in which a response is confirmed by the doctor review

・Of all enrolled cases (natural history group), cases in which a response is confirmed by the doctor review

- ❑ Kaplan–Meier curve (confidence intervals and confidence bands will not be plotted)
- ❑ Number and breakdown of events (death and progression)【Added in ver3.0】
- ❑ The proportion of duration of response (DoR) cases at 3 and 6 months and its 95% confidence interval based on Greenwood's formula

- ❑ Number at risk
- ❑ Median DoR and its Brookmeyer–Crowley 95% confidence interval
- ❑ Hazard ratio and its 95% Wald confidence interval by univariate Cox proportional hazards model with factors used in subgroup analysis as covariates

4.5.5. Time to treatment failure

The definition is as follows:

Start date	Event	Termination
Enrollment date	Progression, all deaths, or termination of clinical trial treatment, whichever is earlier	The PFS cases with ongoing clinical trial treatment and untraceable cases will be terminated at the final PFS confirmation date.

Subjects: FAS (tissue), FAS (liquid), all enrolled cases (tissue), all enrolled cases (liquid), and all enrolled cases (natural history group)

- ❑ Kaplan–Meier curve (confidence intervals and confidence bands will not be plotted)
- ❑ Number and breakdown of events (death, progression, and discontinuation of clinical trial treatment)【Added in ver3.0】
- ❑ The proportion of time to treatment failure (TTF) cases at 3, 6, and 12 months and its 95% confidence interval based on Greenwood’s formula
- ❑ Number at risk
- ❑ Median TTF and its Brookmeyer–Crowley 95% confidence interval
- ❑ Hazard ratio and its 95% Wald confidence interval by univariate Cox proportional hazards model with factors used in subgroup analysis as covariates

4.5.6. Disease control rate

※ If the central review is not conducted owing to costs, etc., the disease control rate (DCR) of the central review may not be calculated.

Subjects: FAS (tissue), FAS (liquid), all enrolled cases (tissue), all enrolled cases (liquid), and all enrolled cases (natural history group)

- ❑ Number of DC cases by investigator/sub-investigator review or central review (combined number of cases in which the best overall effect is CR, PR, or SD)
- ❑ Clopper–Pearson 95% confidence interval
- ❑ Odds ratio and its 95% Wald confidence interval by univariate logistic regression with factors used in subgroup analysis as covariates

4.5.7. Overall survival

The definition is as follows:

Start date	Event	Termination
Enrollment date	All deaths	Surviving and untraceable cases will be terminated on the last confirmed survival date.

Subjects: FAS (tissue), FAS (Liquid), all enrolled cases (tissue), all enrolled cases (Liquid), and all enrolled cases (natural history group)

- ❑ Kaplan–Meier curve (confidence intervals and confidence bands will not be plotted)
- ❑ Number of events

- The proportion of overall survival (OS) cases at 3, 6, and 12 months and its 95% confidence interval based on Greenwood's formula
- Number at risk
- Median OS and its Brookmeyer–Crowley 95% confidence interval
- Hazard ratio and its 95% Wald confidence interval by univariate Cox regression with factors used in subgroup analysis as covariates
- Collection of the breakdown of death cases【Added in ver3.0】

4.5.8. Special notes

- The following subgroup analyses will be performed for efficacy endpoints:
  - Gender (male/female)
  - Age (under 70/70 or older)
  - ECOG PS (0/1)
  - Primary lesion site (right/left side)
  - BRAF status in OCP/OCA (wild type/mutant)
  - PIK3CA status in OCP/OCA (wild type/mutant)
  - RAS status in Guardant360 (wild type/mutant)
  - BRAF status in Guardant360 (wild type/mutant)
  - PIK3CA status in Guardant360 (wild type/mutant)
  - MSI status (MSS, MSI-L/MSI-H)
  - Number of metastatic organs (1 or less/2 or more)
  - Presence of primary lesion (Yes/No)
  - Number of previous treatment regimens (2 or less/3 or more)
  - Usage history of angiogenesis inhibitors (bevacizumab, ramucirumab, or aflibercept) (Yes/No)
  - Response by regimen including anti-EGFR antibody drug in previous treatment (Yes/No)
  - HER2 review in tumor tissue (positive/negative)
  - HER2 IHC score in tumor tissue (0, 1+, 2+, 3+)
  - FISH HER2/CEP17 ratio in tumor tissue (less/more than 2.0)
  - FISH HER2/CEP17 ratio in tumor tissue (less/more than 4.0)
  - FISH HER2/CEP17 ratio in tumor tissue (less/more than 6.0)
  - FISH HER2 copy number in tumor tissue (less/more than 9.5)
  - FISH HER2 copy number in tumor tissue (less/more than 15.0)
  - HER2 amplification in blood sample (Guardant360) (++/+++)

Based on the analysis results of the Translational Research (TR) study carried out as an additional study of this research, a subgroup analysis will be performed on the endpoints of efficacy for the following items. In the additional research, the analysis will be performed at the National Cancer Center; Aichi Cancer Center Research Institute; and Laboratory of Systems Genomics, Department of Computational Biology and Medical Sciences, Graduate School of Frontier Sciences, University of Tokyo. The DM group of OCRS will receive the

analysis result from the clinical trial-coordinating physician, and it will be stored on DVD-R together with the analysis data and transferred to the STAT group.

Since the following factors are not the data collected in the main study of the clinical trial, the analysis results will not be included in the Clinical Study Report.

- *HER2*-positive cell ratio (less/more than 50%)
- *HER2*-positive cell ratio (less/more than 30%)
- *HER2*-positive cell ratio (less/more than 10%)

4.6. Safety analysis plan【added or revised in ver3.0】

Subjects: SP (tissue) and SP (liquid)

※ The sum of sets will also be collected in the safety analysis.

4.6.1. Collection of all adverse events

The number of cases of occurrence, rate of occurrence, and number of adverse events that occurred during all cycles will be calculated. In addition, data will be collected by system organ class (SOC) and preferred terms (PTs).

- ❑ Collection of all adverse events
  - Adverse responses including those for which a causal relationship with protocol treatment can be denied
  - Adverse responses excluding those for which a causal relationship with protocol treatment can be denied
- ❑ Collection of an occurrence rate of 10% or more
  - Adverse responses including those for which a causal relationship with protocol treatment can be denied
  - Adverse responses excluding those for which a causal relationship with protocol treatment can be denied
- ❑ Collection of serious adverse events, termination of treatment, and adverse events leading to drug withdrawal ※1
  - Adverse responses including those for which a causal relationship with protocol treatment can be denied
  - Adverse responses excluding those for which a causal relationship with protocol treatment can be denied

※1 Definition of treatment termination and drug withdrawal

Quoted from Protocol 6.1

The clinical trial treatment in this study is the simultaneous combination therapy with the investigational drugs trastuzumab and pertuzumab. Pertuzumab is administered first, followed by post-administration observation for 60 min or longer. Subsequently, trastuzumab is administered for 90 min or longer, followed by post-administration observation for 60 min (the observation period can be shortened to 30 min after the second time as described in 6.1.1 and 6.1.2.). However, if only pertuzumab meets the criteria for termination, the investigator may consider the clinical trial treatment with trastuzumab as a single drug administration by discussing with the Clinical Trial Coordinating Committee. Pertuzumab will not be administered as a single drug because its clinical efficacy and safety have not been established in other cancer types.

The following terms are used in this Clinical Trial Implementation Plan:

- Termination: the administration of the study drug will not be resumed
- Withdrawal: the study drug is not administered for more than one administration schedule and proceeds to the next administration schedule.

4.6.2. Collection of the worst grade by adverse event items

4.6.2.1. Occurrence rate of adverse events

The following will be collected for the worst grade by adverse event items from the start of protocol treatment to that of post-treatment or death based on CTCAE v4.0-JCOG. They will also be collected for all grades and grade 3 or higher. In addition, the grading from the test values will not be performed, and the collection will be performed by SOC and PTs by using the grade of the institutional report.

- ❑ The worst grade by items related to all adverse events that occurred from the start of protocol treatment to that of post-treatment or death
  - ➢ Adverse responses including those for which a causal relationship with protocol treatment can be denied
  - ➢ Adverse responses excluding those for which a causal relationship with protocol treatment can be denied
- ❑ The worst grade related to all adverse events that occurred from the start of protocol treatment to that of post-treatment or death
  - ➢ Adverse responses including those for which a causal relationship with protocol treatment can be denied
  - ➢ Adverse responses excluding those for which a causal relationship with protocol treatment can be denied

4.6.2.2. Number of occurrences of adverse events

The following will be collected for the number of occurrence grade events by adverse event items from the start of protocol treatment to that of post-treatment or death based on CTCAE v4.0-JCOG. The collection will be performed by SOC and PTs by using the grade of the institutional report.

- ❑ The number of occurrence grade events by all adverse event items from the start of protocol treatment to that of post-treatment or death
  - ➢ Adverse responses including those for which a causal relationship with protocol treatment can be denied
  - ➢ Adverse responses excluding those for which a causal relationship with protocol treatment can be denied

4.6.3. Definitions of serious adverse events and other significant adverse events

<u>*Quoted from protocol 10.4.1*</u>

Definition of serious adverse events

An adverse event that falls under any of the following is a “serious adverse event”:

① Death or life-threatening

② Results in persistent or marked disability or dysfunction

③ Causes congenital abnormalities and birth defects

④ Requires hospitalization or extension of the hospitalization period

However, the following items are excluded:

－ Hospitalization and death due to progression of primary disease (PD)

－ Hospitalization or extension of hospitalization period for reducing the burden on subjects who receive a medical examination from a remote location

－ Pre-planned hospitalization or extension of hospitalization period

－ Hospitalization or extension of hospitalization period not related to adverse events

－ Less than 24 h of hospitalization or extension of the hospitalization period for follow-up only

⑤ A medically significant event; defined as an event that endangers the subjects or requires medical or surgical intervention to prevent the above consequences

<u>*Quoted from protocol 10.4.2*</u>

Events of clinical interest

“Cases requiring treatment owing to the reduction in asymptomatic LVEF or leading to the termination of clinical trial treatment” is an event of clinical interest even if the severity criteria are not met.

4.6.4. Special notes

- ❑ Subgroup analysis will not be performed for safety analysis
- ❑ Safety summary will be collected ※1
- ❑ Deaths from adverse events will be collected

※1 The number of occurrences of adverse events, deaths, serious adverse events, terminations due to adverse events, and adverse effects will be collected.

4.7. Other exploratory analyses

4.7.1. ORR of regimens, including an anti-EGFR antibody drug (cetuximab or panitumumab), in previous treatment

Subjects: FAS (tissue), FAS (liquid), all enrolled cases (tissue), and all enrolled cases (liquid)

- ❑ Breakdown of the best overall effect (CR, PR, SD, PD, and NE)
- ❑ Number of response cases (number of cases in which the best overall effect is CR or PR)
- ❑ Clopper–Pearson 95% confidence interval
- ❑ Odds ratio and its 95% Wald confidence interval by univariate logistic regression with subgroup as a covariate

4.7.2. Waterfall plot

Subjects: FAS (tissue), FAS (liquid), all enrolled cases (tissue), and all enrolled cases (liquid)

A waterfall plot will be created by calculating the best change rate (%) for the sum of tumor diameters of measurable lesions from the baseline based on full-image evaluation. All measurements collected after the baseline time point, at which an image evaluation is performed, will be used. In addition, the best change rate is the maximum shrinkage rate compared to baseline, or the minimum increase rate compared to the baseline for the cases without shrinkage. Herein, a graph with color-coded best overall effect (based on the review of the investigator or physician) and *HER2* IHC score (tumor tissue; 0, 1+, 2+/3+) will be created using RECIST v1.1 during the tracking period.

- Vertical axis of graph: the best rate of change in the sum of diameters of tumors (%)
- Horizontal axis of graph: cases (sorted from left with the best rate of change)

4.7.3. Spider plot

Subjects: FAS (tissue), FAS (liquid), all enrolled cases (tissue), and all enrolled cases (liquid)

A spider plot will be created by calculating the rate of change (%) for the sum of tumor diameters of measurable lesions from the baseline based on full-image evaluation. All measurements collected after the baseline time point, at which an image evaluation is performed, will be used. Herein, a graph will be created with color-coded best overall effect (based on the review of the investigator or physician) and *HER2* IHC score (tumor tissue; 0, 1+, 2+/3+) by using RECIST v1.1 during the tracking period.

- Vertical axis of graph: the rate of change in the sum of diameters of tumors (%)
- Horizontal axis of graph: the period starting from the clinical trial treatment start date

4.7.4. Swimmer plot

Subjects: FAS (tissue), FAS (liquid), all enrolled cases (tissue), and all enrolled cases (liquid)

A swimmer plot will be created by plotting the cases at progression and at confirmation of the best effect (the day when CR or PR was first confirmed in the cases with the best overall effect of CR or PR, or the day when SD or higher was confirmed in the cases of SD). The horizontal axis will correspond to the protocol treatment period. Herein, a graph will be created with color-coded best overall effect (based on the review of the investigator or physician) and *HER2* IHC score (tumor tissue; 0, 1+, 2+/3+) by using RECIST v1.1 during the tracking period.

- Vertical axis of graph: cases (sorted from the top in descending order of treatment period from the enrollment date)
- Horizontal axis of graph: the period starting from the clinical trial treatment start date

4.8. Post-treatment summary

Subjects: FAS (tissue), FAS (liquid), all enrolled cases (tissue), and all enrolled cases (liquid)

The following items will be collected and analyzed:

- Post-treatment
- Implementation of the following treatment regimens as post-treatment:
  - Regorafenib
  - Trifluridine and tipiracil hydrochloride
  - Investigational drug
  - Radiation therapy

- Surgery
- Other anticancer drugs

- The following information will be collected for the cases that have undergone post-treatment:
  - Items to be described for the cases that have undergone post-treatment
    - Enrollment number, post-treatment details, the period from the treatment start date to post-treatment start date

4.9. Analysis for the Clinical Study Report preparation

The following chapter structure will be used as a guideline for the items to be published in the Clinical Study Report, and the summary index will be calculated as necessary. In addition, the following is a guideline and may change during the collection.

＜Efficacy assessment [subjects: FAS (tissue), FAS (liquid), all enrolled cases (tissue), all enrolled cases (liquid), and all enrolled cases (natural history group)]. However, the results of FAS (tissue-primary) and FAS (liquid-primary) for the primary endpoint will also be listed.

- 11.2 Demographic data
- 11.4.1.1 Objective response rate (ORR)
- 11.4.1.2 Disease control rate (DCR)
- 11.4.1.3 Progression-free survival (PFS)
- 11.4.1.4 Duration of response (DoR)
- 11.4.1.5 Time to treatment failure (TTF)
- 11.4.1.6 Overall survival (OS)

＜Safety assessment [subjects: SP (tissue) and SP (liquid)]

- 12.2.1 Occurrence rate of adverse events (the number of cases by worst grade and the proportion of the cases)
- 12.4.2 Summary statistics and shifts in clinical test values

＜Form to be quoted, but not included in the body of the Clinical Study Report＞

- 14.3.2 List of deaths and other serious and significant adverse events
- 14.3.4 List of clinical test values by patient

＜Appendix＞

- 16.2.1 List of cases of clinical trial treatment termination
- 16.2.2 List of cases in which significant deviations from the clinical trial plan occurred
- 16.2.3 List of cases excluded from FAS
- 16.2.4a List of demographic data
- 16.2.4b Treatment history
- 16.2.4c List of combination drugs and therapies
- 16.2.5 Compliance (medication status)
- 16.2.6 List of efficacy response data by patient (tumor assessment, OS, PFS, post-treatment, and tumor markers）

- ❑ 16.2.7 List of adverse events by patient

5. Report of analysis results

The report of analysis results will be prepared and established based on the STAT group manual NCCHE-OCRS-SOP-Stat-001-4.

6. Inspection plan

Based on the STAT group manual NCCHE-OCRS-SOP-Stat-001-4, data analysis inspection operations are defined as follows.

In addition, refer to Chapter 7 of this SAP for the implementation system of each operation.

6.1. Data analysis inspection operations

6.1.1. Inspection range

- ❑ The data analysis operations will inspect all data analysis results specified in this SAP.
  - ➢ The operator and inspector will perform double programming in an independent environment and inspect data analysis results based on the STAT group manual NCCHE-OCRS-SOP-Stat-001-4.
  - ➢ Original inspection records on a paper medium will be stored in the security section of the NCCHE OCR.

6.1.2. Methods

The inspector will confirm that the values of the main output and sub-output printed on paper are identical.

6.1.3. Exceptions in inspection and their handling

There are no exceptions to be addressed in this study.

6.2. Operation to create the analysis result report

6.2.1. Inspection range

The results of all data analysis described in the analysis result report will be inspected.

6.2.2. Methods

Inspection operations will be performed based on the STAT group manual NCCHE-OCRS-SOP-Stat-001-4.

7. Implementation system

Shogo Nomura is a staff member of the STAT group in charge of statistical operations and is responsible for all operations. The persons in charge of data analysis operations and the creation of an analysis result report will be listed in the analysis result report.

8. Changes from the corresponding protocol

There are no changes from the corresponding protocol in this SAP.

9. Other considerations

9.1. Pharmacokinetic and biomarker analysis

Not applicable

9.2. Integrated analysis of natural history group, SCRUM-Japan Registry, and this clinical trial 【Added or revised in ver3.0】

This additional study will conduct comparative analysis by using past and concurrent controls as well as integrated analysis by using external data. The outline of the study is shown in this SAP, and its details are described in the Research Implementation Plan of this additional study.

【Added in ver3.0】

In consultation with the Clinical Trial Coordinating Committee, it has been decided that the integrated analysis will be performed in the clinical trial, not in this additional study, and the results will be reported in a Clinical Study Report according to section 12.12.1 of the protocol. Section 12.12.1 of the protocol states that “the analysis method will be specified in the SAP before establishing the data of the main analysis.” However, because the main analysis was performed ahead of schedule, the analysis method will be specified after establishing the data.

9.2.1. Data handling

The handling of data required for integrated analysis is defined below.

9.2.1.1. Clinical trial treatment group data in TRIUMPH study

The clinical trial treatment group data of the TRIUMPH study will be used by extracting the following data from the EDC managed by the Data Management Office, Research Planning Division, Clinical Research Support Office, National Cancer Center Hospital East:

- ❑ Background factors (same as the items collected in main research)
- ❑ Treatment data (same as the items collected in main research)
- ❑ Data required to calculate efficacy endpoints
  - ➢ Data required to calculate the response rate [the best overall effect and overall effect at each measurement time point (by doctor review)]
  - ➢ Data required to calculate PFS (start date, progression date, death date, and final PFS confirmation date)
  - ➢ Data required to calculate overall survival (start date, date of death, and final confirmed survival date)

9.2.1.2. Natural history group data in TRIUMPH study

The natural history group data of the TRIUMPH study will be used by extracting the data listed in 9.2.1.1. from the EDC managed by the Data Management Office, Research Planning Division, Clinical Research Support Office, National Cancer Center Hospital East.

9.2.1.3. Data extracted from SCRUM-Japan Registry

The SCRUM-Japan Registry data will be used by extracting the data needed to calculate background factors, treatment data, and efficacy endpoints (objective response rate, PFS, and overall survival) from the EDC managed by Data Management Office, Research Planning Division, Clinical Research Support Office, National Cancer Center Hospital East. However, because the details of the data that can be extracted have not been determined at the point of SAP ver1.0, the description is omitted. SAP will be updated when the data to be extracted and used is confirmed, and the handling of the data will be added.

※ The data registered by the establishment date of the TRIUMPH data set will be used.

※ Patients enrolled in the natural history group of the TRIUMPH study will be included only in the natural history group and will not be extracted from the SCRUM-Japan Registry data.

※ Data transfer procedure

- ✓ Before the transfer of data, those in charge of the DM in the TR Support Office and the STAT group will create a data definition document that corresponds to the variable names and definitions of the TRIUMPH study and SCRUM-Japan Registry data.
- ✓ After the data for creating the TRIUMPH study analysis report is established, the person in charge of the STAT group will request the person in charge of DM in the TR Support Office to send the SCRUM-Japan Registry data.

- ✓ After receipt of the request, the person in charge of DM in the TR Support Office will extract the relevant data from the EDC, save it on a suitable recording medium such as a CD-R together with the materials corresponding to the SDR, and send it to the person in charge of the STAT group.
- ✓ The person in charge of the STAT group will record the date of receipt of the SCRUM-Japan Registry data from the person in charge of DM in the TR Support Office as the "data reception date."
- ✓ The person in charge of the STAT group will confirm that the dataset received can be integrated with the TRIUMPH data set.

【Added in ver3.0】

The following data will be extracted from the SCRUM-Japan Registry data. Since the SCRUM-Japan unique ID is also stored in the EDC of the clinical trial, the SCRUM-Japan Registry can be linked to the data collected in the clinical trial. The data extraction period of the SCRUM-Japan Registry is scheduled for the end of June 2020.

- ❑ Patient identification number that can be linked to the data collected in the clinical trial
- ❑ SCRUM-Japan enrollment date
- ❑ Treatment start date of treatment line immediately after the patient became refractory or intolerant to all of 5-FU, oxaliplatin, irinotecan, and anti-EGFR antibody (cetuximab/panitumumab)
- ❑ Patient background factors
    - ➢ Background factors: gender; primary lesion site; tissue type; history of systemic chemotherapy before sample collection, anti-EGFR antibody drug administration before sample collection, and radiation therapy at the sample collection site
    - ➢ Treatment factors: collected by age, performance status, and treatment regimen
- ❑ The overall effect on the date of imaging and testing performed on the treatment line immediately after the patient became refractory or intolerant to 5-FU, oxaliplatin, irinotecan, and anti-EGFR antibody (cetuximab/panitumumab)
- ❑ The best overall effect, progression, progression review date, treatment end date, and death of the treatment line immediately after the patient became refractory or intolerant to 5-FU, oxaliplatin, irinotecan, and anti-EGFR antibody (cetuximab/panitumumab)
    - ➢ Date and cause of death in death cases
- ❑ Final confirmed survival date (only for surviving cases)

9.2.1.4. External data

The data of clinical trials of *HER2*-positive cases that have been or will be conducted overseas, which will be used in the additional studies, is referred to as "external data." However, because the use of external data is yet to be determined as of SAP ver1.0, the description is omitted. When the availability of external data is confirmed, SAP will be updated, and the handling of the data will be added.

【Added in ver3.0】

Integrated analysis with external data will not be performed.

9.2.2. Study design

The endpoints of this additional study are objective response rate, PFS period, and overall survival period. The definition of each endpoint follows the one in the main research. The following endpoints will be analyzed at each

analysis time point.

Analysis time point	End points
Main analysis	Objective response rate and PFS period
Final analysis	Objective response rate, PFS period, and overall survival period

Comparisons between the following 4 types of groups will be performed for the endpoints above. However, if the data integration is difficult or the sample size of a specific SCRUM-Japan Registry group is small, one of the following analyses may be omitted.

① Natural history group (concurrent control group) in the TRIUMPH study vs FAS of target patients based on tumor and blood samples of the clinical trial treatment group in the TRIUMPH study

② SCRUM-Japan Registry group vs FAS of the clinical trial treatment group in the TRIUMPH study

③ Concurrent control group and SCRUM-Japan Registry group (integrated control group 1) vs FAS of the clinical trial treatment group in the TRIUMPH study

④ Concurrent control group and group that integrates SCRUM-Japan Registry and external data (integrated study group 2) vs group that integrates FAS of the clinical trial treatment group in the TRIUMPH study and external data (integrated study group)

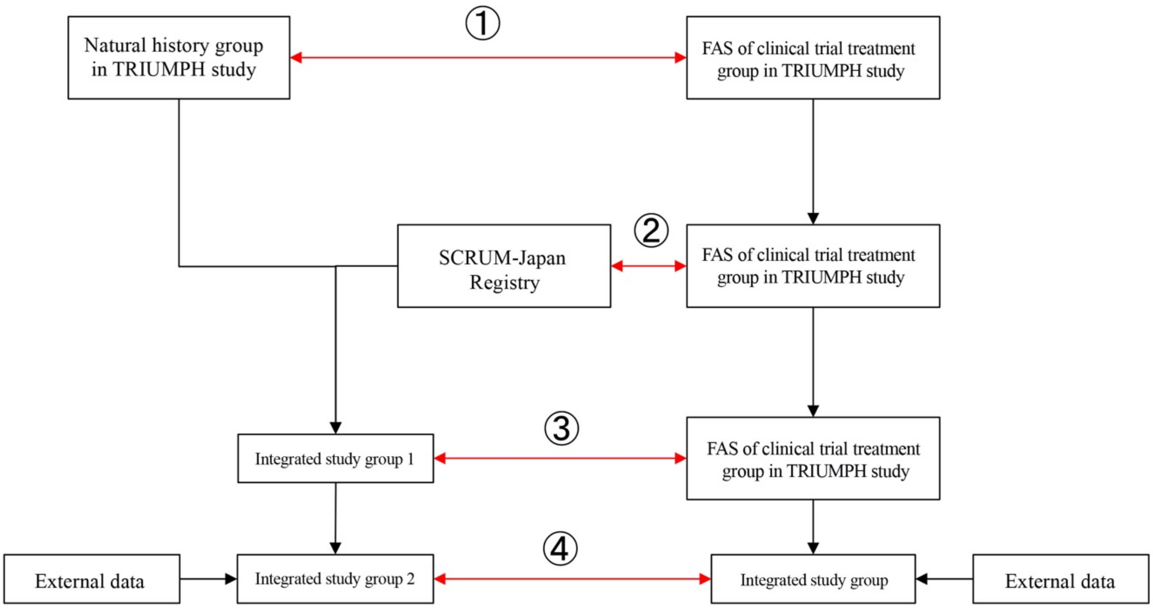

※Comparison will be made between the groups indicated by the red arrows in the figure.

【Added in ver3.0】

Since the sample size is not sufficiently large to perform multivariate analysis for confounding adjustment, the summary statistics below will be calculated for the data extracted from the SCRUM-Japan Registry, and the comparison of ② will be performed visually. As of January 15, 2020, no cases have been enrolled in the natural history group. Thus, ①, ③, and ④ will not be examined. If any patients are enrolled in the natural history group in the future, a list of data entered into the EDC will be included in the Clinical Study Report. The content of the

report will follow the description in the relevant sections of 9.2.3.

- ❑ Response rate for the SCRUM-Japan Registry group (only patients with informed consent) and its 95% confidence interval
- ❑ Survival curve of PFS and OS in the SCRUM-Japan Registry group (only patients with informed consent) and its summary statistics
- ❑ Response rate in the SCRUM-Japan Registry group (patients with informed consent and patients enrolled by opting-out) and its 95% confidence interval
- ❑ Survival curve for PFS and OS in the SCRUM-Japan Registry group (patients with informed consent and patients enrolled by opting-out) and its summary statistics

9.2.2.1. Analysis target group【Added or revised in ver3.0】

The analysis target group of each population is described below.

Group	Analysis target group
Clinical trial treatment group of TRIUMPH study	Of all cases enrolled in the TRIUMPH study, groups that meet eligibility criteria A and B, excluding subjects (ineligible cases) that meet any of the exclusion criteria
Natural history group of TRIUMPH study	Of all cases enrolled in the TRIUMPH study, groups that meet eligibility criteria A and do not meet any of the eligibility criteria B and the exclusion criteria
SCRUM-Japan Registry group	Of all cases enrolled in the SCRUM-Japan Registry, groups that meet all of the following:<br><br>1) *RAS* wild-type in tissue sample analysis<br>2) *HER2* positive in either tissue or blood sample analysis (※ in principle, the definition of *HER2* positive follows eligibility criteria A)<br>3) Refractory or intolerant to fluorinated pyrimidine antimetabolites, oxaliplatin, irinotecan, cetuximab, or panitumumab (regardless of treatment history of angiogenesis inhibitors such as bevacizumab, ramucirumab, and aflibercept as well as trifluridine/tipiracil hydrochloride, and regorafenib). In addition, for subjects who are under postoperative adjuvant chemotherapy or who have relapsed within 6 months after the last dose, postoperative adjuvant chemotherapy is counted as a treatment history.

9.2.3. Analysis plan for comparison【Added or revised in ver3.0】

【Added in ver3.0】

Analysis will not be performed using propensity scores. As needed, stratified analysis etc. will be subsequently performed using the treatment group (whether enrolled in the clinical trial) as explanatory variables for response rate, PFS, and OS, and each variable of patient background factors (age, gender, BMI, PS, left/right colon, tissue type, etc.) as a layer.

9.2.3.1. Calculation of propensity score

Analysis will be performed using propensity scores in the comparisons of ③ and ④ in 9.2.2. Here, the dataset excluding the outcome data (objective response rate, PFS period, and overall survival period) will be passed to the analyst who is not involved in the main analysis of the clinical trial and additional studies, and then propensity scores will be determined. Propensity scores will be calculated by multivariate logistic regression, with the binary variable of FAS of the clinical trial treatment group of SCRUM-Japan Registry group/TRIUMPH study as the response variable, and patient background factors (age, gender, BMI, PS, left and right colon, histological type, etc.) as explanatory variable candidates. The calculated propensity scores will be examined by creating a histogram or box-and-whisker plot and visually inspecting whether the propensity score distributions in both groups have sufficient overlap and whether there are outliers. When propensity score matching is performed, we will conduct an appropriate ratio of matching and compare the background factors between the two matched cohorts. If the values of standardized difference, etc. are large, the model constructed at the time of calculating the propensity score will be reviewed, the examination will be repeated to investigate whether the background factors are more balanced, and the final propensity score will be determined. The dataset containing the final propensity score will be passed to the person in charge of analysis involved in the main analysis of clinical trials and additional studies, and the analysis described in 9.2.3.2. to 9.2.3.4 below will be performed.

If a sufficient sample size cannot be obtained, analysis will not be performed using the propensity score. In addition, the patients with at least one defect in covariates used as explanatory variables in the propensity score calculation will be excluded from the subjects for the propensity score calculation.

The following analyses, including the one using propensity scores, will be performed for the endpoints in 9.2.3.2. to 9.2.3.4 for the comparisons of ③ and ④ in 9.2.2.

- Univariate analysis
- Multivariate analysis (adjusted by the factors used to build a model when generating the propensity score)
- Stratified analysis (stratified by propensity score)
- Multivariate analysis (adjusted by propensity score as an explanatory variable)
- Univariate analysis by IPW (standard weight) by using propensity score
- Univariate analysis by IPW (stabilized weight) by using propensity score
- Univariate analysis by propensity score matching

9.2.3.2. Objective response rate

The use of the results that require confirmation to review the best overall effect is preferred. However, because of problems such as image assessment not performed on a fixed schedule in the SCRUM-Japan Registry group, ORR may contain bias when using the best overall effect that requires confirmation. Therefore, the following analysis will be performed on the ORR based on the review of the best overall effect that does not require confirmation. In addition, when the central review is performed, the same analysis may be performed on the ORR based on the central review. Moreover, collection for each treatment line may be performed on ORR ※1.

※1 The best overall effect of treatment line immediately after the patient became refractory or intolerant to all of 5-FU, oxaliplatin, irinotecan, and anti-EGFR antibody (cetuximab/panitumumab)

- ❑ Clinical trial treatment group of the TRIUMPH study

- Breakdown of the best overall effect (CR, PR, SD, PD, and NE)
- Response rate and its Clopper–Pearson 95% confidence interval
- ORR of previous treatment will be collected for each treatment regimen

❑ Natural history group of the TRIUMPH study
- Breakdown of the best overall effect (CR, PR, SD, PD, and NE)
- Response rate and its Clopper–Pearson 95% confidence interval

❑ SCRUM-Japan Registry group (excluding groups enrolled by opting-out)
- Breakdown of the best overall effect (CR, PR, SD, PD, and NE)
- Response rate and its Clopper–Pearson 95% confidence interval

❑ SCRUM-Japan Registry group (including groups enrolled by opting-out)
- Breakdown of the best overall effect (CR, PR, SD, PD, and NE)
- Response rate and its Clopper–Pearson 95% confidence interval

9.2.3.3. PFS

The definition is as follows. However, since the treatment line may differ depending on the test, the analysis with a specific treatment start date as the start date will be performed as a sensitivity analysis. In addition, when the central review is performed, the same analysis will be carried out for the PFS after the central review.

Start date	Event	Termination
Specific treatment start date ※1	Progression or all deaths, whichever is earlier	The PFS and untraceable cases will be terminated at the final PFS confirmation date.

※1 Treatment start date of treatment line immediately after the patient became refractory or intolerant to all of 5-FU, oxaliplatin, irinotecan, and anti-EGFR antibody (cetuximab/panitumumab)

❑ Clinical trial treatment group of TRIUMPH study

※ The following items for clinical trial treatment and previous treatment will be output:
- Kaplan–Meier curve (confidence intervals and confidence bands will not be plotted)
- Number of events
- Proportion of PFS cases at 3, 6, and 12 months and its 95% confidence interval based on Greenwood's formula
- Number at risk
- Median PFS and its Brookmeyer–Crowley 95% confidence interval

❑ Natural history group of TRIUMPH study
- Kaplan–Meier curve (confidence intervals and confidence bands will not be plotted)
- Number of events
- Proportion of PFS cases at 3, 6, and 12 months and its 95% confidence interval based on Greenwood's formula
- Number at risk
- Median PFS and its Brookmeyer–Crowley 95% confidence interval

❑ SCRUM-Japan Registry group (excluding groups enrolled by opting-out)
- Kaplan–Meier curve (confidence intervals and confidence bands will not be plotted)
- Number of events

- Proportion of PFS cases at 3, 6, and 12 months and its 95% confidence interval based on Greenwood's formula
- Number at risk
- Median PFS and its Brookmeyer–Crowley 95% confidence interval

❑ SCRUM-Japan Registry group (including groups enrolled by opting-out)
- Kaplan–Meier curve (confidence intervals and confidence bands will not be plotted)
- Number of events
- Proportion of PFS cases at 3, 6, and 12 months and its 95% confidence interval based on Greenwood's formula
- Number at risk
- Median PFS and its Brookmeyer–Crowley 95% confidence interval

9.2.3.4. Overall survival

The definition is as follows. However, since the treatment line may differ depending on the test, the analysis with a specific treatment start date as the start date will be performed as a sensitivity analysis.

Start date	Event	Termination
Specific treatment start date ※1	All deaths	Surviving and untraceable cases will be terminated on the last confirmed survival date.

※1 Treatment start date of treatment line immediately after the patient became refractory or intolerant to all of 5-FU, oxaliplatin, irinotecan, and anti-EGFR antibody (cetuximab/panitumumab)

❑ Kaplan–Meier curve (confidence intervals and confidence bands will not be plotted)

❑ Number of events

❑ Proportion of PFS cases at 3, 6, and 12 months and its 95% confidence interval based on Greenwood's formula

❑ Number at risk

❑ Median OS and its Brookmeyer–Crowley 95% confidence interval

10. References

There are no additional references other than those listed in the protocol.

11. Special notes on analysis

11.1.【Added in ver2.0】Main analysis

The main analysis was to be performed after the number of FAS cases reaches 25, which is the sample size in both groups of "patients confirmed to be *HER2* positive in tumor tissues (A + B)" and "patients confirmed to be *HER2* positive and *RAS* wild-type in blood samples (A + C)." However, we have confirmed by monitoring that the number of response subjects, which is the boundary of the primary endpoint (confirmed ORR assessed by investigator review), is at least 5 in both the groups at the stage with 17 A + B cases and 15 A + C cases of possible tumor assessment, before reaching the subject size. Therefore, for early result announcement by the decision of the clinical trial coordinating physicians, the main analysis will be conducted on 17 A + B cases and 15 A + C cases, for which tumor assessment has been performed after the start of protocol treatment, by January 7, 2019 (data cutoff date).

11.1.1.【Added in ver2.1】Subgroup analysis of efficacy endpoint

In addition to the factors described in section 4.5.8, a subgroup analysis of the following items will be performed.

In addition, a color-coded plot with the gene mutations described in the following items will be created for sections 4.7.2, 4.7.3, and 4.7.4.

- *RAS*, *BRAF*, and *PIK3CA* mutations in Guardant360 (all wild/positive for at least one)

By using a part of the data collected in the GOZILA study (*ERBB2* mutation), we will perform a subgroup analysis for the endpoint of efficacy on the following items. In addition, a color-coded plot with the gene mutations described in the following items will be created for sections 4.7.2, 4.7.3, and 4.7.4. In addition, in the GOZILA study, data collection and management are being performed by the TR Support Office, and the OCRS Stat Group will receive a dataset on a recording medium, which contains the subject ID of the TRIUMPH study, the ID of the GOZILA study, and *ERBB2* mutation status, from the TR Support Office for this analysis.

Moreover, the following factors contain the data that are not collected in the main study of the clinical trial, and its analysis results will not be included in the Clinical Study Report.

- *RAS*, *BRAF*, *PIK3CA*, and *ERBB2* mutation in Guardant360 (all wild/positive for at least one)

11.2. 【Added in ver3.1】Finaly analysis

11.2.1. Presentation of results from TRIUMPH

In the case review meeting for the final analysis, it was confirmed that all enrolled cases and FAS were the same. Therefore, only the results of the FAS analysis will be included in the statistical analysis report (SAR) and the Clinical Study Report.

11.2.2. Imputation of missing data in SCRUM-Japan Registry

Missing data will not be imputed.

EOD
