# Supplementary material for: Circulating tumor DNA-guided treatment with pertuzumab plus trastuzumab for HER2-amplified metastatic colorectal cancer: a phase 2 trial
Source: Nat Med. 2021 Nov 11;27(11):1899–903. doi: 10.1038/s41591-021-01553-w (PMC8604726; doi:10.1038/s41591-021-01553-w)
Supplement: Supplementary file 2 — Reporting Summary [file 41591_2021_1553_MOESM2_ESM.pdf]

nature portfolio

Corresponding author(s): Takayuki Yoshino

Last updated by author(s): Sep 18, 2021

Reporting Summary

Nature Portfolio wishes to improve the reproducibility of the work that we publish. This form provides structure for consistency and transparency in reporting. For further information on Nature Portfolio policies, see our Editorial Policies and the Editorial Policy Checklist.

Statistics

For all statistical analyses, confirm that the following items are present in the figure legend, table legend, main text, or Methods section.

n/a	Confirmed	
☐	☒	The exact sample size (n) for each experimental group/condition, given as a discrete number and unit of measurement
☐	☒	A statement on whether measurements were taken from distinct samples or whether the same sample was measured repeatedly
☐	☒	The statistical test(s) used AND whether they are one- or two-sided<br>*Only common tests should be described solely by name; describe more complex techniques in the Methods section.*
☐	☒	A description of all covariates tested
☐	☒	A description of any assumptions or corrections, such as tests of normality and adjustment for multiple comparisons
☐	☒	A full description of the statistical parameters including central tendency (e.g. means) or other basic estimates (e.g. regression coefficient) AND variation (e.g. standard deviation) or associated estimates of uncertainty (e.g. confidence intervals)
☐	☒	For null hypothesis testing, the test statistic (e.g. F, t, r) with confidence intervals, effect sizes, degrees of freedom and P value noted<br>*Give P values as exact values whenever suitable.*
☐	☒	For Bayesian analysis, information on the choice of priors and Markov chain Monte Carlo settings
☐	☒	For hierarchical and complex designs, identification of the appropriate level for tests and full reporting of outcomes
☐	☒	Estimates of effect sizes (e.g. Cohen's d, Pearson's r), indicating how they were calculated

Our web collection on statistics for biologists contains articles on many of the points above.

Software and code

Policy information about availability of computer code

Data collection	Data were collected using Medidata Rave version 2015.1.1 and Medidata Classic Rave version 2018.2.0.<br>Ion ReporterTM Software version 4.4 (for OCA v1) and 5.0 (for OCA v3), Guardant360 (version 2.10 and 2.11), RTA software version 2.12, bcl2fastq version 2.19., BWA-MEM version 0.7.15 (arXiv:1303.3997v2).
Data analysis	SAS (version 9.4)

For manuscripts utilizing custom algorithms or software that are central to the research but not yet described in published literature, software must be made available to editors and reviewers. We strongly encourage code deposition in a community repository (e.g. GitHub). See the Nature Portfolio guidelines for submitting code & software for further information.

Data

Policy information about availability of data

All manuscripts must include a data availability statement. This statement should provide the following information, where applicable:
- Accession codes, unique identifiers, or web links for publicly available datasets
- A description of any restrictions on data availability
- For clinical datasets or third party data, please ensure that the statement adheres to our policy

To protect the privacy and confidentiality of patients in this study, clinical data are not made publicly available in a repository or the supplementary material of the article, but will be available at any time reasonable request to the Corresponding author. Those requests will be reviewed by a study steering committee to verify whether the request is subject to any intellectual property or confidentiality obligations. All data shared will be de-identified.

Field-specific reporting

Please select the one below that is the best fit for your research. If you are not sure, read the appropriate sections before making your selection.

☒ Life sciences ☐ Behavioural & social sciences ☐ Ecological, evolutionary & environmental sciences

For a reference copy of the document with all sections, see nature.com/documents/nr-reporting-summary-flat.pdf

Life sciences study design

All studies must disclose on these points even when the disclosure is negative.

Sample size	The planned sample size for each testing group (tissue-positive and ctDNA-positive) was calculated to be 25 on the basis of a power of 90% to test the null hypothesis of confirmed ORR by investigator assessment of 5%, versus the alternative hypothesis of the ORR of 30%, at a one-sided α of 0.025. Five confirmed objective responses were needed to declare the study positive. No adjustment for multiplicity was planned.
Data exclusions	No data were excluded from the analysis.
Replication	This study was a clinical study with human participants. Here, replication of data set is not applicable for our study.
Randomization	Randomization was not applicable because this study was a single-arm phase 2 trial.
Blinding	Blinding was not applicable because this study was a single-arm phase 2 trial.

Reporting for specific materials, systems and methods

We require information from authors about some types of materials, experimental systems and methods used in many studies. Here, indicate whether each material, system or method listed is relevant to your study. If you are not sure if a list item applies to your research, read the appropriate section before selecting a response.

Materials & experimental systems

n/a	Involved in the study	
☐	☒	Antibodies
☒	☐	Eukaryotic cell lines
☒	☐	Palaeontology and archaeology
☒	☐	Animals and other organisms
☐	☒	Human research participants
☐	☒	Clinical data
☒	☐	Dual use research of concern

Methods

n/a	Involved in the study	
☒	☐	ChIP-seq
☒	☐	Flow cytometry
☒	☐	MRI-based neuroimaging

Antibodies

Antibodies used	PATHWAY HER2/neu (4B5) Rabbit Monoclonal Primary Antibody (Ventana Medical Systems, Tucson, AZ), Lot Number: Y03230, Y21121, E13637, E33040.
Validation	This antibody has already been validated as an in vitro diagnostics and commercialized.

Human research participants

Policy information about studies involving human research participants

Population characteristics	Population characteristics are detailed in Extended Data Fig. 4. Eligible patients were male or female aged 20 years or older; had histologically confirmed mCRC; had an Eastern Cooperative Oncology Group (ECOG) performance status (PS) of 0 or 1; had RAS wild-type and HER2-positive tumors, defined as immunohistochemistry (IHC) 3+ >10% of tumor cells or fluorescence in situ hybridization (FISH) positive (HER2/CEP17 ratio ≥2.0) by tissue testing, or as HER2-amplified and RAS wild-type by ctDNA analysis; and were refractory or intolerant to a fluoropyrimidine, irinotecan, oxaliplatin, and an anti-EGFR antibody.
Recruitment	Patients were recruited by investigators based on prespecified inclusion/exclusion criteria.
Ethics oversight	Study protocol was approved by the institutional review board at each institution, and all patients provided written informed consent. Name of participating institutions are following:<br>1. National Cancer Center Hospital East, Kashiwa, Japan<br>2. Aichi Cancer Center Hospital, Nagoya, Japan<br>3. National Cancer Center Hospital, Tokyo, Japan

4. National Hospital Organization Kyushu Cancer Center, Fukuoka, Japan
5. Hokkaido University Hospital, Sapporo, Japan
6. National Hospital Organization Shikoku Cancer Center, Matsuyama, Japan
7. National Hospital Organization Osaka National Hospital, Osaka, Japan

Note that full information on the approval of the study protocol must also be provided in the manuscript.

Clinical data

Policy information about clinical studies

All manuscripts should comply with the ICMJE guidelines for publication of clinical research and a completed CONSORT checklist must be included with all submissions.

Clinical trial registration	UMIN000027887
Study protocol	The protocol has been submitted along with the manuscript.
Data collection	Data were entered into the EDC by the investigators and clinical research coordinators at each local site (National Cancer Center Hospital East, Aichi Cancer Center Hospital, National Cancer Center Hospital, National Hospital Organization Kyushu Cancer Center, Hokkaido University Hospital, National Hospital Organization Shikoku Cancer Center, and National Hospital Organization Osaka National Hospital). Participants were enrolled in TRIUMPH between January 24, 2018 and July 29, 2019.
Outcomes	The primary endpoint was confirmed objective response rate (ORR; defined as the proportion of patients who achieved a complete or partial response confirmed on a follow-up scan ≥4 weeks after the initial response) by investigator assessment. Secondary endpoints were progression-free survival (PFS), duration of response (DOR), disease control rate (DCR), overall survival (OS), confirmed ORR by independent central review, and incidences of adverse events.
